# Bmi‐1 Epigenetically Orchestrates Osteogenic and Adipogenic Differentiation of Bone Marrow Mesenchymal Stem Cells to Delay Bone Aging

**DOI:** 10.1002/advs.202404518

**Published:** 2024-09-03

**Authors:** Jingyu Zhao, Ao Chen, Rong Wang, Dong Qiu, Haiyun Chen, Jiyu Li, Jin'ge Zhang, Tianxiao Wang, Yue Wang, Yujie Lin, Jiawen Zhou, Yifei Du, Hua Yuan, Yongjie Zhang, Dengshun Miao, Yuli Wang, Jianliang Jin

**Affiliations:** ^1^ Department of Human Anatomy Research Centre for Bone and Stem Cells School of Basic Medical Sciences Key Laboratory for Aging & Disease School of Biomedical Engineering and Informatics Nanjing Medical University Nanjing Jiangsu 211166 China; ^2^ Department of Oral and Maxillofacial Surgery The Affiliated Stomatological Hospital of Nanjing Medical University State Key Laboratory Cultivation Base of Research Prevention and Treatment for Oral Diseases Jiangsu Province Engineering Research Centre of Stomatological Translational Medicine Nanjing Medical University Nanjing Jiangsu 210029 China; ^3^ School of Pharmacy Nanjing Medical University Nanjing Jiangsu 211166 China

**Keywords:** Bmi‐1, bone marrow mesenchymal stem cells, DNA methylation, DNMT3A, H3K27 trimethylation, senile osteoporosis

## Abstract

With the increase in the aging population, senile osteoporosis (SOP) has become a major global public health concern. Here, it is found that Prx1 and Bmi‐1 co‐localized in trabecular bone, bone marrow cavity, endosteum, and periosteum. *Prx1*‐driven *Bmi‐1* knockout in bone‐marrow mesenchymal stem cells (BMSCs) reduced bone mass and increased bone marrow adiposity by inhibiting osteoblastic bone formation, promoting osteoclastic bone resorption, downregulating the proliferation and osteogenic differentiation of BMSCs, and upregulating the adipogenic differentiation of BMSCs. However, *Prx1*‐driven *Bmi‐1* overexpression showed a contrasting phenotype to *Prx1*‐driven *Bmi‐1* knockout in BMSCs. Regarding mechanism, Bmi‐1‐RING1B bound to DNMT3A and promoted its ubiquitination and inhibited DNA methylation of *Runx2* at the region from 45047012 to 45047313 bp, thus promoting the osteogenic differentiation of BMSCs. Moreover, Bmi‐1‐EZH2 repressed the transcription of *Cebpa* by promoting H3K27 trimethylation at the promoter region −1605 to −1596 bp, thus inhibiting the adipogenic differentiation of BMSCs. It is also found that *Prx1*‐driven *Bmi‐1* overexpression rescued the SOP induced by *Prx1*‐driven *Bmi‐1* knockout in BMSCs. Thus, Bmi‐1 functioned as a hub protein in the epigenetic regulation of BMSCs differentiation to delay bone aging. The *Prx1*‐driven *Bmi‐1* overexpression in BMSCs can be used as an approach for the translational therapy of SOP.

## Graphical abstract

Bmi‐1 epigenetically orchestrates osteogenic and adipogenic differentiation of bone‐marrow mesenchymal stem cells (BMSCs) to prevent senile osteoporosis (SOP). *Prx1*‐driven *Bmi‐1* overexpression in BMSCs rescues the SOP phenotype induced by *Prx1*‐driven *Bmi‐1* knockout in BMSCs. The *Prx1*‐driven *Bmi‐1* overexpression in BMSCs can be used as an approach for the translational therapy of SOP. (Image created with BioRender.com).

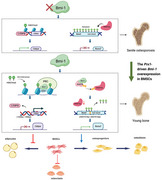

## 1 Introduction

Senile osteoporosis (SOP) is a systemic and progressive disease characterized by cellular senescence, a decrease in bone density, degeneration of bone microstructure, and an increased risk of bone fragility and fracture risk.^[^ ^1^ ^]^ Unlike postmenopausal osteoporosis, SOP is primarily caused by the process of aging and therefore affects both older males and females’ health.^[^ ^2^ ^]^ The global incidence of SOP is estimated to be 40% in elderly women and 30% in elderly men, respectively.^[^ ^3^ ^]^ Hence, it is critical to thoroughly understand the cellular and molecular mechanisms underlying SOP to identify new therapeutic targets.

In addition to the imbalance between bone formation by osteoblasts and bone resorption by osteoclasts, evidence shows that changes in the proliferative and differentiative functions of bone‐marrow mesenchymal stem cells (BMSCs) are also key factors in the development of SOP.^[^ ^4^ ^]^ Previous studies have indicated that BMSCs typically differentiate uniformly into osteoblasts, chondrocytes, and adipocytes; however, in older individuals, BMSCs differentiate more into adipocytes than into osteoblasts. This transformation in the differentiation properties of BMSCs reduces bone formation, consequently leading to SOP.^[^ ^2^, ^5^ ^]^ Peroxisome proliferator‐activated receptor γ (PPARγ) and CCAAT enhancer‐binding protein alpha (C/EBPα) are the main regulatory factors of adipocyte formation and differentiation, while Osterix (encoded by *Sp7*) and Runx family transcription factor 2 (Runx2) are the primary regulatory factors of osteogenic differentiation of BMSCs.^[^ ^6^ ^]^ However, the mechanisms underlying the abnormal differentiation of BMSCs in the elderly population remain poorly understood.

Epigenetics refers to heritable changes in gene expression and regulation that do not involve alterations in DNA sequences. Epigenetics plays a critical role in the physiological regulation of development, cell fate, cell proliferation, genome integrity, and fundamental transcriptional regulation.^[^ ^7^, ^8^ ^]^ It primarily involves DNA/RNA methylation, histone modifications, and the regulation of non‐coding RNA molecules. DNA methyltransferases play an important role in bone biology, and DNA methylation inhibitors can disrupt the osteogenic process. The 5‐azacytidine/5‐aza‐2′‐deoxycytidine (5‐Aza‐C/5‐Aza‐dC) can demethylate the genome, enhance the expression of osteogenesis‐related genes, and effectively promote osteogenic differentiation.^[^ ^9^, ^10^ ^]^ Nevertheless, it remains unclear whether DNA methylation influences the osteogenic differentiation of BMSCs. Moreover, although several studies have reported epigenetic modification during the differentiation processes of BMSCs, there is a notable lack of further investigations into the key molecules that epigenetically orchestrate osteogenic and adipogenic differentiation of BMSCs to delay bone aging.

B cell‐specific Maloney murine leukemia virus insertion region 1 (Bmi‐1), a member of polycomb repressive complex (PRC) 1, plays a crucial role in regulating the cell cycle and preventing cellular senescence.^[^ ^11^, ^12^, ^13^, ^14^ ^]^ We previously reported that mice with global knockout of *Bmi‐1* developed premature SOP phenotype and showed bone growth retardation, reduced bone formation, and increased adipocyte formation.^[^ ^15^ ^]^ However, it remains unclear whether this phenotype primarily arises from the impaired biological characteristics of Bmi‐1 in epigenetically maintaining BMSCs. Because Bmi‐1 significantly activates E3 ubiquitin ligase activity of ring finger protein RING1B, and the PRC1 complex formed by Bmi‐1 and RING1B plays a crucial role in histone ubiquitination.^[^ ^16^, ^17^, ^18^ ^]^ Our recent study demonstrates that Bmi‐1‐RING1B complex promotes GATA binding protein 4 (GATA4) ubiquitination and its selective autophagic degradation, thereby preventing cellular senescence, senescence‐associated secretory phenotype (SASP), and muscle dysfunction.^[^ ^19^, ^20^ ^]^ A previous study indicates that DNA methyltransferase 3 alpha (DNMT3A) is ubiquitinated by ring finger protein 180 and subsequently subjected to proteasome‐mediated degradation.^[^ ^21^ ^]^ During the osteogenic differentiation of BMSCs, the level of *Runx2* methylation level decreases, indicating that *Runx2* methylation plays a key regulatory role in osteoblast differentiation.^[^ ^10^, ^22^ ^]^ However, it remains to be elucidated whether Bmi‐1‐RING1B complex can promote the ubiquitin‐mediated degradation of DNMT3A, thereby inhibiting the DNA methylation of *Runx2* for upregulating osteogenic differentiation. Furthermore, enhancer of zeste homolog 2 (EZH2) is a component of the PRC2 complex, which mediates the trimethylation of H3K27 (histone 3) to generate H3K27me3.^[^ ^23^ ^]^ H3K27me3 is involved in the repression of numerous genes associated with cell development and differentiation;^[^ ^24^ ^]^ it promotes osteoblasts proliferation and plays a dual role in bone formation.^[^ ^25^, ^26^ ^]^ However, its effect on the adipogenic differentiation of BMSCs remains unclear. Although Bmi‐1 can enhance EZH2 expression and H3K27 trimethylation,^[^ ^27^, ^28^ ^]^ further investigations are needed to determine whether Bmi‐1 can promote the transcription of *Cebpa* and/or *Pparg* by enhancing H3K27 trimethylation for downregulating adipogenic differentiation of BMSCs.

Pair‐related homeobox 1 (Prx1) is a transcriptional coactivator that is expressed during limb sprout development, and *Prx1‐Cre* was employed to knock‐out genes in BMSCs.^[^ ^29^ ^]^ In the present study, to clarify the co‐localization of Prx1 and Bmi‐1 in long bone, we constructed a *tdTOMATO^+^Prx1‐cre* mouse model. To investigate the role of Bmi‐1 in regulating the biological characteristics of BMSCs in vivo, we generated mice with a conditional knockout of *Bmi‐1* in BMSCs (*Bmi‐1^f/f^Prx1‐cre*) and transgenic mice with *Prx1*‐driven *Bmi‐1* overexpression in BMSCs (*Bmi‐1^Tg^*). This study demonstrated that Bmi‐1‐RING1B complex promoted DNMT3A ubiquitination to inhibit DNA methylation of *Runx2* for upregulating osteogenic differentiation. Bmi‐1‐EZH2 repressed the transcription of *Cebpa* and *Pparg* by promoting H3K27 trimethylation at the promoter region for downregulating adipogenic differentiation of BMSCs. These synergistic effects of Bmi‐1 induced the differentiation of BMSCs into osteoblasts rather than into adipocytes, thus providing a new target to prevent SOP.

## 2 Results

### 2.1 Bmi‐1 Might Function as a Hub Protein in the Epigenetic Regulation of the Differentiation of BMSCs with Aging

**Figure 1:**
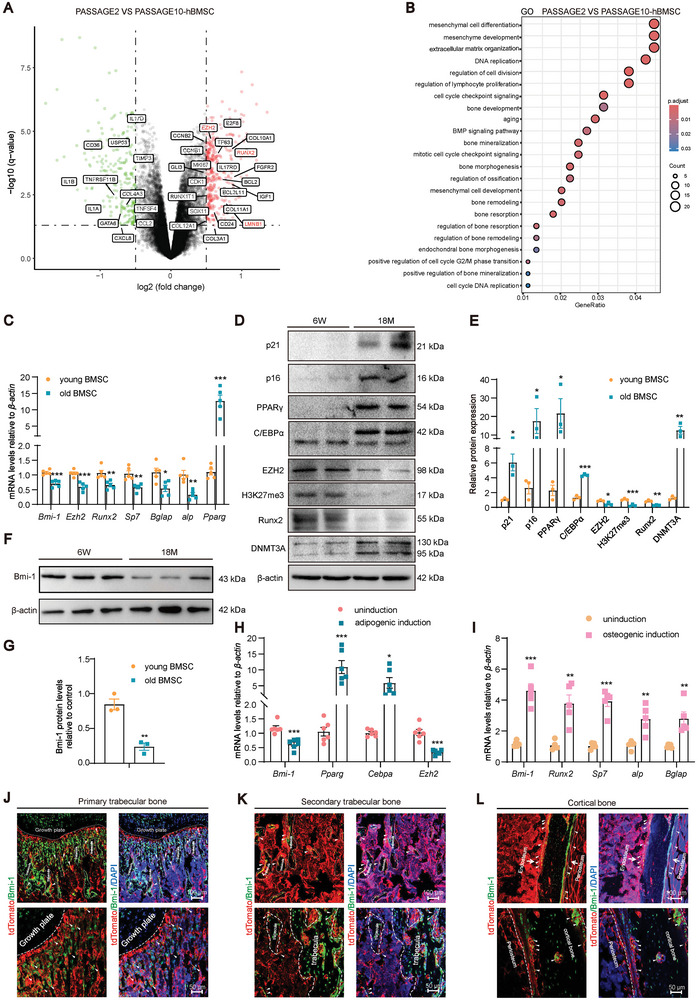
Bmi‐1 might act as a hub protein in the epigenetic regulation of differentiation of BMSCs with aging. A) Volcano plot analysis of the DEGs in young (Passage 2) and aged (Passage 10) human BMSCs. B) GO analysis of DEGs in these BMSCs. C) Primary second‐generation cells of young BMSCs extracted from 6‐week‐old wild‐type (WT) mice and primary sixth generation cells of aged BMSCs extracted from 18‐month‐old WT mice. The mRNA levels of *Bmi‐1, Ezh2, Runx2, Sp7, Bglap, alp*, and *Pparg* in BMSCs detected by RT‐qPCR and calculated and expressed relative to control *β‐actin* mRNA. Cell experiments were performed with five biological replicates per group. Values are expressed as mean ± SEM from five determinations per group, ^*^ *P* < 0.05, ^**^ *P* < 0.01, ^***^ *P* < 0.001, compared to the young BMSC group, unpaired Student's *t*‐test. D) Western blotting assay of BMSC extracts showing the protein levels of p21, p16, PPARγ, C/EBPα, EZH2, H3K27me3, Runx2, and DNMT3A. β‐Actin was used as a loading control. E) Protein expression relative to β‐actin was assessed by densitometric analysis. F) Western blotting assay of BMSC extracts showing the protein level of Bmi‐1. β‐Actin was used as a loading control. G) Bmi‐1 protein expression relative to β‐actin was assessed by densitometric analysis. Cell experiments were performed with three biological replicates per group. Values are expressed as mean ± SEM from three determinations per group, ^*^ *P* < 0.05, ^**^ *P* < 0.01, ^***^ *P* < 0.001 compared to the young BMSC group, unpaired Student's *t*‐test. H) BMSCs extracted from WT mice that were induced or not induced for adipogenic differentiation. The mRNA levels of *Bmi‐1*, *Pparg*, *Cebpa*, and *Ezh2* in BMSCs detected by RT‐qPCR and calculated and expressed relative to control *β‐actin* mRNA. I) BMSCs extracted from WT mice that were induced or not induced for osteogenic differentiation. The mRNA levels of *Bmi‐1*, *Runx2*, *Sp7*, *alp*, and *Bglap* in BMSCs detected by RT‐qPCR and calculated and expressed relative to control *β‐actin* mRNA. Cell experiments were performed with five biological replicates per group. Values are expressed as mean ± SEM from five determinations per group, ^*^ *P* < 0.05, ^**^ *P* < 0.01, ^***^ *P* < 0.001, compared to the young BMSC group, unpaired Student's *t*‐test. J–L) Representative micrographs of Prx1 and Bmi‐1 immunofluorescence‐labeled cells in the primary trabecular bone, secondary trabecular bone, and cortical bone of the femur from 8‐week‐old *tdTOMATO^+^Prx1‐cre* male mice. Red fluorescence represents Prx‐1; green fluorescence represents Bmi‐1; DAPI was used for staining the nuclei.

To determine the biology characteristics of BMSCs with aging and the possible regulatory mechanisms, the differentially expressed genes (DEGs) from GSE34303 dataset of human BMSCs were analyzed and used to perform Gene Ontology (GO) analysis between aged (Passage 10) and young (Passage 2) BMSCs. The results showed that with physiological aging, an increase was shown in the mRNA expression of SASP genes (*IL1A*, *IL1B*, *TNFRSF11B*, *TNFSF4*, *CD36*, *CCL2*, *CXCL8*, and *IL17D*) and extracellular matrix degradation gene *TIMP3*, however, a decrease was shown in the mRNA expression of extracellular matrix synthesis genes (*COL3A1*, *COL10A1*, *COL11A1*, *COL12A1*), stem cell regulatory proliferative and differential genes (*RUNX2, EZH2*, *GATA6*, *LMNB1*, *CD24*, *SOX11*, and *GLI3*), and cell proliferation and cell cycle regulation genes (*CCNB2*, *CCNB1*, *CDK1*, *E2F8*, and *MKI67*). Physiologically aged human BMSCs showed reduced expression of the anti‐aging marker *LMNB1*, the PRC complex component *EZH2*, and the osteogenic genes *RUNX2* (**Figure** **1**A). Meanwhile, with physiological aging, BMSCs exhibited changes in mesenchymal cell differentiation, mesenchyme development, cell proliferation and cell cycle regulation, DNA replication, bone development and formation, aging, BMP signaling pathway, bone mineralization and its regulation, bone morphogenesis, regulation of ossification, mesenchymal cell development, bone remodeling and its regulation, bone regeneration and its regulation, and bone resorption and its regulation (Figure 1B).

GSEA between young (Passage 2) and aged (Passage 10) human BMSCs showed that physiological aging led to the downregulation of proliferation signaling, including positive regulation of the cell cycle process and marker of proliferation Ki67 (MKI67); osteogenesis signaling, including osteoblast differentiation, RUNX2‐regulated osteoblast differentiation, and RUNX2‐mediated transcriptional regulation; and PRC2 methylation signaling, including PRC2‐EZH2‐UP and methylation of histones and DNA by PRC2; however, senescence and SASP‐NF‐κB signaling and adipogenesis signaling, including positive regulation of the lipid biosynthetic process, were upregulated (Figure S1, Supporting Information).

We further analyzed changes in mouse BMSCs with aging. As compared to young BMSCs, old BMSCs showed a decrease in the mRNA expression of *Bmi‐1*, *Ezh2*, *Runx2*, *Sp7*, *Bglap*, and *alp*, and in the protein expression of Bmi‐1, EZH2, H3K27me3, Runx2, Osterix, osteocalcin (OCN) and alkaline phosphatase (ALP). However, old BMSCs showed an increase in the mRNA expression of *Pparg*, *Dnmt3a*, *p16*, *p21*, and *Cebpa*, and in the protein expression of p16, p21, PPARγ, C/EBPα and DNMT3A (Figure 1C–G; Figure S2A–C, Supporting Information).

To confirm the potential role of Bmi‐1 in the osteogenic and adipogenic differentiation of BMSCs, young BMSCs were subjected to differentiation experiments. The results showed that during adipogenic differentiation, the mRNA expression of *Pparg* and *Cebpa* expression was upregulated, while *Bmi‐1* and *Ezh2* expression was downregulated (Figure 1H). Correspondingly, the protein expressions PPARγ and C/EBPα were upregulated, whereas the expressions of Bmi‐1 and EZH2 were downregulated (Figure S2D,E, Supporting Information). During osteogenic differentiation, the mRNA expression of *Bmi‐1*, *Runx2*, *Sp7*, *alp*, and *Bglap* was upregulated (Figure 1I). Similarly, the protein levels of Bmi‐1, Runx2, Osterix, ALP, and OCN were elevated (Figure S2F,G, Supporting Information). These results suggest that Bmi‐1 may epigenetically regulate the osteogenic and adipogenic differentiation of BMSCs during aging.

### 2.2 Prx1 and Bmi‐1 are Co‐Localized in Trabecular Bone, Bone Marrow Cavity, Endosteum, and Periosteum

To clarify the cellular co‐localization of Prx1 and Bmi‐1 in long bone, we constructed a *tdTOMATO^+^Prx1‐cre* mice model for tracking. The results showed that Bmi‐1 was expressed in trabecular bone, bone marrow cavity, endosteum, periosteum, articular cartilage, and growth plate, while Prx1 was expressed in trabecular bone, bone marrow cavity, endosteum, and periosteum in adult mice, but not in articular cartilage and growth plate. Both Prx1 and Bmi‐1 were co‐localized in trabecular bone, bone marrow cavity, endosteum, and periosteum (Figure 1J–L; Figure S3A,B, Supporting Information).

### 2.3 *Prx1*‐Driven *Bmi‐1* Knockout in BMSCs does not Affect Bone Size

To determine the role of Bmi‐1 in regulating the biological characteristics of BMSCs in vivo, we generated mice with conditional knockout of *Bmi‐1* in BMSCs (*Bmi‐1^f/f^Prx1‐cre*). Our results showed no significant difference in body size and long bone length between 6‐week‐old *Bmi‐1^f/f^Prx1‐cre* mice and *Bmi‐1^f/f^* littermates (Figure S3C–E, Supporting Information). *Bmi‐1* was knocked down in the primary and secondary bone trabecular bones (Figure S3F,G, Supporting Information). No difference in long bone size and growth plate width was observed between *Bmi‐1^f/f^Prx1‐cre* mice and *Bmi‐1^f/f^* littermates (Figure S3H–L, Supporting Information). Thus, *Prx1*‐driven *Bmi‐1* knockout in BMSCs did not affect bone size.

### 2.4 *Prx1*‐Driven *Bmi‐1* Knockout in BMSCs Reduces Bone Mass but Increases Bone Marrow Adiposity

**Figure 2:**
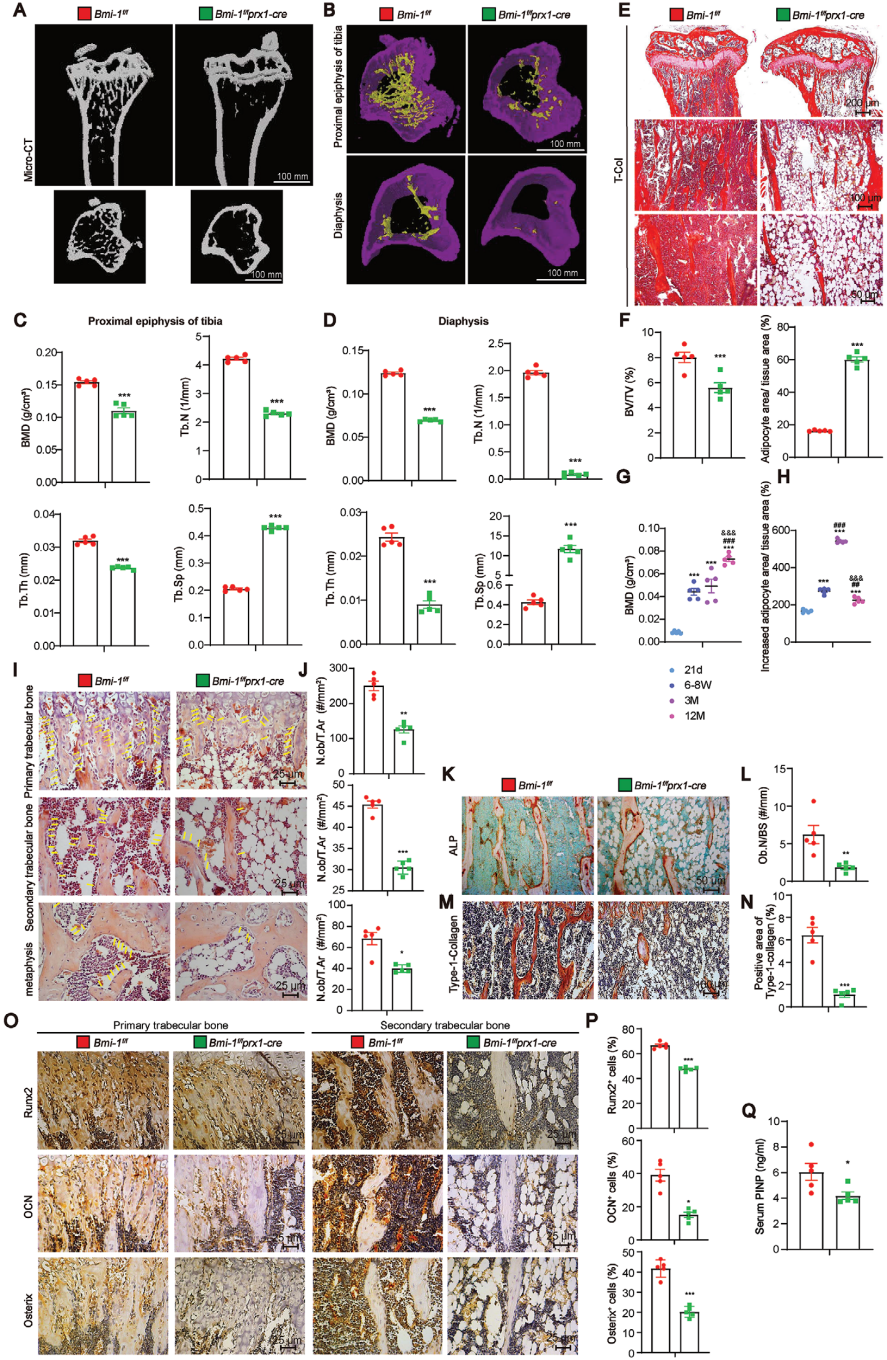
*Prx1*‐driven *Bmi‐1* knockout in BMSCs reduces bone mass and osteoblastic bone formation but increases bone marrow adiposity. A,B) Representative 3D reconstruction of the µCT scan of the proximal epiphysis or diaphysis in the tibia of 8‐week‐old *Bmi‐1^f/f^Prx1‐cre* mice and *Bmi‐1^f/f^* littermates. C,D) Quantitative analysis of bone formation parameters, including BMD, Tb.N, Tb.Th, and Tb.Sp, of proximal and distal epiphyses. E) Representative micrographs of total collagen staining. F) Quantitative analysis of BV/TV and percentage of the adipocyte area in the tibia. Five mice per group were used for the experiments. Values are expressed as mean ± SEM of five determinations per group. ^***^ *P* < 0.001, compared to *Bmi‐1^f/f^* littermates, unpaired Student's *t*‐test for bar graphs. G,H) The proportion of reduced bone mass and increased bone marrow in *Bmi‐1^f/f^Prx1‐cre* mice as compared to *Bmi‐1^f/f^* littermates at different ages, including 21 days, 6–8 weeks, 3 months, and 12 months. Five mice per group were used for the experiments. Statistical analysis was performed with one‐way ANOVA for pairwise comparisons. Values are expressed as mean ± SEM of five determinations per group, ^***^ *P* < 0.001, compared to 21 days old mice; ^##^ *P* < 0.01, ^###^ *P* < 0.001, compared to 6–8 weeks old mice; ^&&&^ *P* < 0.001, compared to 3 months old mice. I) Representative micrographs of the metaphysis and primary and secondary trabecular bone sections stained with hematoxylin and eosin. J) Quantitative analysis of the number of osteoblasts per tissue area (N.Ob/T.Ar, #/mm^2^). K) Representative micrographs of the primary and secondary trabecular bone sections subjected to histochemical staining for ALP. L) Quantitative analysis of the number of osteoblasts/unit trabecular perimeter (Ob.N/BS). M) Representative micrographs of type‐1‐collagen immunostaining in tibial sections. N) Quantification of the percentages of type‐1‐collagen‐positive area. O) Representative micrographs of the primary and secondary trabecular bone sections immunostained for Runx2, OCN, and Osterix. P) Quantification of the percentages of Runx2‐, OCN‐, and Osterix‐positive osteoblasts. Q) Determination of serum propeptide of type I procollagen (PINP) levels (ng/mL). Five mice per group were used for the experiments. ^*^ *P* < 0.05, ^**^ *P* < 0.01, ^***^ *P* < 0.001, compared to *Bmi‐1^f/f^* mice, unpaired Student's *t*‐test for bar graphs.

To investigate whether *Prx1*‐driven *Bmi‐1* knockout in BMSCs affects bone mass, micro‐computed tomography (µCT) assay and total collagen (T‐Col) staining were performed. The results showed that compared to 8‐week‐old *Bmi‐1^f/f^* littermates, 8‐week‐old *Bmi‐1^f/f^Prx1‐cre* mice showed a significant decrease in bone mineral density (BMD), trabecular number (Tb.N), and trabecular thickness (Tb.Th) at the proximal epiphysis or diaphysis in the tibia, but a significant increase in trabecular separation (Tb.Sp) (**Figure** **2**A–D). The results of T‐Col staining were consistent with those of the µCT assay. Although 8‐week‐old *Bmi‐1^f/f^Prx1‐cre* mice showed a decrease in the ratio of bone volume over tissue volume (BV/TV), their bone marrow adiposity was apparently increased as compared to that in *Bmi‐1^f/f^* littermates (Figure 2E,F). Consistent with the results observed in the tibia, bone mass was reduced but bone marrow adiposity was increased in the femur of 8‐week‐old *Bmi‐1^f/f^Prx1‐cre* mice (Figure S4A–I, Supporting Information).

To clarify the effect of *Prx1*‐driven *Bmi‐1* knockout in BMSCs on bone mass and bone marrow adiposity in mice at the successive stages of bone development and growth until aging, we measured bone mass and bone marrow adiposity of 21‐day‐old, 6–8 weeks old, 3‐month‐old, and 12‐month‐old mice. The results showed that at every age, *Prx1*‐driven *Bmi‐1* knockout in BMSCs reduced bone mass and increased bone marrow adiposity. Moreover, the proportion of reduced bone mass gradually increased with age, and the proportion of increased bone marrow adiposity reached its peak at the age of 3 months in *Bmi‐1^f/f^Prx1‐cre* mice as compared to that in *Bmi‐1^f/f^* littermates (Figure 2G,H; Figures S4–S7, Supporting Information).

### 2.5 *Prx1*‐Driven *Bmi‐1* Knockout in BMSCs Reduces Osteoblastic Bone Formation but Increases Osteoclastic Bone Resorption

**Figure 3:**
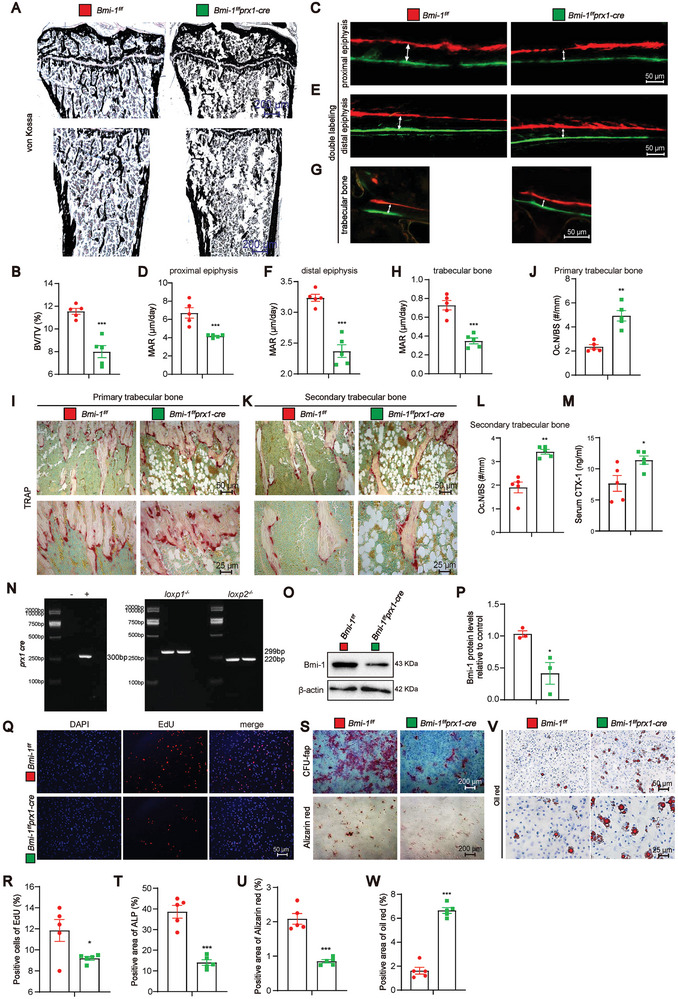
*Prx1*‐driven *Bmi‐1* knockout in BMSCs reduces mineralization capacity, bone formation rate, cell proliferation and osteogenesis differentiation, but increases osteoclastic bone resorption and adipogenic differentiation of BMSCs. A) Von Kossa staining of the tibia from 8‐week‐old *Bmi‐1^f/f^Prx1‐cre* mice and *Bmi‐1^f/f^* littermates. B) Quantitative analysis of BV/TV (%). C–H) Representative micrographs of Calcein/XO dual‐labeling and MAR (µm/day) analysis in proximal epiphysis, distal epiphysis, and trabecular bone respectively. I–L) Representative micrographs of primary and secondary trabecular bone sections subjected to histochemical staining for tartrate‐resistant acid phosphatase (TRAP), and quantitative analysis of the number of osteoclasts/unit trabecular perimeter (N.Oc/BS, #/mm). M) Serum c‐telopeptide of type‐1‐collagen (CTX‐1) levels (ng mL^−1^). Five mice per group were used for the experiments. ^*^ *P* < 0.05, ^**^ *P* < 0.01, ^***^ *P* < 0.001 compared to the *Bmi‐1^f/f^* mice, and unpaired Student's *t*‐test for bar graphs. N) Genotyping of BMSCs from *Bmi‐1^f/f^* and *Bmi‐1^f/f^prx1‐cre* mice. O) Western blotting assay of BMSC extracts showing the Bmi‐1 protein level. β‐Actin was used as the loading control. P) Protein expression relative to β‐actin was assessed by densitometric analysis. Three biological replicates per group were used for the experiments. ^*^ *P* < 0.05, compared to *Bmi‐1^f/f^* mice, unpaired Student's *t*‐test for bar graphs. Q) Representative micrographs of 5‐ethynyl‐2‐deoxyuridine (EdU) staining, with nuclei staining by DAPI. R) The percentage of EdU‐positive cells. S) Representative micrographs of CFU‐fap and Alizarin Red staining. T) The percentage of ALP‐positive cell area. U) The percentage of Alizarin Red‐positive cell area. V) Representative micrographs of Oil Red staining. W) The percentage of Oil Red‐positive cell area. Cell experiments were performed with five biological replicates per group. Values are expressed as mean ± SEM from five determinations per group. ^*^ *P* < 0.05, ^***^ *P* < 0.001, compared to the *Bmi‐1^f/f^* group, unpaired Student's *t*‐test.

To determine the reasons why *Prx1*‐driven *Bmi‐1* knockout in BMSCs reduces bone mass, we analyzed osteoblastic bone formation of 8‐week‐old mice. The results showed that compared to *Bmi‐1^f/f^* mice, *Bmi‐1^f/f^Prx1‐cre* mice showed a decrease in the number of osteoblasts in the tibia, including in primary trabecular bone, secondary trabecular bone, and metaphysis (Figure 2I,J). *Bmi‐1^f/f^Prx1‐cre* mice also exhibited a decrease in ALP‐positive area (Figure 2K,L) and Type‐1‐collagen‐positive area (Figure 2M,N). The number of Runx2‐, OCN‐, and Osterix‐positive cells also decreased (Figure 2O,P). The serum level of PINP, which represents the degree of bone formation,^[^ ^30^ ^]^ was decreased in *Bmi‐1^f/f^Prx1‐cre* mice as compared to that in *Bmi‐1^f/f^* mice (Figure 2Q). After frozen sectioning and von Kossa staining, the results showed that compared to 8‐week‐old *Bmi‐1^f/f^* mice, *Bmi‐1^f/f^Prx1‐cre* mice exhibited a decrease in BV/TV in the tibia, thus indicating that the specific knockout of *Bmi‐1* in BMSCs decreases mineralization capacity (**Figure** **3**A,B). Calcium green and xylenol orange (XO) were injected to observe the distance between them, which indicates new bone formation. The results showed that the bone mineralization apposition rate (MAR) was decreased in the proximal and distal epiphyses and trabecular bone (Figure 3C–H). These findings suggested that *Prx1*‐driven *Bmi‐1* knockout in BMSCs reduced osteoblastic bone formation.

To determine the reasons why *Prx1*‐driven *Bmi‐1* knockout in BMSCs reduced bone mass, we analyzed osteoclastic bone resorption in 8‐week‐old mice by using TRAP histochemical staining. The results showed that compared to *Bmi‐1^f/f^* mice, *Bmi‐1^f/f^Prx1‐cre* mice exhibited a significant increase in TRAP‐positive osteoclast surface in the primary and secondary trabecular bones (Figure 3I–L). The CTX‐1 is a marker of the degree of bone resorption.^[^ ^30^ ^]^ *Bmi‐1^f/f^Prx1‐cre* mice showed a significant increase in the serum CTX‐1 level as compared to *Bmi‐1^f/f^* mice (Figure 3M). These results suggest that *Prx1*‐driven *Bmi‐1* knockout in BMSCs increased osteoclastic bone resorption.

### 2.6 *Prx1*‐Driven *Bmi‐1* Knockout in BMSCs Reduces Proliferation and Osteogenic Differentiation but Increases Adipogenic Differentiation of BMSCs In Vitro

To observe whether *Bmi‐1* knockout reduced osteoblastic bone formation and promoted bone marrow adiposity in vitro, BMSCs were cultured and genotyped (Figure 3N), and the knockout efficiency was then confirmed (Figure 3O,P). The results showed that the EdU‐positive area was reduced in *Bmi‐1^f/f^Prx1‐cre* mice as compared to that in *Bmi‐1^f/f^* mice (Figure 3Q,R). Moreover, the CFU‐fap area and Alizarin Red‐positive area were significantly reduced in *Bmi‐1^f/f^Prx1‐cre* mice (Figure 3S–U). However, the Oil Red‐positive area was significantly increased in *Bmi‐1^f/f^Prx1‐cre* mice as compared to that in *Bmi‐1^f/f^* mice (Figure 3V,W). These results suggest that *Prx1*‐driven *Bmi‐1* promoted proliferation and osteogenic differentiation but decreased adipogenic differentiation of BMSCs.

### 2.7 *Prx1*‐Driven *Bmi‐1* Overexpression in BMSCs Increases Bone Mass by Promoting Osteoblastic Bone Formation but Inhibiting Osteoclastic Bone Resorption

**Figure 4:**
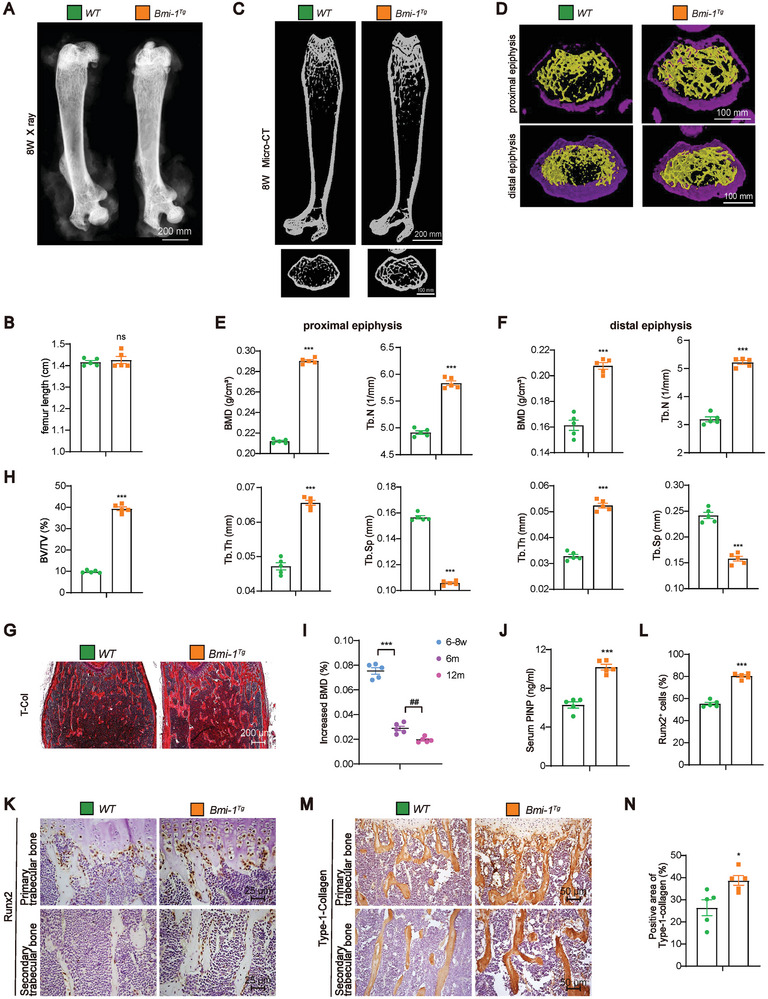
*Prx1*‐driven *Bmi‐1* overexpression in BMSCs increases bone mass by promoting osteoblastic bone formation. A) Representative micrographs of X‐ray for femurs. B) Femur length (cm). C,D) Representative 3D reconstruction of the µCT scan of the proximal epiphysis or diaphysis in the femur of 8‐week‐old WT and *Bmi‐1^Tg^* mice littermates. E,F) Quantitative analysis of bone formation parameters, including BMD, Tb.N, Tb.Th, and Tb.Sp, of proximal and distal epiphyses in the femur. G) Representative micrographs of T‐Col staining. H) Quantitative analysis of BV/TV (%). Five mice per group were used for the experiments. Values are expressed as mean ± SEM from five determinations per group. ^***^ *P* < 0.001, compared to WT mice, unpaired Student's *t*‐test. I) The proportion of increased BMD in *Bmi‐1^Tg^* mice compared to that in WT littermates at different ages, including 6–8 weeks, 6 months, and 12 months. Five mice per group were used for the experiments. Statistical analysis was performed with one‐way ANOVA for pairwise comparisons. Values are expressed as mean ± SEM of five determinations per group. ^***^ *P* < 0.001, compared to 6–8 weeks old mice; ^##^ *P* < 0.01, compared to 6 months old mice. J) Determination of serum PINP levels (ng mL^−1^). K) Representative micrographs of primary and secondary trabecular bone sections immunostained for Runx2. L) Quantification of the percentage of Runx2‐positive osteoblasts. M) Representative micrographs of primary and secondary trabecular bone sections immunostained for Type‐1‐collagen. N) Quantification of the percentage of Type‐1‐collagen‐positive area. Five mice per group were used for the experiments, Values are expressed as mean ± SEM from five determinations per group. ^*^ *P* < 0.05, ^***^ *P* < 0.001, compared to WT mice, unpaired Student's *t*‐test.

To determine whether *Prx1*‐driven *Bmi‐1* overexpression in BMSCs affects bone mass, we generated *Bmi‐1^Tg^* mice with *Bmi‐1* overexpression in BMSCs (Figure S8A,B, Supporting Information) and performed µCT assay and T‐Col staining. *Bmi‐1* overexpression significantly increased bone mass parameters, including BMD, Tb.Th, Tb.N, and BV/TV, and decreased Tb.Sp in both proximal and distal epiphyses (**Figure** **4**A,C–H). However, compared to WT littermates, *Bmi‐1* overexpression did not change the body length, weight, femur and tibia length and width of the growth plate in *Bmi‐1^Tg^* mice (Figure 4B; Figure S8, Supporting Information).

To clarify the effect of *Prx1*‐driven *Bmi‐1* overexpression in BMSCs on bone mass in mice at the successive stages of bone growth until aging, we observed bone mass of 2‐month‐old, 7‐month‐old, and 12‐month‐old mice. The results showed that at every age group, *Prx1*‐driven *Bmi‐1* overexpression in BMSCs increased bone mass. Moreover, the proportion of increased BMD gradually reduced in *Bmi‐1^Tg^* mice with aging as compared to that in *Bmi‐1^f/f^* littermates (Figure 4C–I; Figures S9 and S10, Supporting Information).

**Figure 5:**
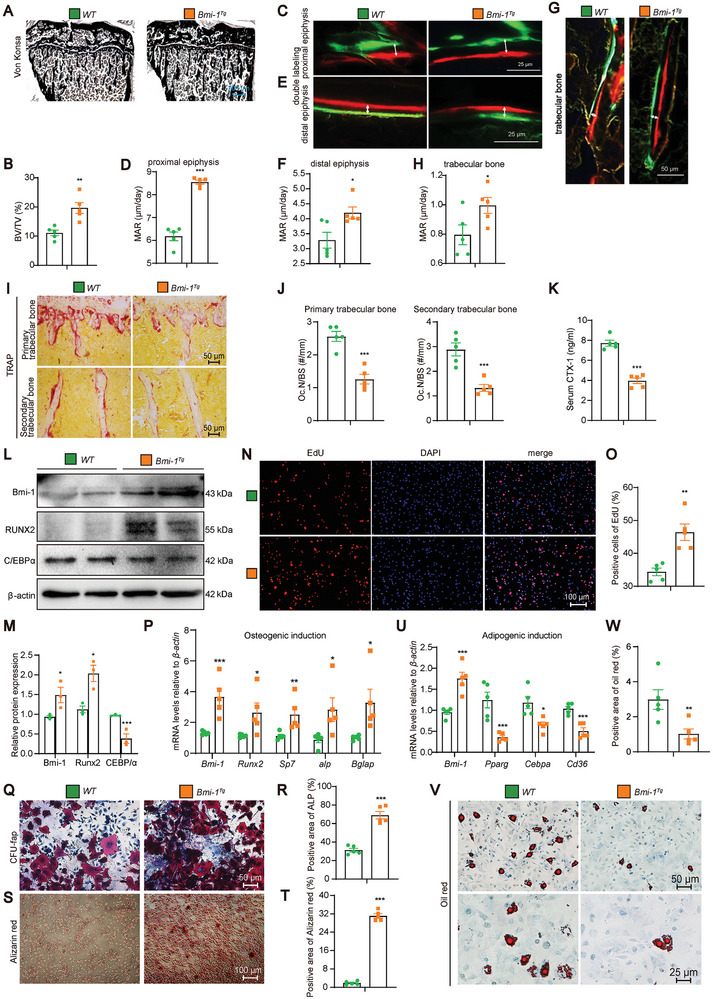
*Prx1*‐driven *Bmi‐1* overexpression in BMSCs increases proliferation and osteogenic differentiation, but reduces osteoclastic bone resorption and adipogenic differentiation of BMSCs. A) Von Kossa staining of the tibia from 8‐week‐old *WT* and *Bmi‐1^Tg^* mice littermates. B) Quantitative analysis of BV/TV (%). C–H) Representative micrographs of Calcein/ XO dual‐labeling and MAR (µm per day) analysis. I) Representative micrographs of primary and secondary trabecular bone sections subjected to histochemical staining for TRAP. J) Quantitative analysis of the number of Oc.N/BS. K) Serum CTX‐1 levels (ng mL^−1^). Five mice per group were used for the experiments. Values are expressed as mean ± SEM from five determinations per group. ^*^ *P* < 0.05, ^**^ *P* < 0.01, ^***^ *P* < 0.001, compared to WT mice, unpaired Student's *t*‐test. L) Western blotting assay of BMSC extracts showing the protein levels of Bmi‐1, Runx2, and C/EBPα. β‐Actin was used as the loading control. M) Protein expression relative to β‐actin was assessed by densitometric analysis. Three mice per group were used for the experiments. Values are expressed as mean ± SEM from three determinations per group. ^*^ *P* < 0.05, ^***^ *P* < 0.001, compared to WT mice, unpaired Student's *t*‐test. N) Representative micrographs of EdU staining, with nuclei staining by DAPI. O) The percentage of EdU‐positive cells. P) BMSCs were induced for osteogenic differentiation. The mRNA levels of *Bmi‐1, Runx2, Sp7, alp*, and *Bglap* in BMSCs detected by RT‐qPCR and calculated and expressed relative to control *β‐actin* mRNA. Q) Representative micrographs of CFU‐fap staining. R) The percentage of ALP‐positive cell area. S) Representative micrographs of Alizarin Red staining. T) The percentage of Alizarin Red‐positive cell area. U) The mRNA levels of *Bmi‐1, Pparg, Cebpa*, and *Cd36* in BMSCs detected by RT‐qPCR and calculated and expressed relative to control *β‐actin* mRNA. V) BMSCs were induced for adipogenic differentiation. Representative micrographs of Oil Red staining. W) The percentage of Oil Red‐positive cell area. Cell experiments were performed with five biological replicates per group. Values are expressed as mean ± SEM from five determinations per group. ^*^ *P* < 0.05, ^**^ *P* < 0.01, ^***^ *P* < 0.001, compared to the WT group, unpaired Student's *t*‐test.

To determine the reasons why *Prx1*‐driven *Bmi‐1* overexpression in BMSCs increases bone mass, we analyzed osteoblastic bone formation and osteoclastic bone resorption in 8‐week‐old mice. The results showed that compared to WT littermates, *Bmi‐1^Tg^* mice exhibited an increase in the serum level of PINP, Type‐1‐collagen‐positive area, and the number of Runx2‐ and OCN‐positive cells, BV/TV, MAR (Figures 4J–N and 5A–H; Figure S8K,L, Supporting Information); however, the number of osteoclasts and the serum level of CTX‐1 decreased in *Bmi‐1^Tg^* mice (**Figure** **5**I–K). These results indicated that *Bmi‐1* overexpression in BMSCs could increase bone mass by promoting osteoblastic bone formation and inhibiting osteoclastic bone resorption.

### 2.8 *Prx1*‐Driven *Bmi‐1* Overexpression in BMSCs Increases Proliferation and Osteogenic Differentiation but Reduces Adipogenic Differentiation of BMSCs

BMSCs were isolated from WT and *Bmi‐1^Tg^* mice to observe whether *Bmi‐1* overexpression increases proliferation and osteogenic differentiation but reduces adipogenic differentiation of BMSCs. The results showed that compared to WT mice, *Bmi‐1^Tg^* mice exhibited an increase in the protein and mRNA expression levels of Bmi‐1 and Runx2; however, this mice group showed a decrease in the protein expression level of C/EBPα in BMSCs (Figure 5L,M). The EdU‐positive area also increased in *Bmi‐1^Tg^* mice (Figure 5N,O). Under osteogenic induction, *Bmi‐1^Tg^* mice showed an increase in the mRNA levels of osteogenesis‐related genes *Runx2*, *Sp7*, *alp*, and *Bglap* and CFU‐fap and Alizarin Red‐positive areas as compared to WT mice (Figure 5P–T). Under adipogenic induction, the mRNA levels of the adipogenesis‐related genes *Pparg*, *Cebpa*, and *Cd36* and the Oil Red‐positive area were decreased in *Bmi‐1^Tg^* mice as compared to those in WT mice (Figure 5U,W). These results further confirmed that *Prx1*‐driven *Bmi‐1* promoted proliferation and osteogenic differentiation but inhibited adipogenic differentiation of BMSCs.

### 2.9 Bmi‐1‐RING1B Binds to DNMT3A to Promote its Ubiquitination and Inhibits *Runx2* DNA Methylation, thus Promoting the Osteogenic Differentiation of BMSCs

**Figure 6:**
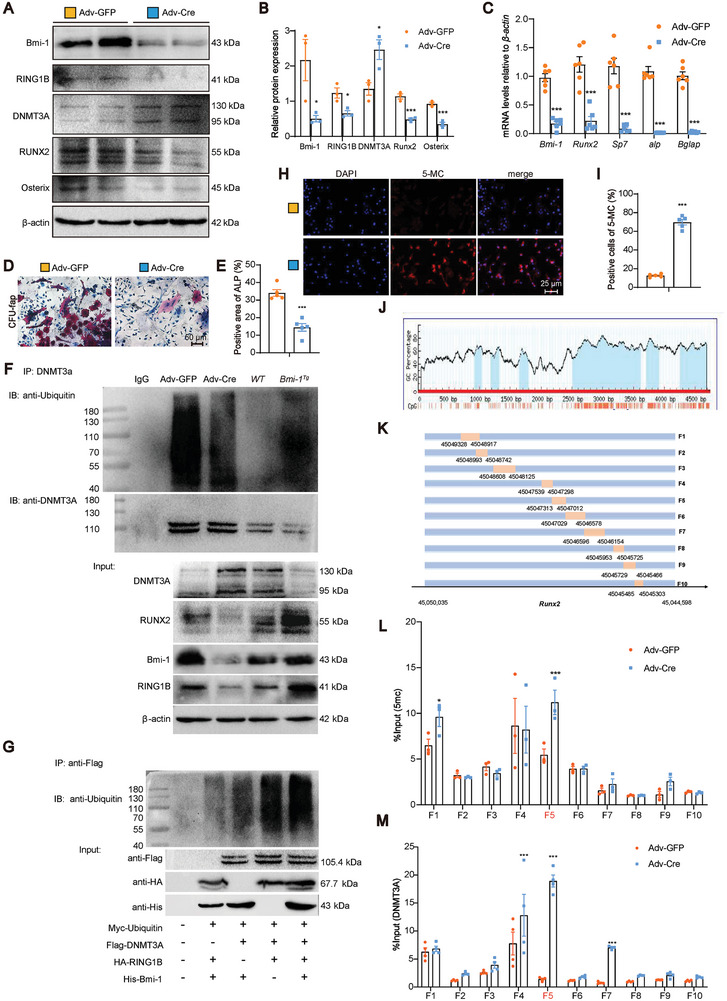
Bmi‐1‐RING1B binds to DNMT3A to promote its ubiquitination and inhibits *Runx2* DNA methylation, thereby inducing the osteogenic differentiation of BMSCs. Primary BMSCs extracted from *Bmi‐1^f/f^* mice were transfected with Adv‐GFP or Adv‐Cre adenovirus to construct primary BMSCs with *Bmi‐1* knockdown group (Adv‐Cre) or vehicle group (Adv‐GFP). A) Western blotting assay of BMSC extracts showing the protein levels of Bmi‐1, RING1B, DNMT3A, RUNX2, and Osterix. β‐Actin was used as the loading control. B) Protein expression relative to β‐actin was assessed by densitometric analysis. C) BMSCs were induced for osteogenic differentiation. The mRNA levels of *Bmi‐1, Runx2, Sp7, alp*, and *Bglap* in BMSCs detected by RT‐qPCR and calculated and expressed relative to control *β‐actin* mRNA. D) Representative micrographs of CFU‐fap staining. E) The percentage of the ALP‐positive cell area. Cell experiments were performed with five biological replicates per group. Values are expressed as mean ± SEM from five determinations per group. ^*^ *P* < 0.05, ^***^ *P* < 0.001, compared to the Adv‐GFP group, unpaired Student's *t*‐test. F) BMSCs from the Adv‐GFP group, Adv‐Cre group, WT, and *Bmi‐1^Tg^* mice were isolated and subjected to immunoprecipitation with anti‐DNMT3A antibodies. Western blotting assays were performed for detecting ubiquitin and DNMT3A in immunoprecipitation samples and for detecting DNMT3A, RUNX2, Bmi‐1, and RING1B in input samples. G) HEK293T cells were transfected with full‐length plasmids, including Myc‐Ubiquitin, Flag‐DNMT3A, HA‐RING1B, and/or His‐Bmi‐1. The cells were subjected to immunoprecipitation with anti‐Flag‐tag antibodies and then analyzed by Western blotting assay for detecting ubiquitin. The input proteins were detected by Western blotting assay using anti‐Flag‐tag, anti‐HA‐tag, or anti‐His‐tag antibodies. H) Representative micrographs of 5‐methylcytosine (5‐MC) immunostaining, with nuclei staining by DAPI. I) The percentage of 5‐MC‐positive cells. Cell experiments were performed with five biological replicates per group. Values are expressed as mean ± SEM from five determinations per group. ^***^ *P* < 0.001, compared to the Adv‐GFP group, unpaired Student's *t*‐test. J) CpG enrichment in *Runx2* was predicted by MethPrimer 2.0. K) An illustration of *Runx2* truncated primers. L,M) Chromatin immunoprecipitation (ChIP) assays were performed with chromatin prepared from BMSCs. The chromatin was immunoprecipitated with normal rabbit IgG or anti‐5‐MC and anti‐DNMT3A antibodies, and the precipitated genomic DNA was analyzed as relative enrichment by qPCR using different primers for the different regions of *Runx2*. Cell experiments were performed with three biological replicates per group. Values are expressed as mean ± SEM from three determinations per group. ^***^ *P* < 0.001, compared to the Adv‐GFP group, unpaired Student's *t*‐test.

To investigate whether Bmi‐1 epigenetically regulates osteogenic differentiation, we isolated primary BMSCs from *Bmi‐1^f/f^* mice and transfected them with Adv‐Cre or Adv‐GFP adenovirus. The results showed that in the Adv‐Cre‐treated group, the protein levels of Bmi‐1, RING1B, Runx2, and Osterix were reduced; however, the DNMT3A protein level was increased as compared to that in the Adv‐GFP‐treated group (**Figure** **6**A,B). In accordance with the abovementioned changes in osteogenic transcription factors, the mRNA levels of osteogenesis‐related genes *Runx2*, *Sp7*, *alp*, and *Bglap* and the CFU‐fap‐positive area were decreased in the Adv‐Cre‐treated group (Figure 6C–E). These results showed that Bmi‐1 promoted the osteogenic differentiation of BMSCs.

We recently reported that Bmi‐1 significantly enhanced the E3 ubiquitin ligase activity of RING1B to promote protein ubiquitination in addition to the modification of histone ubiquitination.^[^ ^19^, ^20^ ^]^ Bioinformatics analysis (https://www.uniprot.org/align/) was conducted, and the results showed that DNMT3A shared the same conserved region and amino acid residues in the active domain with GATA4. In the present study, to determine whether Bmi‐1‐RING1B could promote the ubiquitination degradation of DNMT3A to inhibit *Runx2* DNA methylation, we used BMSCs with *Bmi‐1* knockdown or overexpression and performed protein immunoprecipitation assay. Anti‐DNMT3A antibodies were used to capture proteins that were bound to bait proteins. Anti‐ubiquitin antibodies were used to detect the ubiquitination levels of DNMT3A. The results showed that the DNMT3A ubiquitination levels in BMSCs with *Bmi‐1* knockdown were significantly lower than those in vehicle BMSCs; however, the DNMT3A ubiquitination levels were significantly higher in *Bmi‐1*‐overexpressed BMSCs than in WT BMSCs (Figure 6F). In the ubiquitination assay, Bmi‐1 and RING1B co‐overexpression apparently increased the ubiquitination level of DNMT3A (Figure 6G). These results suggest that the formation of the PRC1 protein complex by Bmi‐1‐RING1B promoted the ubiquitination of DNMT3A.

To further clarify whether DNMT3A can promote the DNA methylation of *Runx2*, we conducted 5‐MC immunofluorescence staining and Methyl Primer Express Software analysis. We detected 5‐MC expression and found that the number of 5‐MC‐positive cells was apparently increased in the Adv‐Cre‐treated group (Figure 6H,I). Methyl Primer Express Software analysis revealed the presence of CpG islands in *Runx2* (Figure 6J). Next, to determine whether DNMT3A can bind to *Runx2*, a series of mouse *Runx2* primers were designed according to the above‐mentioned CpG islands (Figure 6K). ChIP assay was performed, and anti‐5‐MC and anti‐DNMT3A primary antibodies were used to capture chromatin. The results showed that DNMT3A and 5‐MC exhibited significant binding to *Runx2* at the region from 45047012 to 45047313 bp as compared to the control group (Figure 6L,M). These results demonstrated that Bmi‐1‐RING1B bound to DNMT3A to promote its ubiquitination and inhibited the DNA methylation of *Runx2*, thus promoting the osteogenic differentiation of BMSCs.

### 2.10 Bmi‐1‐EZH2 Represses the Transcription of *Cebpa* by Promoting H3K27 trimethylation at the Promoter Region, thus Inhibiting the Adipogenic Differentiation of BMSCs

**Figure 7:**
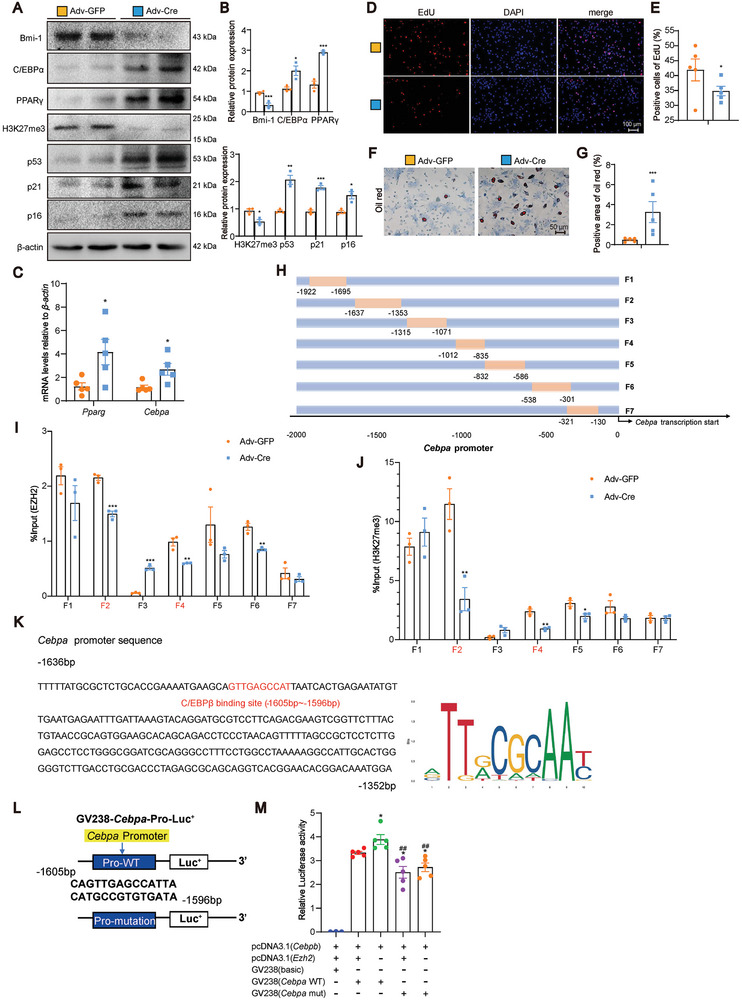
Bmi‐1‐EZH2 represses the transcription of *Cebpa* by promoting H3K27 trimethylation at the promoter region, thus inhibiting the adipogenic differentiation of BMSCs. A) Western blotting assay of BMSC extracts showing the protein levels of Bmi‐1, C/EBPα, PPARγ, H3K27me3, p53, p21, and p16. β‐actin was used as the loading control. B) Protein expression relative to β‐actin was assessed by densitometric analysis. Cell experiments were performed with three biological replicates per group. Values are expressed as mean ± SEM from three determinations per group. ^*^ *P* < 0.05, ^**^ *P* < 0.01, ^***^ *P* < 0.001, compared to the Adv‐GFP group, unpaired Student's *t*‐test. C) BMSCs were induced for adipogenic differentiation. The mRNA levels of *Pparg* and *Cebpa* in BMSCs were detected by RT‐qPCR and calculated and expressed relative to control *β‐actin* mRNA. D) Representative micrographs of EdU staining, with nuclei staining by DAPI. E) The percentage of EdU‐positive cells. F) BMSCs were induced for adipogenic differentiation. Representative micrographs of Oil Red staining. G) The percentage of Oil Red‐positive cell area. Cell experiments were performed with five biological replicates per group. Values are expressed as mean ± SEM from five determinations per group. ^*^ *P* < 0.05, ^***^ *P* < 0.001, compared to the Adv‐GFP group, unpaired Student's *t*‐test. H) Illustration of *Cebpa* promoter truncated primers. I,J) ChIP assays were performed with the chromatin prepared from BMSCs. The chromatin was immunoprecipitated with normal rabbit IgG or anti‐EZH2 and anti‐H3K27me3 antibodies, and the precipitated genomic DNA was analyzed as relative enrichment by qPCR using different primers for the different regions of the *Cebpa* promoter. Cell experiments were performed with three biological replicates per group. Values are expressed as mean ± SEM from five or three determinations per group. ^*^ *P* < 0.05, ^**^ *P* < 0.01, ^***^ *P* < 0.001, compared to the Adv‐GFP group, unpaired Student's *t*‐test. K) The JASPAR database (https://jaspar.elixir.no) was utilized for motif sequence analysis. C/EBPβ‐like sequence binding site (highlighted in yellow) in the *Cebpa* promoter region, and C/EBPβ‐like sequence highlighted in red. L) Schematic diagram of the GV238‐*Cebpa* promoter reporter plasmid and mutant constructs of the GV238‐*Cebpa* promoter reporter plasmid. M) *Cebpa* promoter activity was measured by a luciferase reporter gene assay. Five biological replicates were used for each experiment. Statistical analysis was performed with one‐way ANOVA. Cell experiments were performed with three biological replicates per group. Values are expressed as mean ± SEM from five determinations per group. ^*^ *P* < 0.05, compared to the *Cebpb*, *Ezh2*, and *Cebpa‐*WT co‐transfected group; ^##^ *P* < 0.01, compared to the *Cebpb* and *Cebpa*‐WT co‐transfected group.

To determine whether Bmi‐1 epigenetically regulates adipogenic differentiation, we isolated primary BMSCs from *Bmi‐1^f/f^* mice and transfected them with Adv‐Cre or Adv‐GFP. The results showed that the protein levels of Bmi‐1 and H3K27me3 were reduced in the Adv‐Cre‐treated group; however, the protein levels of C/EBPα, PPARγ, p53, p21, and p16 were increased as compared to those in the Adv‐GFP‐treated group (**Figure** **7**A,B). Consistent with the increase in the above‐mentioned aging markers and adipogenesis transcriptional factors, the number of EdU‐positive cells was decreased and the Oil Red‐positive area was increased in the Adv‐Cre‐treated group (Figure 7C–F). These results revealed that Bmi‐1 prevented adipogenic differentiation and aging of BMSCs.

Because the mRNA levels of *Pparg* and *Cebpa* were increased in the Adv‐Cre‐treated group (Figure 7G), we investigated whether Bmi‐1‐EZH2 inhibits the transcription of the genes *Pparg* and *Cebpa* through H3K27 trimethylation. We constructed a series of primers that targeted mouse *Pparg* and *Cebpa* promoters (Figure 7H; Figure S11A, Supporting Information) and detected the binding of H3K27me3 and EZH2 to *Pparg* and *Cebpa* after *Bmi‐1* was knocked down in BMSCs by ChIP. The results of the ChIP assay showed that EZH2 and H3K27me3 exhibited significant binding to the *Cebpa* promoter at the regions F2 (−1637 to −1353) and F4 (−1012 to −835) (Figure 7I,J) and to the *Pparg* promoter at the regions F1 (−1995 to −1536), F2 (−1570 to −1410), F5 (−577 to −346), and F6 (−355 to −83) as compared to the control group (Figure S11B,C, Supporting Information). These results demonstrated that EZH2 could bind to the *Pparg* and *Cebpa* promoters at the regions indicated and induced H3K27 trimethylation, which eventually inhibited the mRNA expression of *Pparg* and *Cebpa*.

To further confirm whether *Cebpa* expression was regulated by Bmi‐1‐EZH2 at the transcriptional level, *Cebpb*‐ and *Ezh2*‐overexpressing plasmids were transfected into C3H10T1/2 cells. A C/EBPβ‐like sequence was identified at −1605 to −1596 bp upstream of the transcriptional start site of the *Cebpa* gene (JASPAR CORE database; http://jaspar.genereg.net/) (Figure 7K). Therefore, mouse *Cebpa* promoter‐carrying luciferase reporter plasmids with a C/EBPβ‐like sequence or a C/EBPβ‐like sequence (−1605 to −1596 bp) with mutation were constructed (Figure 7L). The results of the luciferase assay showed that the luciferase activity was decreased in C3H10T1/2 cells transfected with a *Ezh2*‐overexpressing plasmid as compared to that in the vehicle group. In contrast, the luciferase activity was decreased in C3H10T1/2 cells transfected with a GV238‐*Cebpa* mutation plasmid as compared to that in cells transfected with a C/EBPβ‐like sequence plasmid; thus, EZH2 could not activate the mutant reporter (Figure 7M). These results suggest that Bmi‐1‐EZH2 regulated *Cebpa* expression by regulating H3K27 trimethylation at the transcriptional level.

### 2.11 *Prx1*‐Driven *Bmi‐1* Overexpression in BMSCs Rescues the SOP Phenotype Induced by *Prx1*‐Driven *Bmi‐1* Knockout in BMSCs

**Figure 8:**
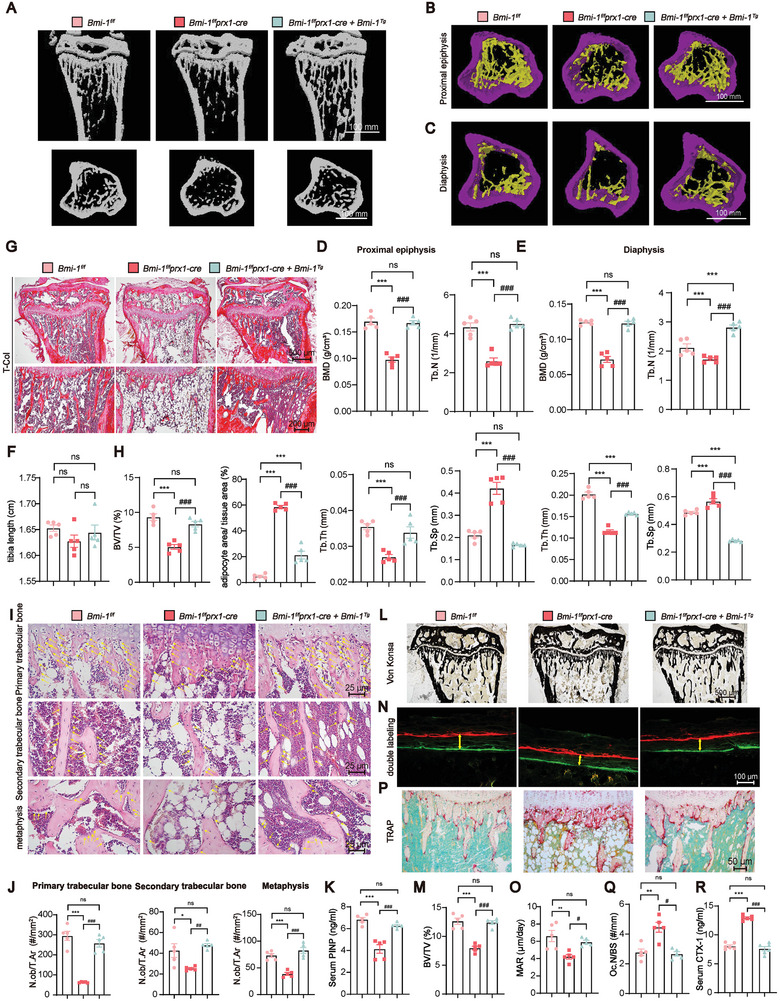
*Prx1*‐driven *Bmi‐1* overexpression in BMSCs rescues the SOP phenotype induced by *Prx1*‐driven *Bmi‐1* knockout in BMSCs in vivo*. Bmi‐1^f/f^Prx1‐cre* with *Bmi‐1^Tg^* (*Bmi‐1^f/f^Prx1‐cre & Bmi‐1^Tg^*) mice were generated, and the bone phenotype was compared with that of *Bmi‐1^f/f^* or *Bmi‐1^f/f^ Prx1‐cre* mice. A–C) Representative 3D reconstruction of the µCT scan of the proximal epiphysis or diaphysis in the tibia. D,E) Quantitative analysis of bone formation parameters, including BMD, Tb.N, Tb.Th, and Tb.Sp, of proximal epiphysis and diaphysis in the tibia. F) Quantification of tibial length (cm). G) Representative micrographs of T‐Col staining. H) Quantitative analysis of BV/TV and the percentage of the adipocyte area in the proximal tibia. I) Representative micrographs of metaphysis and primary and secondary trabecular bone sections stained with hematoxylin and eosin. J) Quantitative analysis of the number of N.Ob/T.Ar. K) Serum PINP levels (ng ml^−1^). L) Von Kossa staining of the tibia from the indicated groups of mice. M) Quantitative analysis of BV/TV (%). N,O) Representative micrographs of Calcein/XO dual‐labeling and MAR (µm per day) analysis. P) Representative micrographs of primary and secondary trabecular bone sections subjected to histochemical staining for TRAP. Q) Quantitative analysis of the N.Oc/BS. R) Serum CTX‐1 levels (ng mL^−1^). Five mice per group were used for the experiments. Statistical analysis was performed with one‐way ANOVA. Values are expressed as mean ± SEM from five determinations per group. ^*^ *P* < 0.05, ^**^ *P* < 0.01, ^***^ *P* < 0.001, compared to *Bmi‐1^f/f^* mice. ^#^ *P* < 0.05, ^##^ *P* < 0.01, ^###^ *P* < 0.001, compared to *Bmi‐1^f/f^Prx1‐cre* mice.

To determine whether *Prx1*‐driven *Bmi‐1* overexpression in BMSCs could correct the SOP phenotype in mice with *Prx1*‐driven *Bmi‐1* knockout, *Bmi‐1^f/f^Prx1‐cre* with *Bmi‐1^Tg^* (*Bmi‐1^f/f^Prx1‐cre & Bmi‐1^Tg^*) mice were generated, and the bone phenotype was compared with that of *Bmi‐1^f/f^* or *Bmi‐1^f/f^ Prx1‐cre* mice. We compared the differences in bone volume‐related parameters between the two genotypes of mice by µCT scanning, 3D reconstruction, and T‐Col staining. The results showed that compared to *Bmi‐1^f/f^Prx1‐cre* mice, 8‐week‐old *Bmi‐1^f/f^Prx1‐cre & Bmi‐1^Tg^* mice exhibited a significant increase in bone mass parameters at the proximal epiphysis and diaphysis of the tibia and femur, including BMD, Tb.N, Tb.Th, T‐Col‐positive area, the number of osteoblasts, the number of OCN‐ and Runx2‐positive cells and the serum level of PINP; however, this mice group showed a significant decrease in Tb.Sp and bone marrow adiposity in the tibia and femur (**Figure** **8**A–K; Figure S12, Supporting Information). We further analyzed osteoblastic bone formation and osteoclastic bone resorption in *Bmi‐1^f/f^Prx1‐cre & Bmi‐1^Tg^* mice and found an increase in BV/TV and MAR; however, a decline in the number of osteoclasts and the serum level of CTX‐1 (Figure 8L–R). These results suggest that *Prx1*‐driven *Bmi‐1* overexpression in BMSCs could improve the bone mass of mice with *Prx1*‐driven *Bmi‐1* knockout by enhancing osteoblastic bone formation and inhibiting osteoclastic bone resorption.

**Figure 9:**
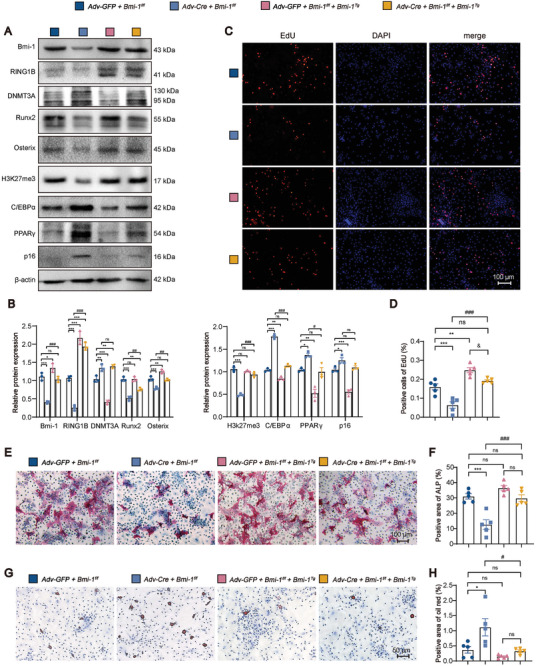
*Prx1*‐driven *Bmi‐1* overexpression increases proliferation and osteogenic differentiation but ameliorates aging and adipogenic differentiation of *Bmi‐1*‐knocked down BMSCs. Primary BMSCs extracted from *Bmi‐1^f/f^* and *Bmi‐1^f/f^Bmi‐1^Tg^* mice were transfected with Adv‐GFP or Adv‐Cre to construct *Bmi‐1^f/f^* and *Bmi‐1^f/f^Bmi‐1^Tg^* BMSCs with *Bmi‐1* knockdown or vehicle. A) Western blotting assay of BMSC extracts showing the protein levels of Bmi‐1, RING1B, DNMT3A, Runx2, Osterix, H3K27me3, C/EBPα, PPARγ, and p16. β‐Actin was used as the loading control. B) Protein expression relative to β‐actin was assessed by densitometric analysis. C) Representative micrographs of EdU staining, with nuclei staining by DAPI. D) The percentage of EdU‐positive cells. E) Representative micrographs of CFU‐fap staining. F) The percentage of ALP‐positive area. G) Representative micrographs of Oil Red staining. H) The percentage of Oil Red‐positive area. Statistical analysis was performed with one‐way ANOVA. Cell experiments were performed with three biological replicates per group. Values are expressed as mean ± SEM from five determinations per group, ^*^ *P* < 0.05, ^**^ *P* < 0.01, ^***^ *P* < 0.001, compared to the Adv‐GFP+*Bmi‐1^f/f^* group; ^#^ *P* < 0.05, ^##^ *P* < 0.01, ^###^ *P* < 0.001, compared to the Adv‐Cre+*Bmi‐1^f/f^* group; ^&^ *P* < 0.05, compared to the Adv‐GFP+*Bmi‐1^f/f^* +*Bmi‐1^Tg^* group.

To determine whether *Prx1*‐driven *Bmi‐1* overexpression increased proliferation and osteogenic differentiation and ameliorated aging and adipogenic differentiation of *Bmi‐1*‐knocked down BMSCs, we isolated primary BMSCs from *Bmi‐1^f/f^* mice and *Bmi‐1^f/f^Bmi‐1^Tg^* mice and transfected them with Adv‐Cre or Adv‐GFP. The results showed that in the Adv‐Cre‐treated *Bmi‐1^f/f^Bmi‐1^Tg^* group, the protein expression levels of Bmi‐1, RING1B, Runx2, and Osterix and the EdU‐positive area were increased; however, the DNMT3A protein level was reduced. Moreover, as compared to the Adv‐Cre‐treated *Bmi‐1^f/f^* group, the Adv‐Cre‐treated *Bmi‐1^f/f^Bmi‐1^Tg^* group exhibited increased protein levels of H3K27me3 and reduced protein levels of C/EBPα, PPARγ, and p16 (**Figure** **9**A,B). Additionally, the number of EdU‐positive cells was increased in the Adv‐Cre‐treated *Bmi‐1^f/f^Bmi‐1^Tg^* group as compared to that in the Adv‐Cre‐treated *Bmi‐1^f/f^* group. The CFU‐fap‐positive area was increased when BMSCs were induced toward osteogenic differentiation; however, the Oil Red‐positive area was decreased when BMSCs were induced toward adipogenic differentiation (Figure 9C–H). These results demonstrated that *Bmi‐1* overexpression in BMSCs rescued the SOP phenotype induced by *Bmi‐1* knockout in BMSCs, thus suggesting that Bmi‐1 acted as a hub protein in the epigenetic regulation of BMSC differentiation with aging.

## 3 Discussion

The present study demonstrated that Bmi‐1 acts as a hub protein in the epigenetic regulation of the differentiation of BMSCs to delay bone aging. *Prx1*‐driven *Bmi‐1* knockout in BMSCs did not affect bone size, but reduced bone mass and increased bone marrow adiposity by inhibiting osteoblastic bone formation, promoting osteoclastic bone resorption, downregulating proliferation and osteogenesis differentiation, and upregulating adipogenic differentiation of BMSCs. However, *Prx1*‐driven *Bmi‐1* overexpression in BMSCs showed the opposite biological phenotype to *Prx1*‐driven *Bmi‐1* knockout in BMSCs. Regarding the underlying mechanism, Bmi‐1‐RING1B bound to DNMT3A to promote its ubiquitination and inhibited DNA methylation of *Runx2* at the region from 45047012 to 45047313 bp, thus promoting the osteogenic differentiation of BMSCs. Moreover, Bmi‐1‐EZH2 repressed the transcription of *Cebpa* by promoting H3K27 trimethylation at the promoter region −1605 to −1596 bp, thus inhibiting the adipogenic differentiation of BMSCs. Finally, we found that *Prx1*‐driven *Bmi‐1* overexpression in BMSCs rescued the SOP phenotype induced by *Prx1*‐driven *Bmi‐1* knockout in BMSCs.

Previous studies on human and animal models have shown that aging was one of the main causes of osteoporosis. As a systemic skeletal disease, SOP occurs mainly due to a reduction in bone formation caused by two factors: 1) decrease in the number of BMSCs and 2) less differentiation of BMSCs into osteoblasts and more into adipocytes.^[^ ^31^ ^]^ The present study found that mice with *Prx1*‐driven *Bmi‐1* conditional knockout showed the SOP phenotype, including reduced bone mass and increased bone marrow adiposity in vivo, and decreased proliferation and osteogenic differentiation and increased adipogenic differentiation of BMSCs in vitro. The proportion of reduced bone mass gradually increased with age, and the proportion of increased bone marrow adiposity reached its peak at the age of 3 months. Thus, the loss of bone mass was caused by a decrease in the osteogenic differentiation ability, cell aging, and an increase in adipogenic differentiation of BMSCs, resulting in gradual and progressive exacerbation and showing premature bone aging and SOP.

The imbalance between bone formation by osteoblasts and bone absorption by osteoclasts causes bone mass loss.^[^ ^4^ ^]^ The present study revealed that *Prx1*‐driven *Bmi‐1* inhibited osteoclastic bone resorption and enhanced osteoblastic bone formation. Several studies have reported that osteoclast formation was regulated by macrophage colony‐stimulating factor, receptor activator of nuclear factor‐κB ligand (RANKL), and osteoprotegerin secreted by BMSCs and osteoblasts.^[^ ^32^ ^]^ Bone marrow adipocytes also increase bone resorption by secreting the osteoclast‐promoting cytokine RANKL.^[^ ^33^, ^34^ ^]^ Moreover, bone marrow precursor cells, rather than osteoblasts or osteocytes, are the most supportive cells for osteoclast formation.^[^ ^35^ ^]^ In the present study, we found that *Prx1*‐driven Bmi‐1 reduced the number and activity of osteoclasts. A recent study demonstrated that the lineage of aged skeletal stem cells promotes osteoclastic activity and myeloid skewing by affecting the activity of hematopoietic stem and progenitor cells.^[^ ^36^ ^]^ Skeletal stem cells can also form and regulate local microvascular networks, monitor the differentiation of osteoclasts, and establish and maintain the hematopoietic microenvironment required for the growth and maturation of blood cells.^[^ ^37^ ^]^ Hence, further investigations are required to determine whether *Prx1*‐driven Bmi‐1 in BMSCs could regulate the hematopoietic microenvironment and directly regulate osteoclast progenitor cells or indirectly regulate the differentiation of osteoclast progenitor cells into mature osteoclasts through osteoblasts, adipocytes, or other types of cells.

Our previous study showed that *Bmi‐1* global null mice exhibited bone growth retardation, reduced bone formation and chondrocyte proliferation, and increased adipocyte formation.^[^ ^15^ ^]^ Bmi‐1 maintains the self‐renewal of BMSCs by inhibiting the expression of p27, p16, and p19 and partially by stimulating Sirt1, thereby promoting the differentiation of BMSCs into osteoblasts and inhibiting their differentiation into adipocytes.^[^ ^15^ ^]^ Our recent study also showed that *Bmi‐1* overexpression in BMSCs restores 1,25‐dihydroxyvitamin D deficiency‐induced oxidative stress, DNA damage, cell senescence, and SASP, thereby increasing osteoblastic bone formation, bone volume, and BMD.^[^ ^38^ ^]^ A recent study indicated that Bmi‐1 was expressed in the early differentiation stage of osteoblasts and may regulate its differentiation during the endochondral osteogenesis process. The cells aggregated at embryonic day E14 are a group of mesenchymal stem cells that subsequently differentiate into chondrocytes and exhibit Bmi‐1 positivity. At E16, this group differentiates into chondrocytes expressing Bmi‐1, Runx2, and Osterix within the cartilage primordia. The function of chondrocytes in cartilage primordia is regulated by Runx2. These results suggest that Bmi‐1 may regulate the expression and transcriptional activity of Runx2 and Osterix in the endochondral osteogenesis process.^[^ ^39^ ^]^ However, it remains unclear whether and how Bmi‐1 regulates osteogenic differentiation and/or adipogenic differentiation of BMSCs. In the present study, we report for the first time that Bmi‐1 acts as a hub protein to epigenetically orchestrate osteogenic and adipogenic differentiation of BMSCs to delay bone aging. The dynamic balance between DNA methylation and demethylation is a critical factor to ensure the normal differentiation of BMSCs into osteoblasts. Abnormalities in the epigenetic mechanism of DNA methylation modification not only affect the osteogenic differentiation function of BMSCs but also lead to the development of many bone diseases.^[^ ^40^ ^]^ Several studies have demonstrated that the differentiation of BMSCs into osteoblasts was accompanied by DNA methylation in the key osteogenic gene promoter, thus suggesting that DNA methylation modification may be required for maintaining the differentiation of BMSCs into osteoblasts.^[^ ^22^, ^41^, ^42^ ^]^ Our recent study reported that Bmi‐1 significantly enhanced the E3 ubiquitin ligase activity of RING1B to promote protein ubiquitination and modify the mono‐ubiquitination of histone H2A at lysine 119.^[^ ^19^, ^20^, ^43^ ^]^ In the present study, we found that Bmi‐1 and its partner RING1B in PRC1 bound to DNMT3A to promote its ubiquitination and inhibited *Runx2* DNA methylation, thus inducing the osteogenic differentiation of BMSCs. Histone methylation regulates transcription mainly by blocking access to transcription factors and inhibiting gene transcription.^[^ ^40^ ^]^ H3K27me3, an epigenetic marker in which histone H3 is trimethylated at lysine 27, is an epigenetic silencing marker associated with the downregulation of nearby genes and is frequently found in heterochromatin regions.^[^ ^23^, ^44^ ^]^ Bmi‐1 can enhance EZH2 expression to promote H3K27 trimethylation.^[^ ^23^, ^27^ ^]^ However, the role of H3K27 in the adipogenic differentiation process is currently unclear, and its effects on *Pparg* and *Cebpa* are also unknown. The present study found that Bmi‐1 and its partner EZH2 in PRC2 repressed the transcription of *Cebpa* and *Pparg* by promoting histone H3K27 trimethylation, thereby inhibiting the adipogenic differentiation of BMSCs.

Our recent study also reported that adeno‐associated viral vector (AAV) serotype 9‐*CMV*‐*Bmi‐1*‐*RING1B* could promote the ubiquitination of GATA4 and prevent GATA4‐dependent senescence‐associated pathological cardiac hypertrophy.^[^ ^19^ ^]^ A previous study showed that eight serotypes of AAV, including 1, 4, 5, 6, 7, 9, rh10, and rh39, were successfully infected into osteoblasts in vitro. An intra‐articular injection approach was used to detect the infection efficiency of the above‐mentioned serotypes in vivo. The results indicated that only AAV9 could successfully infect bone cells in both cancellous and cortical bones.^[^ ^45^ ^]^ Further studies are required to determine whether AAV9‐*Prx1*‐*Bmi‐1*‐*RING1B* can effectively treat SOP by inhibiting DNMT3A‐dependent DNA methylation for upregulating *Runx2*.

Prx1 is a transcriptional coactivator expressed during limb sprout development and is a label for BMSCs.^[^ ^29^ ^]^ Compared to cells in the periosteum/trabeculae of adult mice, labeled cells in the periosteum are in a resting state under normal conditions. Prx1‐positive periosteal cells have important regenerative activity and are highly expressed after bone fracture, and these cells play a repair role following fractures. However, very few Prx1^+^ cells were detected in articular cartilage and growth plates.^[^ ^29^ ^]^ Consistent with this finding, we found that Prx1 was expressed in trabecular bone, bone marrow cavity, endosteum, and periosteum in adult mice, but not in articular cartilage and growth plate. Prx1 and Bmi‐1 were colocalized in trabecular bone, bone marrow cavity, endosteum, and periosteum. Hence, *Prx1*‐driven *Bmi‐1* knockout in BMSCs did not affect bone size because of no expression of *Prx1* in the growth plate. Further studies are required to investigate the biological role of periosteal cells co‐labeled with Bmi‐1 and Prx1 after fractures and whether they regulate the healing process of bone fracture.

Finally, our study demonstrated that Bmi‐1 epigenetically orchestrated osteogenic and adipogenic differentiation in BMSCs to prevent SOP. Bmi‐1‐RING1B promoted DNMT3A ubiquitination to inhibit DNA methylation of *Runx2* for increasing osteogenic differentiation. Moreover, Bmi‐1‐EZH2 inhibited the transcription of *Cebpa* and *Pparg* by promoting H3K27 trimethylation and reduced adipogenic differentiation. Thus, the *Prx1*‐driven *Bmi‐1* overexpression in BMSCs could be used as an approach for the translational therapy of SOP.

## 4 Experimental Section

### High‐Throughput Sequencing Analysis

The gene expression profile data of GSE34303 was downloaded from the Gene Expression Omnibus database (https://www.ncbi.nlm.nih.gov/geo/). The GSE34303 dataset contains the mRNA expression data of human BMSCs between passage 2 and passage 10. The DEGs in this dataset were determined using R software (version 4.0.3). Following data reprocessing and the identification of DEGs, GO analysis was performed as described previously.^[^ ^19^, ^20^ ^]^ All the genes expressed between young (passage 2) and aged (passage 10) human BMSCs were sorted according to Log2FC values. Genes that met the cut‐off criteria (*P* value ≤  0.05 and log2‐transformed fold change |Log2FC| ≥ 0.5) were indicated as DEGs using R software limma package, and a total of 815 DEGs were obtained. An unbiased gene set enrichment analysis (GSEA) was performed using the clusterProfiler package in R software as described previously.^[^ ^46^ ^]^ Please see “Supplementary table of DEGs (GSE34303)” in Supporting Information.

### Mice and Genotyping Analysis

All mice used in this study were maintained on the C57BL/6 background. The *Prx1‐cre* mice was a present from Prof. Baojian Li at Shanghai Jiao Tong University.^[^ ^29^ ^]^ Mice bred with *Prx1 cre^−/−^* male mice and *Prx1 cre^−/−^* female mice showed neonatal lethality;^[^ ^47^ ^]^ hence, *Prx1‐cre^±^* (*Prx1‐cre*) mice were used in the experiment and were bred using *Prx1‐cre^+/−^* mice and WT mice.^[^ ^29^ ^]^ *Prx1‐cre* mice were genotyped using forward primer 5ʹ‐AATCCATATTGGCAGAACGAAA‐3ʹ and reverse primer 5ʹ‐CTGACCAGAGTCATCCTTAGCG‐3ʹ.

The *Bmi‐1* heterozygous (*Bmi‐1^f/+^*) mouse line with a C57BL/6J background was constructed in the Institute of Model Animal, Wuhan University, China. The *loxp1* allele was detected using the forward primer 5ʹ‐CCCTTTTTTGTGCATAACT‐3ʹ and the reverse primer 5ʹ‐CCCTGGGACTAATTTGTATA‐3ʹ. The *loxp2* allele was detected using the forward primer 5ʹ‐GTTGCTAGGTACTTTGTATCAC‐3ʹ and the reverse primer 5ʹ‐GTCTGAATGGCCTAAATATG‐3ʹ. *Bmi‐1^f/f^* mice used in the experiment were obtained by mating male and female *Bmi‐1^f/+^* mice. To generate *Bmi‐1^f/f^Prx1‐cre* mice, female *Bmi‐1^f/f^* mice was crossed with male *Prx1‐cre* mice and mating again. For DNA amplification and analysis, two DNA ladders were used as molecular weight markers. The first DNA marker employed in this study was Biosharp (Catalog No. BL101A), which provides a size range of 100 to 1500 bp. The second marker was Takara (Catalog No. 3427Q), which offers a size range of 100 to 2000 bp for accurate sizing of PCR products.

Mice with a C57BL/6J background and showing *Bmi‐1* overexpression under the control of the 2.4‐kb *Prx1* promoter (*Bmi‐1^Tg^*) were generated at Nanjing Medical University (Nanjing, China).^[^ ^19^, ^38^, ^48^ ^]^ The primers used to identify the *Bmi‐1^Tg^* mouse genotype were as follows: forward primer: 5ʹ‐GGCTCTCTCCTTAGCTTCCC‐3ʹ; reverse primer: 5ʹ‐CCTTATGTTCAGGAGTGGTCTG‐3ʹ. To generate *Bmi‐1^f/f^Prx1‐cre+Bmi‐1^Tg^* mice, male *Bmi‐1^f/f^Prx1‐cre* mice was crossed with female *Bmi‐1^Tg^* mice.

B6/JGpt‐H11 em1Cin (CAG‐LoxP‐ZsGreen‐Stop‐LoxP‐tdTomato)/Gpt (*tdTOMATO^+^*) mice used in the experiment were purchased from GemPharmatech Co., Ltd. (Nanjing, China). Two pairs of primers were used for identifying the genotype, namely WT and 3ʹ‐arm. The WT was detected using the forward primer 5ʹ‐CAGCAAAACCTGGCTGTGGATC‐3ʹ and the reverse primer 5ʹ‐ATGAGCCACCATGTGGGTGTC‐3ʹ. The 3ʹ‐arm was detected using the forward primer 5ʹ‐TACGGCATACGAGCTGTAC‐3ʹ and the reverse primer 5ʹ‐CCAACCTTTGTTCATGGCAG‐3ʹ. *Prx1‐cre* male mice were crossed with *tdTOMATO^+^* female mice, and *tdTOMATO^+^Prx1‐cre* male mice were analyzed at the age of 8 weeks.

Mice were housed in the SPF Laboratory Animal Center of Nanjing Medical University. All experiments were conducted in accordance with the guidelines for the Care and Use of Laboratory Animals published by the US National Institutes of Health (NIH Publication, 8^th^ Edition, 2011). The use of mice for animal experiments was approved by the Institutional Animal Care and Use Committee of Nanjing Medical University (Approval Number: IACUC‐2012035).

### Primary BMSCs Culture and Adenoviral‐Cre Recombinase Infection and Differentiation

Femurs and tibias were collected from mice and flushed out BMSCs by using α‐MEM culture medium supplemented with 10% fetal bovine serum (FBS) and 1% penicillin/streptomycin solution. All nuclear cells were seeded onto 100 mm culture dishes at the density of 2 × 10^6^ cells per dish, and the dishes were incubated at 37 °C under 5% CO_2_ conditions. After 3 days, nonadherent cells were removed by washing with PBS, and the adherent cells were cultured in α‐MEM supplemented with 10% FBS and 1% penicillin/streptomycin solution for additional 5 days. Mouse BMSCs in passage 3 were used in this study.

BMSCs from *Bmi‐1^f/f^* or *Bmi‐1^f/f^Bmi‐1^Tg^* mice were infected with GFP control adenovirus (Adv‐GFP) or Cre recombinant adenovirus (Adv‐Cre) to obtain primary BMSCs in the *Bmi‐1* knockdown group (Adv‐Cre) or vehicle group (Adv‐GFP). For this purpose, BMSCs were co‐incubated with Adv‐Cre or Adv‐GFP adenovirus at a multiplicity of infection of 100 for 8 h. The obtained BMSCs were then analyzed by RT‐qPCR and Western blotting assay.

For osteogenic differentiation, the α‐MEM culture medium was supplemented with 10^−8^ m dexamethasone, 50 µg mL^−1^ vitamin C, and 10 mm β‐glycerophosphate, and the cultured cells were stained with ALP and Alizarin Red. For adipogenic differentiation, the α‐MEM culture medium was supplemented with 10^−8^ m dexamethasone and 100 µg mL^−1^ insulin, and the cultured cells were stained with Oil Red.

### The µCT Analysis

To conduct µCT analysis, the right femurs and tibias were removed and placed in a solution of periodate‐lysine‐paraformaldehyde (PLP). A CT scan was performed using the µCT system SkyScan 1176 (Bruker, Kartuizersweg, Belgium) at 9 µm resolution for quantitative analysis. 2D images were used to generate 3D renderings by using the 3D Creator software supplied with the instrument (SkyScan). The following 3D indices were calculated automatically with the software as described previously:^[^ ^15^, ^38^, ^49^ ^]^ BMD, BV/TV, Tb.N, Tb.Th, and Tb.Sp.

### Histological Analysis

The right femurs and tibias were fixed overnight at 4 °C in PLP solution, embedded in paraffin, and cut into 5‐µm‐thick sections along the coronal plane by using a rotary microtome (Leica Biosystems Nussloch GmbH, Nussloch, Germany). The paraffin‐embedded sections were rehydrated in a series of graded ethanol solutions with decreasing concentrations and ddH_2_O and subjected to hematoxylin and eosin staining, immunohistochemical staining, and immunofluorescent assay. The sections were also analyzed to determine T‐Col, ALP, and the osteoclast marker TRAP as described previously. To conduct dynamic bone formation assay, mice were intraperitoneally administered Calcein (10 mg kg^−1^; Sigma‐Aldrich, USA) and after 14 days, they were injected with XO (90 mg kg^−1^; Sigma‐Aldrich, USA). Non‐decalcified bone was embedded in an optimum cutting temperature compound, and 10‐µm‐thick sections were obtained as transparent films as described previously.^[^ ^49^, ^50^ ^]^

### Immunohistochemical Staining

Immunohistochemical staining was performed as described previously.^[^ ^51^ ^]^ The following primary antibodies were used for positive staining: anti‐Bmi‐1 (66 161, Proteintech, USA), anti‐Runx2 (BS8734, Bioworld Technology Inc., USA), anti‐Osterix (sc‐393325, Santa Cruz Biotechnology Inc., USA), and anti‐OCN (sc‐365797, Santa Cruz Biotechnology Inc.). The sections were washed, incubated with secondary antibodies (biotinylated IgG; Sigma‐Aldrich, USA), and processed using the Vectastain ABC‐HRP kit (Vector Laboratories Inc., Burlingame, CA, USA) for positive staining in accordance with the manufacturer's protocol.

### Immunofluorescent Assay

Immunofluorescent assay was performed as described previously.^[^ ^19^, ^49^ ^]^ Anti‐Bmi‐1 antibodies (10832‐1‐AP, Proteintech) were used as the primary antibodies. Affinity‐purified Alexa Fluor 594‐conjugated goat anti‐rabbit IgG (GAR5942, Multi Sciences Biotech, Co., Ltd., China) and Dylight 649‐conjugated goat anti‐rat IgG (A23640, Abbkine Scientific Co., Ltd., Wuhan, Hubei, China) were used as secondary antibodies. The nuclei were labeled with DAPI (Sigma‐Aldrich). The sections were mounted with a medium (Vector Laboratories Inc., USA) to prevent quenching as described previously.^[^ ^19^ ^]^

### Immunocytochemistry

For immunocytochemical assay, the cultured cells were incubated overnight at 4 °C with primary antibodies against 5‐MC (ab10805, Abcam, USA), followed by incubation with Alexa Fluor 594‐conjugated goat anti‐rabbit secondary antibodies to detect immunoreactivity. For the EdU assay to detect cell proliferation, cultured BMSCs were incubated with EdU for 2 h and then subjected to EdU staining by using the Apollo 567 staining kit (#C10310‐1, Guangzhou RiboBio Co., Ltd., China) in accordance with the manufacturer's instructions.

### Western Blotting Assay

Total protein was extracted from tissue/cell samples, and immunoblotting assay was performed as described previously.^[^ ^19^, ^20^ ^]^ The following primary antibodies were used: anti‐Bmi‐1 (10832‐1‐AP, Proteintech), anti‐RING1B (5694S, Cell Signaling Technology), anti‐Runx2 (GB115631‐100, Servicebio), anti‐Osterix (sc‐393325, Santa Cruz Biotechnology Inc.), anti‐DNMT3A (49768S, Cell Signaling Technology), anti‐H3K27me3 (9733S, Cell Signaling Technology), anti‐p16 (ab211542, Abcam), anti‐p53 (ab2424, Abcam), anti‐p21 (#2947, Cell Signaling Technology), anti‐C/EBPα (sc‐166258, Santa Cruz Biotechnology Inc.), and anti‐PPARγ (sc‐7273, Santa Cruz Biotechnology Inc.). β‐Actin (AP0060, Bioworld Technology Inc.) was used as a control for total protein.

### RNA Isolation and RT‐qPCR

Total RNA was extracted from cultured BMSCs by using TRIzol reagent (Invitrogen Life Sciences, USA) in accordance with the manufacturer's instructions. The cDNA was synthesized using the SuperScript III First‐Strand Synthesis SuperMix (Invitrogen). RT‐qPCR was performed using an Agilent real‐time PCR system. The β‐actin gene was simultaneously amplified to normalize gene expression. Groups of at least five mice were examined, and each experiment was repeated three times to determine differences in relative gene expression. Table S1 (Supporting Information) shows the RT‐qPCR primer sequences used in this study. Please see Table S1 (Supporting Information).

### Protein Sequence Alignment

The amino acid sequences of DNMT3A, and GATA4 proteins from mouse or humans were aligned using online tools (Uniprot/Align: https://www.uniprot.org/align/). Please see “Alignment of GATA4 and DNMT3A in Mouse or in Human” in Supporting Information.

### Ubiquitination Assay and Protein Immunoprecipitation

Based on a previously described method,^[^ ^19^, ^20^, ^52^ ^]^ HEK293T cells were transfected with full‐length plasmids, including Myc‐Ubiquitin, Flag‐DNMT3A, HA‐RING1B, and/or His‐Bmi‐1. All plasmids were produced by TranSheep Bio Co. Ltd. (Shanghai, China). Total proteins were subjected to immunoprecipitation assay using the Pierce Cross‐link Magnetic IP/Co‐IP kit (#88 805, Thermo Fisher Scientific, USA) as described previously.^[^ ^19^ ^]^ The extracted proteins were tagged with an anti‐DYKDDDDK Tag (anti‐FLAG M2 antibodies, #14 793, Cell Signaling Technology) and subjected to immunoprecipitation and Western blotting assay with anti‐ubiquitin antibodies (#91 112, Cell Signaling Technology). The input proteins were detected by western blotting assay using the following primary antibodies: anti‐DYKDDDDK tag (anti‐FLAG M2 antibodies, #14 793, Cell Signaling Technology), anti‐HA‐tag (#3724, Cell Signaling Technology), and anti‐His‐tag (#12 698, Cell Signaling Technology). BMSCs were isolated from *Bmi‐1^f/f^* mouse infected with Adv‐GFP or Adv‐Cre and from WT and *Bmi‐1^Tg^* mice and subjected to immunoprecipitation with anti‐DNMT3A (49768S, Cell Signaling Technology) antibodies. Western blotting assay was performed using anti‐ubiquitin (43124S, Cell Signaling Technology) and anti‐DNMT3A (49768S, Cell Signaling Technology) antibodies for the immunoprecipitation samples and anti‐DNMT3A (49768S, Cell Signaling Technology), anti‐Runx2 (GB115631‐100, Servicebio), anti‐Bmi‐1 (10832‐1‐AP, Proteintech), and anti‐RING1B (5694S, Cell Signaling Technology) antibodies for the input samples. β‐actin (BS6007MH, Bioworld) was used as a control for the input proteins.

### Chromatin Immunoprecipitation

Based on a previously described method,^[^ ^53^ ^]^ ChIP was performed with a Magna ChIP chromatin immunoprecipitation A kit (2 931 149, Millipore, Billerica, MA, USA) by using BMSCs from *Bmi‐1^f/f^* mouse infected with Adv‐GFP or Adv‐Cre in accordance with the manufacturer's protocol.^[^ ^19^ ^]^ Under osteogenic induction, the chromatin samples were incubated with anti‐5‐MC (VL3156961, Invitrogen), anti‐DNMT3A (49768S, Cell Signaling Technology), and anti‐rabbit IgG (PP64, Millipore, USA) antibodies. Table S2 (Supporting Information) shows the primers used for the different *Runx2* regions. Under adipogenic induction, the chromatin samples were incubated with anti‐EZH2 (5246S, Cell Signaling Technology), anti‐H3K27me3 (9733S, Cell Signaling Technology), and anti‐rabbit IgG (PP64, Millipore, USA) antibodies. Table S2 (Supporting Information) shows the primers for the different *Pparg* and *Cebpa* promoter regions. Please see Table S2 (Supporting Information).

### Motif Sequence Analysis

The JASPAR database (https://jaspar.elixir.no) was utilized for motif sequence analysis. JASPAR is an open‐access database of curated, non‐redundant transcription factor binding profiles stored as position frequency matrices and flexible models for transcription factors across multiple species in six taxonomic groups.^[^ ^54^ ^]^ Simply put, access the JASPAR database homepage, identify the research species, enter the transcription factor of interest in the search box, and obtain motif sequence. Please see “Predicting the binding sites of C/EBPβ in *Cebpa* or *Pparg* promoter” in Supporting Information.

### Dual Luciferase Assay

The chimeric genes of the *Cebpa* promoter plasmids for the transfection experiments were cloned into a GV238‐basic vector (Genechem, Shanghai, China) by ligating the luciferase gene at the 5ʹ‐flanking regions upstream of the gene. Cells of the human BMSC cell line C3H10T1/2 were plated into 24‐well cell culture plates at 24 h before transfection. A mixture of 1 µg each of *Cebpb*‐overexpressed pCDNA3.1, *Ezh2‐* overexpressed pCDNA3.1 and GV238‐basic, *Cebpb*‐overexpressed pCDNA3.1, *Ezh2‐*overexpressed pCDNA3.1 and GV238, *Ezh2*‐overexpressed pCDNA3.1 and GV238, *Cebpb*‐overexpressed pCDNA3.1, *Ezh2*‐overexpressed pCDNA3.1 and GV238‐mutation “CATGCCGTGTGATA”, *Cebpb* overexpression and GV238‐mutation was successively co‐transfected with Firefly luciferase (Fluc)‐Renilla luciferase (Rluc) into C3H10T1/2 cells by using the Lipofectamine 2000 reagent (Invitrogen) in accordance with the manufacturer's instructions as described previously.^[^ ^20^, ^53^ ^]^ After 2 days, a commercial kit (Promega Corporation, Madison, WI, USA) was used to measure the promoter‐driven luciferase activity as described previously.^[^ ^20^, ^53^ ^]^

### Statistical Analysis

All data were analyzed by GraphPad Prism software version 8.0.2 (GraphPad Software, San Diego, CA, USA) as described previously.^[^ ^49^, ^53^ ^]^ The measurement data were expressed as mean ± SEM fold‐change compared to the vehicle group and analyzed using Student's *t‐*test or one‐way ANOVA to compare differences among the groups. Student's *t‐*test was used to compare the means of two groups, and one‐way ANOVA was used to compare the means of three or more groups. Qualitative data were expressed as percentages and analyzed using the chi‐square test as indicated. *P‐*values were two‐sided, and a *P‐*value of <0.05 was considered statistically significant.

## Conflict of Interest

The authors declare no conflict of interest.

## Author Contributions

J.Z., A.C., and R.W. contributed equally to this work and should be considered co‐first authors. J.J., Y.W., and D.M. performed conceptualization. J.Y.Z., A.C., R.W., D.Q., H.C., JG.Z., J.L., T.W., Y.W., Y.L., J.W.Z., Y.D., H.Y., Y.Z., D.M., Y.W., and J.J. performed methodology. J.Y.Z., A.C., R.W., D.Q., H.C., J.G.Z., J.L., Y.W., and J.J. performed software. J.Y.Z., A.C., R.W., D.Q., H.C., T.W., Y.W., Y.L., Y.W., and J.J. performed validation. J.Y.Z., A.C., R.W., D.Q., H.C., Y.W., and J.J. performed data analysis. J.Y.Z., A.C., J.J., and Y.W. performed wrote the original draft, with help from the other authors. J.J., Y.W., Y.Z., and D.M. performed wrote, reviewed, edited the draft, with help from the other authors. J.J., R.W., and Y.W. performed project administration and supervision. J.J., Y.W., and D.M. performed funding acquisition.

## Supporting information

Supporting Information

## Data Availability

All data and materials used in the analysis are available to any researcher for purposes of reproducing or extending the analysis.
