# Supplementary material for: Bmi‐1 Epigenetically Orchestrates Osteogenic and Adipogenic Differentiation of Bone Marrow Mesenchymal Stem Cells to Delay Bone Aging
Source: Adv Sci (Weinh). 2024 Sep 3;11(46):2404518. doi: 10.1002/advs.202404518 (PMC11633582; doi:10.1002/advs.202404518)
Supplement: Supplementary file 1 — Supporting Information [file ADVS-11-2404518-s001.pdf]

ADVANCED SCIENCE Open Access

Supporting Information

for *Adv. Sci.*, DOI 10.1002/advs.202404518

Bmi-1 Epigenetically Orchestrates Osteogenic and Adipogenic Differentiation of Bone Marrow Mesenchymal Stem Cells to Delay Bone Aging

Jingyu Zhao, Ao Chen, Rong Wang, Dong Qiu, Haiyun Chen, Jiyu Li, Jin'ge Zhang, Tianxiao Wang, Yue Wang, Yujie Lin, Jiawen Zhou, Yifei Du, Hua Yuan, Yongjie Zhang, Dengshun Miao, Yuli Wang and Jianliang Jin**

SI1_Figures S1-S12 Figures and Legends

Figure S1 Physiological aging downregulates proliferation signaling, osteogenesis signaling, and PRC2 methylation signaling, but upregulates senescence and adipogenesis signaling. All the genes expressed between young (Passage 2) and aged (Passage 10) human BMSCs were sorted according to the value of log2FC to perform an unbiased gene set enrichment analysis (GSEA) analysis by the Cluster Profiler R package. Enrichment analysis about proliferation signaling including (A) positive regulation of cell cycle process and marker of proliferation Ki67 (MKI67), about (B) senescence and senescence-associated secretory phenotype-NF-κB signaling, about osteogenesis signaling including (C) osteoblast differentiation, RUNX2 regulates osteoblast differentiation, and transcriptional regulation by RUNX2, about adipogenesis signaling (D) positive regulation of lipid biosynthetic process, and about methylation signaling including (E) PRC2-EZH2-UP and PRC2 methylates histones and DNA. NES: normalized enrichment scores.

Figure S1

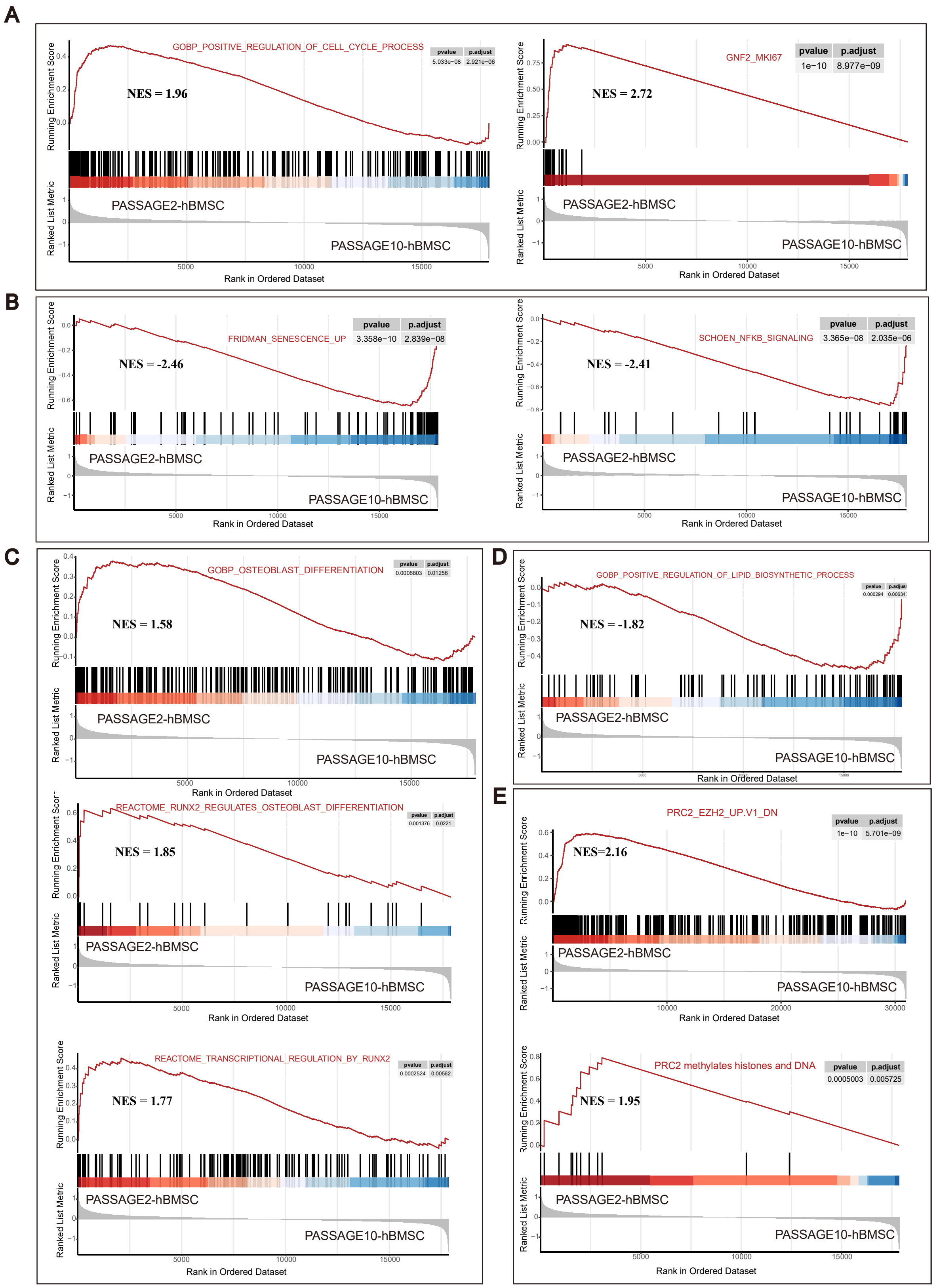
A
GOBP_POSITIVE_REGULATION_OF_CELL_CYCLE_PROCESS
pvalue p.adjust
5.033e−08 2.921e−06
NES = 1.96
GNF2_MKI67
pvalue p.adjust
1e−10 8.977e−09
NES = 2.72
B
FRIDMAN_SENESCENCE_UP
pvalue p.adjust
3.358e−10 2.839e−08
NES = -2.46
SCHOEN_NFKB_SIGNALING
pvalue p.adjust
3.365e−08 2.035e−06
NES = -2.41
C
GOBP_OSTEOBLAST_DIFFERENTIATION
pvalue p.adjust
0.0006803 0.01256
NES = 1.58
REACTOME_RUNX2_REGULATES_OSTEOBLAST_DIFFERENTIATION
pvalue p.adjust
0.001376 0.0221
NES = 1.85
REACTOME_TRANSCRIPTIONAL_REGULATION_BY_RUNX2
pvalue p.adjust
0.0002524 0.00562
NES = 1.77
D
GOBP_POSITIVE_REGULATION_OF_LIPID_BIOSYNTHETIC_PROCESS
NES = -1.82
E
PRC2_EZH2_UP.V1_DN
pvalue p.adjust
1e−10 5.701e−09
NES=2.16
PRC2 methylates histones and DNA
pvalue p.adjust
0.0005003 0.005725
NES = 1.95
Running Enrichment Score
Ranked List Metric
PASSAGE2-hBMSC
PASSAGE10-hBMSC
Rank in Ordered Dataset

Figure S2 Bmi-1 might act as a hub protein in the epigenetic regulation of differentiation of BMSCs with aging. (A) Primary second-generation cells of young BMSCs extracted from 6-week-old WT mice and primary sixth generation cells of aged BMSCs extracted from 18-month-old WT mice. The mRNA levels of *Dnmt3a, p16, p21, Cebpa* in BMSCs detected by RT-qPCR and calculated and expressed relative to control *β-actin* mRNA. (B) Western blotting assay of BMSC extracts showing the protein levels of Osterix, osteocalcin (OCN), and alkaline phosphatase (ALP). β-actin was used as a loading control. (C) Protein expression relative to β-actin was assessed by densitometric analysis. Cell experiments were performed with three or five biological replicates per group. Values are expressed as mean ± SEM from three or five determinations per group, $^{*}P < 0.05$, $^{**}P < 0.01$, $^{***}P < 0.001$, compared to the young BMSCs group, unpaired Student's *t*-test. (D) BMSCs extracted from WT mice that were induced or not induced for adipogenic differentiation. The protein levels of Bmi-1, PPARγ, C/EBPα, and EZH2 in BMSCs detected by Western blotting assay. (E) Protein expression relative to β-actin was assessed by densitometric analysis. (F) BMSCs extracted from WT mice that were induced or not induced for osteogenic differentiation. The protein levels of Bmi-1, Runx2, Osterix, ALP, and OCN in BMSCs detected by Western blotting assay. (G) Protein expression relative to β-actin was assessed by densitometric analysis. Cell experiments were performed with three biological replicates per group. Values are expressed as mean ± SEM from three determinations per group, $^{*}P < 0.05$, $^{***}P < 0.001$, compared to the uninduced BMSCs group, unpaired Student's *t*-test.

Figure S2

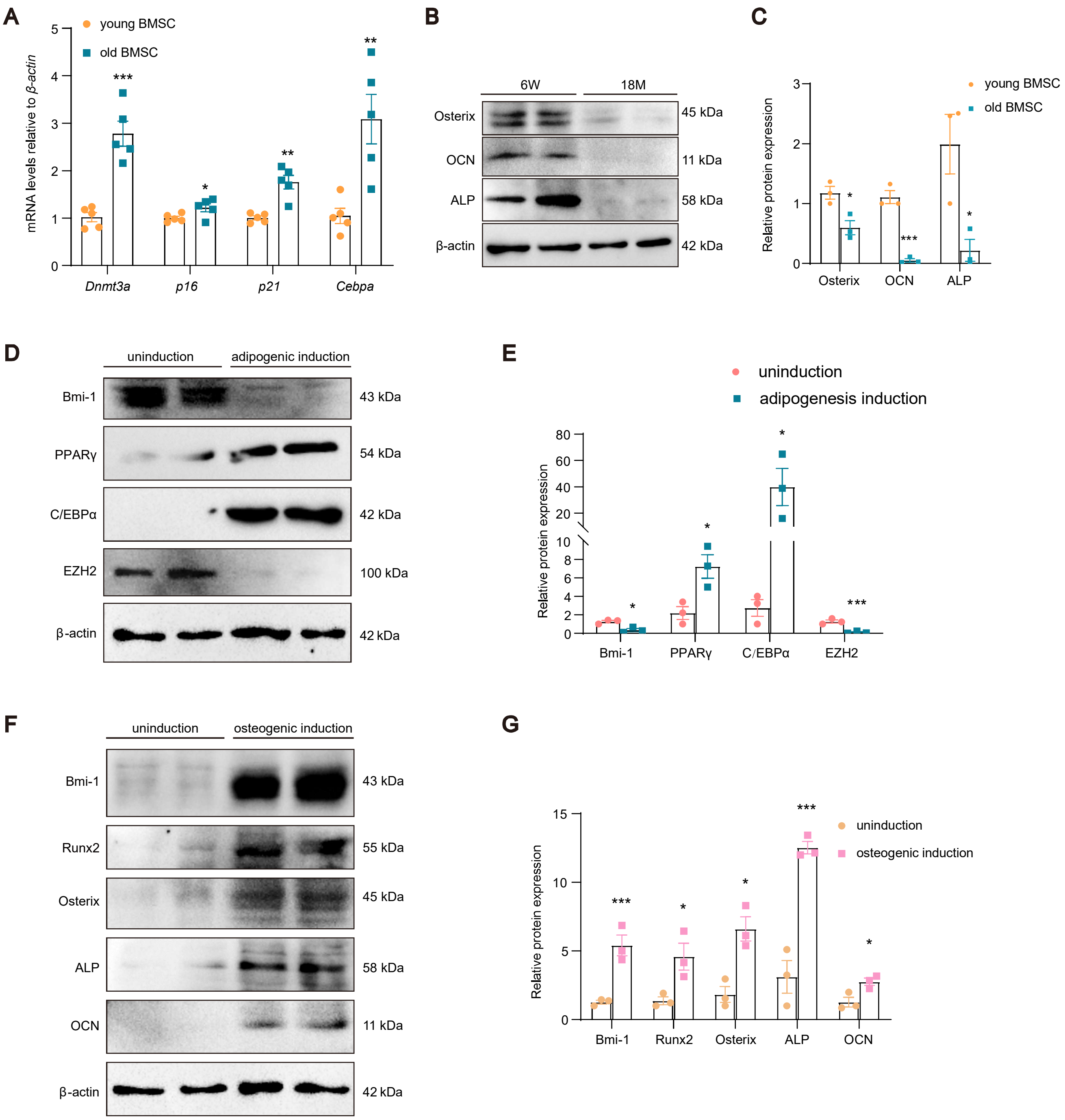

A
young BMSC
old BMSC
mRNA levels relative to β-actin
Dnmt3a
p16
p21
Cebpa
B
6W
18M
Osterix
OCN
ALP
β-actin
45 kDa
11 kDa
58 kDa
42 kDa
C
Relative protein expression
D
uninduction
adipogenic induction
Bmi-1
PPARγ
C/EBPα
EZH2
43 kDa
54 kDa
100 kDa
E
adipogenesis induction
F
osteogenic induction
Runx2
55 kDa
G

Figure S3 Genotyping of *Prx1*-driven *Bmi-1* conditional knockout mice, and *Prx1*-driven *Bmi-1* knockout in BMSCs does not affect articular cartilage and growth plate width. (A-B) Representative micrographs of Prx1- and Bmi-1 immunofluorescence-labeled cells in articular cartilage, and growth plate from 8-week-old *tdTOMATO*$^{+}$*Prx1-cre* male mice. Red fluorescence represents Prx-1; green fluorescence represents Bmi-1, with DAPI staining the nuclei. (C) Genotyping analysis of *prx1-cre*, *flox1* and *flox2*. *Prx1-cre* positive mice showed 300 bp band, however WT mice did not show this band. The *flox1* homozygous mice only showed 299 bp band; WT mice only showed 368 bp band; *flox1* heterozygous mice showed 299 bp and 368 bp bands. The *flox2* homozygous mice only showed 220 bp band; WT mice only showed 283 bp band; *flox2* heterozygous mice showed 220 bp and 283 bp bands. (D) Representative appearances of 8-week-old *Bmi-1*$^{f/f}$ and *Bmi-1*$^{f/f}$*Prx1-cre* mice. (E) Weight (g) and body length (cm). (F) Representative micrographs of Bmi-1 immunostaining in tibial sections, with DAPI staining the nuclei. (G) The percentage of Bmi-1-positive cells in primary trabecular bone, secondary trabecular bone, and total bone of tibias respectively. (H) Representative micrographs of X-ray for tibias. (I) Tibial length (cm). (J) Representative micrographs of Safranin O/fast green staining. (K-L) Quantification of tibial growth plate thickness (μm) of 6-week-old or 3-month-old mice. Five mice per group were used for experiments. Values are mean ± SEM from five determinations per group, **P < 0.01, ***P < 0.001, compared to *Bmi-1*$^{f/f}$ mice, and unpaired Student's *t*-test for bar graphs.

Figure S3

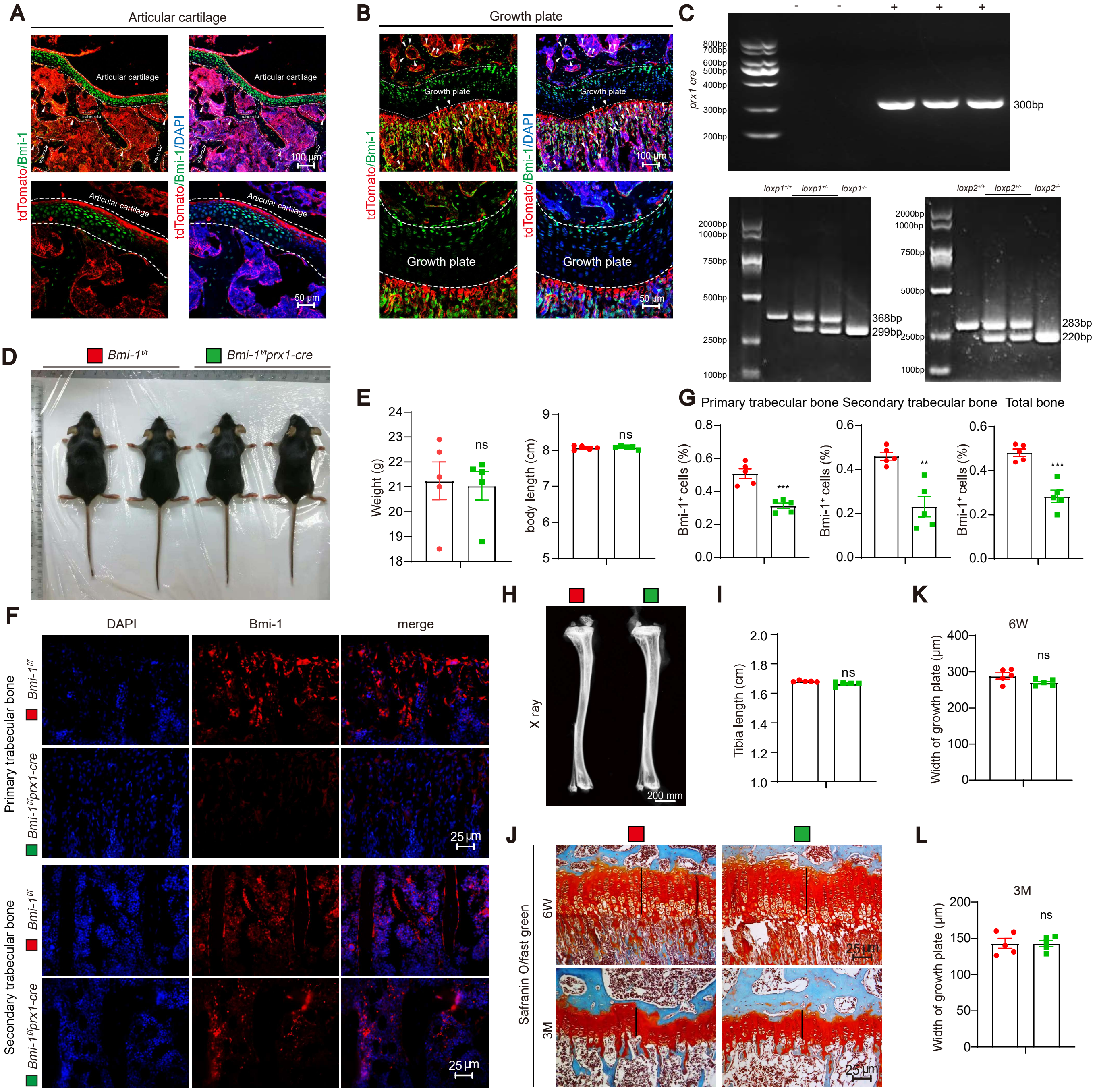
A
Articular cartilage
tdTomato/Bmi-1
tdTomato/Bmi-1/DAPI
100 μm
50 μm
B
Growth plate
C
prx1 cre
300bp
368bp
299bp
283bp
220bp
D
Bmi-1f/f
Bmi-1f/fprx1-cre
E
Weight (g)
body length (cm)
ns
G
Primary trabecular bone
Secondary trabecular bone
Total bone
Bmi-1+ cells (%)
F
DAPI
Bmi-1
merge
Primary trabecular bone
Secondary trabecular bone
25 μm
H
X ray
200 mm
I
Tibia length (cm)
K
6W
Width of growth plate (μm)
J
Safranin O/fast green
6W
3M
L
3M

Figure S4 *Prx1*-driven *Bmi-1* knockout in BMSCs reduces bone mass but increases bone marrow adiposity in femurs of 8-week-old mice. (A) Representative micrographs of X-ray for femur in 8-week-old *Bmi-1*$^{f/f}$*Prx1-cre* mice and *Bmi-1*$^{f/f}$ littermates. (B-D) Representative three-dimensional reconstruction of μCT. (E-F) Quantitative bone formation parameters analysis of bone density (BMD), trabecular number (Tb.N), trabecular thickness (Tb.Th), and trabecular separation (Tb.Sp) from proximal epiphysis and diaphysis. (G) Representative micrographs from total collagen (T-Col) staining. (H-I) Quantitative analysis of BV/TV and the percentage of adipocyte area in the proximal femur. Five mice per group were used for experiments. Values are mean ± SEM from five determinations per group, $^{***}P < 0.001$, compared to *Bmi-1*$^{f/f}$ mice, and unpaired Student's *t*-test for bar graphs.

Figure S4

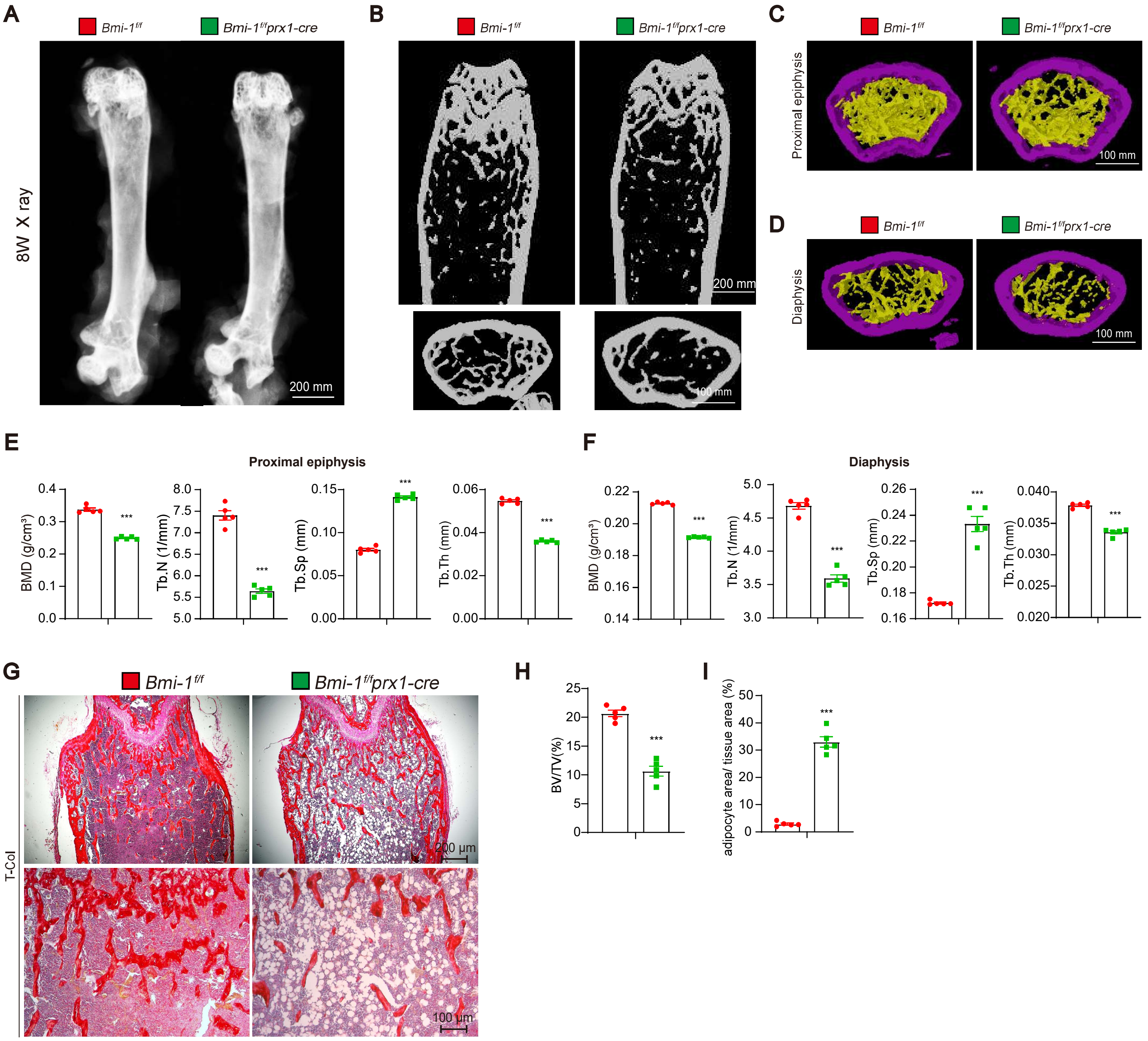

A
B
C
D
E
F
G
H
I
Bmi-1f/f
Bmi-1f/fprx1-cre
8W X ray
200 mm
200 mm
100 mm
Proximal epiphysis
Diaphysis
BMD (g/cm³)
Tb.N (1/mm)
Tb.Sp (mm)
Tb.Th (mm)
T-Col
200 μm
100 μm
BV/TV(%)
adipocyte area/ tissue area (%)
***

Figure S5 *Prx1*-driven *Bmi-1* knockout in BMSCs reduces bone mass but increases bone marrow adiposity in femurs and tibias of 21-day-old mice. (A) Representative micrographs of X-ray for tibia in 21-day-old *Bmi-1*$^{f/f}$*Prx1-cre* mice and *Bmi-1*$^{f/f}$ littermates. (B-C) Representative three-dimensional reconstruction of μCT. (D) Quantitative bone formation parameters analysis of BMD, Tb.N, Tb.Th and Tb.Sp. (E) Representative micrographs from T-Col staining. (F) Quantitative analysis of BV/TV and the percentage of adipocyte area in the proximal tibia from 21-day-old mice. (G) Representative micrographs of X-ray for femur. (H-I) Representative three-dimensional reconstruction of μCT. (J) Quantitative bone formation parameters analysis of BMD, Tb.N, Tb.Sp and Tb.Th. Five mice per group were used for experiments. Values are mean ± SEM from five determinations per group, $^{***}P < 0.001$, compared to *Bmi-1*$^{f/f}$ mice, and unpaired Student's *t*-test for bar graphs.

Figure S5

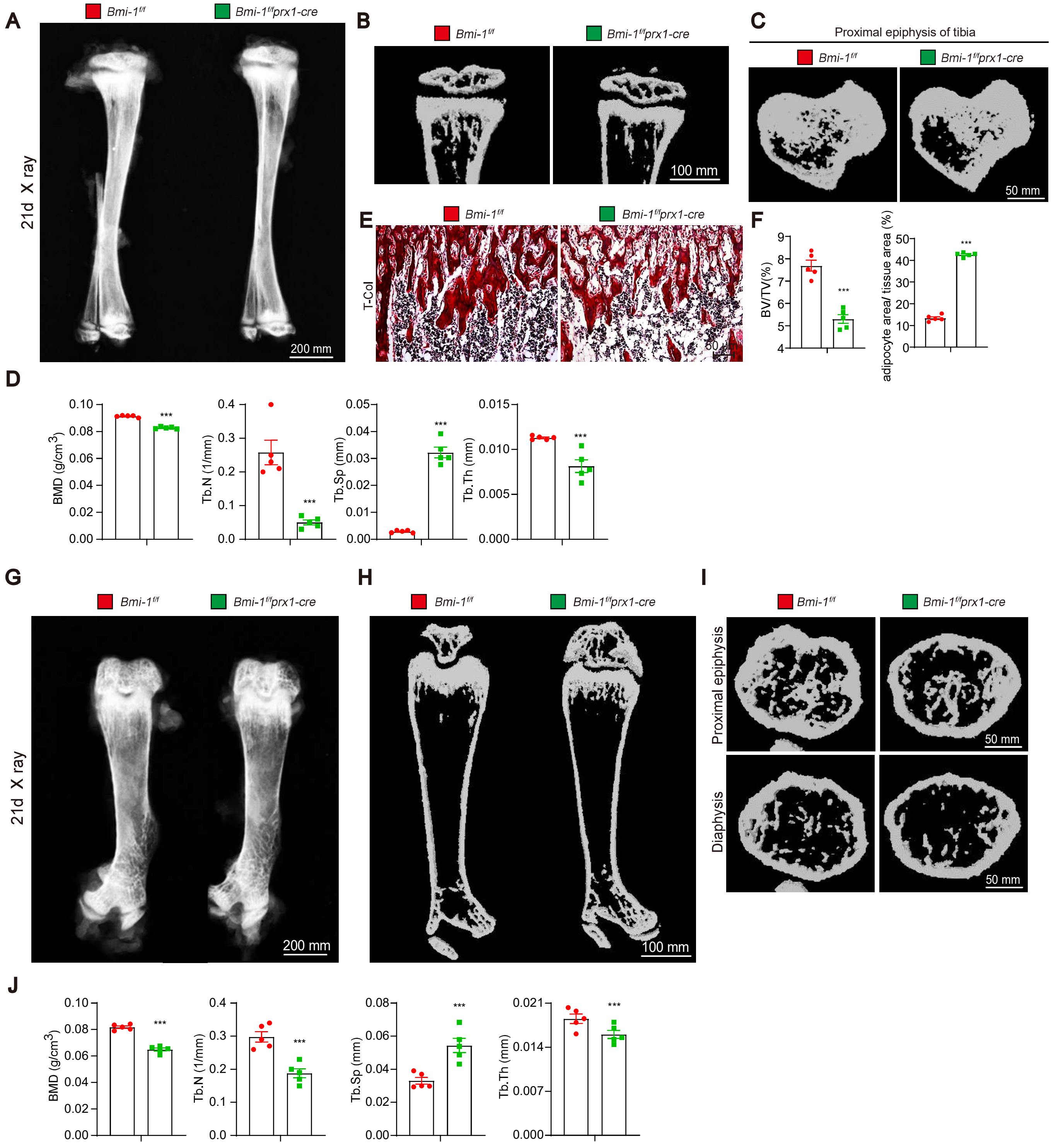

A
Bmi-1f/f
Bmi-1f/fprx1-cre
21d X ray
200 mm
B
100 mm
C
Proximal epiphysis of tibia
50 mm
E
T-Col
F
BV/TV(%)
adipocyte area/ tissue area (%)
***
D
BMD (g/cm3)
Tb.N (1/mm)
Tb.Sp (mm)
Tb.Th (mm)
G
H
I
Proximal epiphysis
Diaphysis
J

Figure S6 *Prx1*-driven *Bmi-1* knockout in BMSCs reduces bone mass but increases bone marrow adiposity in tibias of 3-month-old and 5-month-old mice. (A) Representative micrographs of X-ray for tibia in 3-month-old *Bmi-1*$^{f/f}$*Prx1-cre* mice and *Bmi-1*$^{f/f}$ littermates. (B) Representative three-dimensional reconstruction of μCT for tibia in 3-month-old mice. (C) Quantitative bone formation parameters analysis of BMD, Tb.N, Tb.Sp and Tb.Th for tibia in 3-month-old mice. (D) Representative micrographs of T-Col staining for tibia in 3-month-old mice. (E) Quantitative analysis of BV/TV and the percentage of adipocyte area in the proximal tibia from 3-month-old mice. (F) Representative micrographs of T-Col staining for tibia from 5-month-old mice. (G) Quantitative analysis of BV/TV and the percentage of adipocyte area in the proximal tibia from 5-month-old mice. Five mice per group were used for experiments. Values are mean ± SEM from five determinations per group, $^{***}P < 0.001$, compared to *Bmi-1*$^{f/f}$ mice, and unpaired Student's *t*-test for bar graphs.

Figure S6

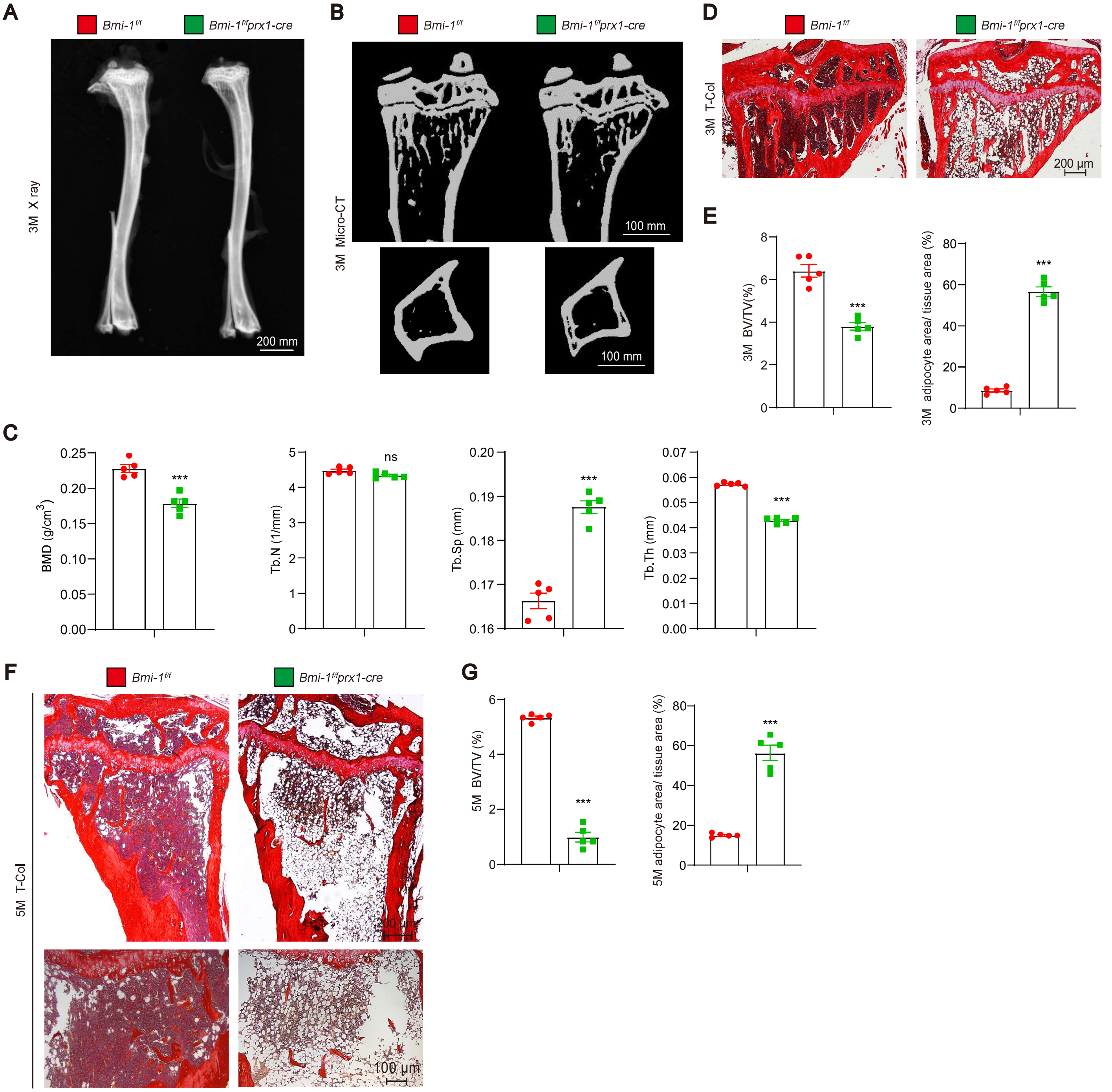

A
Bmi-1f/f
Bmi-1f/fprx1-cre
3M X ray
200 mm
B
Bmi-1f/f
Bmi-1f/fprx1-cre
3M Micro-CT
100 mm
100 mm
C
BMD (g/cm3)
Tb.N (1/mm)
ns
Tb.Sp (mm)
Tb.Th (mm)
***
D
Bmi-1f/f
Bmi-1f/fprx1-cre
3M T-Col
200 μm
E
3M BV/TV(%)
3M adipocyte area/ tissue area (%)
***
F
Bmi-1f/f
Bmi-1f/fprx1-cre
5M T-Col
100 μm
G
5M BV/TV (%)
5M adipocyte area/ tissue area (%)
***

Figure S7 *Prx1*-driven *Bmi-1* knockout in BMSCs reduces bone mass but increases bone marrow adiposity in bones of 12-14 months old mice. (A) Representative micrographs of X-ray for tibia in 12-month-old *Bmi-1*$^{f/f}$*Prx1-cre* mice and *Bmi-1*$^{f/f}$ littermates. (B) Representative three-dimensional reconstruction of μCT for the tibia in 12-month-old mice. (C) Quantitative bone formation parameters analysis of BMD, Tb.N, Tb.Sp and Tb.Th for the tibia in 12-month-old mice. (D) Representative micrographs of T-Col staining for tibia in 14-month-old mice. (E) Quantitative analysis of BV/TV and the percentage of adipocyte area in the proximal tibia from 14-month-old mice. (F) Representative micrographs of X-ray for femur in 12-month-old mice. (G-H) Representative three-dimensional reconstruction of μCT for femur in 12-month-old mice. (I) Quantitative bone formation parameters analysis of BMD, Tb.N, Tb.Sp and Tb.Th for the femur in 12-month-old mice. (J) Representative micrographs of X-ray for the vertebra in 12-month-old mice. (K) Representative three-dimensional reconstruction of μCT for vertebra in 12-month-old mice. (L) Quantitative bone formation parameters analysis of BMD, Tb.N, Tb.Th and Tb.Sp for the vertebra in 12-month-old mice. Five mice per group were used for experiments. Values are mean ± SEM from five determinations per group, $^{***}P < 0.001$, compared to *Bmi-1*$^{f/f}$ mice, and unpaired Student's *t*-test for bar graphs.

Figure S7

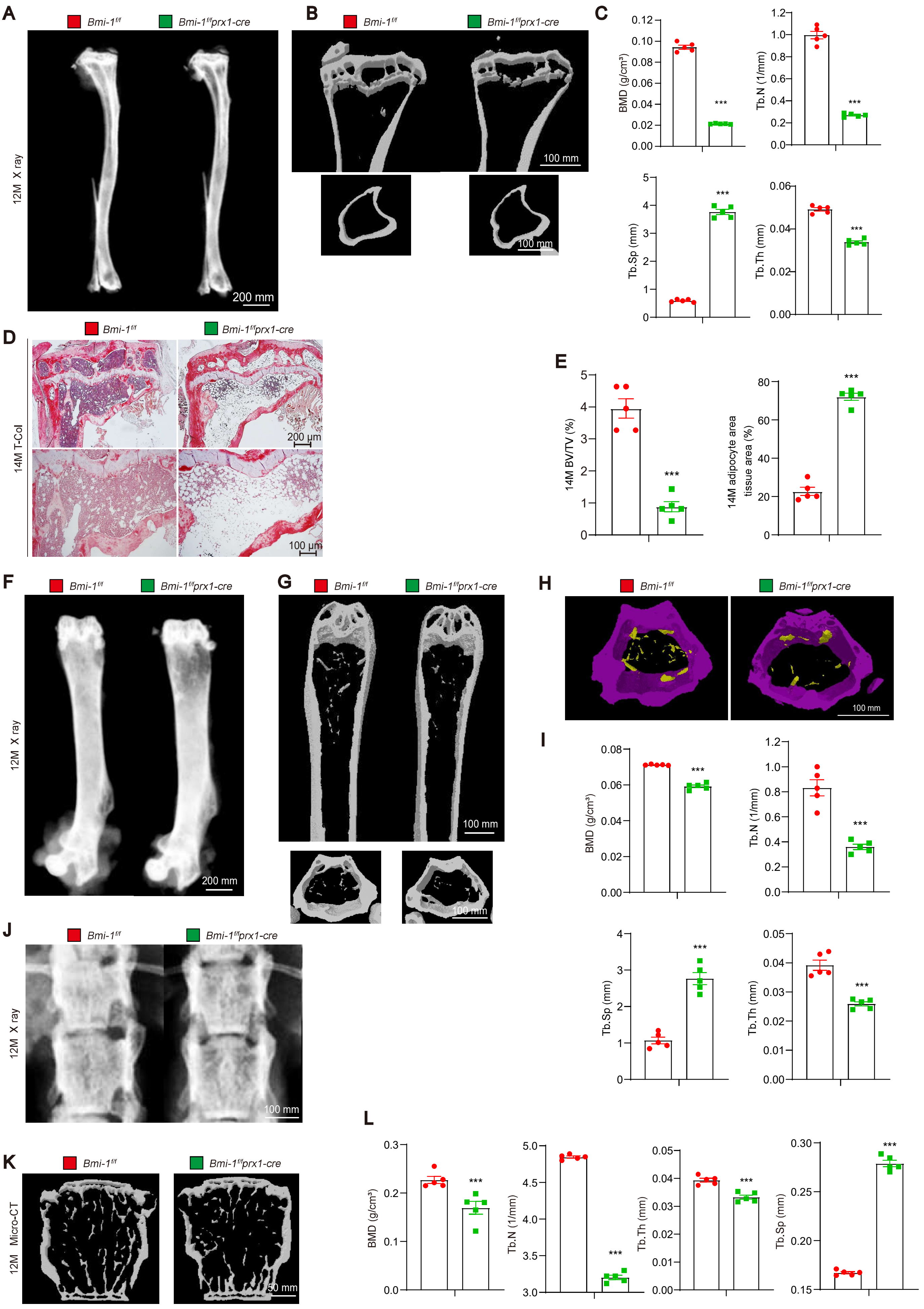

A
Bmi-1f/f
Bmi-1f/fprx1-cre
12M X ray
200 mm
B
100 mm
C
BMD (g/cm³)
Tb.N (1/mm)
Tb.Sp (mm)
Tb.Th (mm)
***
D
14M T-Col
200 μm
100 μm
E
14M BV/TV (%)
14M adipocyte area
tissue area (%)
F
G
H
I
J
K
12M Micro-CT
50 mm
L

Figure S8 *Prx1*-driven *Bmi-1* overexpression in BMSCs increases bone mass through promoting osteoblastic bone formation in tibias of 8-week-old mice. (A) Representative appearance of eight-week-old $Bmi\text{-}1^{Tg}$ and WT mice. (B) Genotyping of WT and $Bmi\text{-}1^{Tg}$ mice, and $Bmi\text{-}1^{Tg}$ positive mice showed 430 bp band, but WT mice did not show this band. (C-D) Weight and body length. (E) Representative micrographs of X-ray for tibia in eight-week-old $Bmi\text{-}1^{Tg}$ and WT mice. (F) tibia length (cm). (G) Representative micrographs of Safranin O/fast green staining. (H) Quantification of tibial growth plate thickness (μm). (I) Representative micrographs of T-Col staining for tibia. (J) Quantitative analysis of BV/TV (%) in the proximal tibia. (K) Representative micrographs of primary and secondary trabecular bone sections immunostained for OCN. (L) Quantification for the percentages of OCN-positive osteoblasts. Five mice per group were used for experiments. Values are mean ± SEM from five determinations per group, $^{*}P < 0.05$, $^{***}P < 0.001$, compared to WT mice, and unpaired Student's *t*-test for bar graphs.

Figure S8

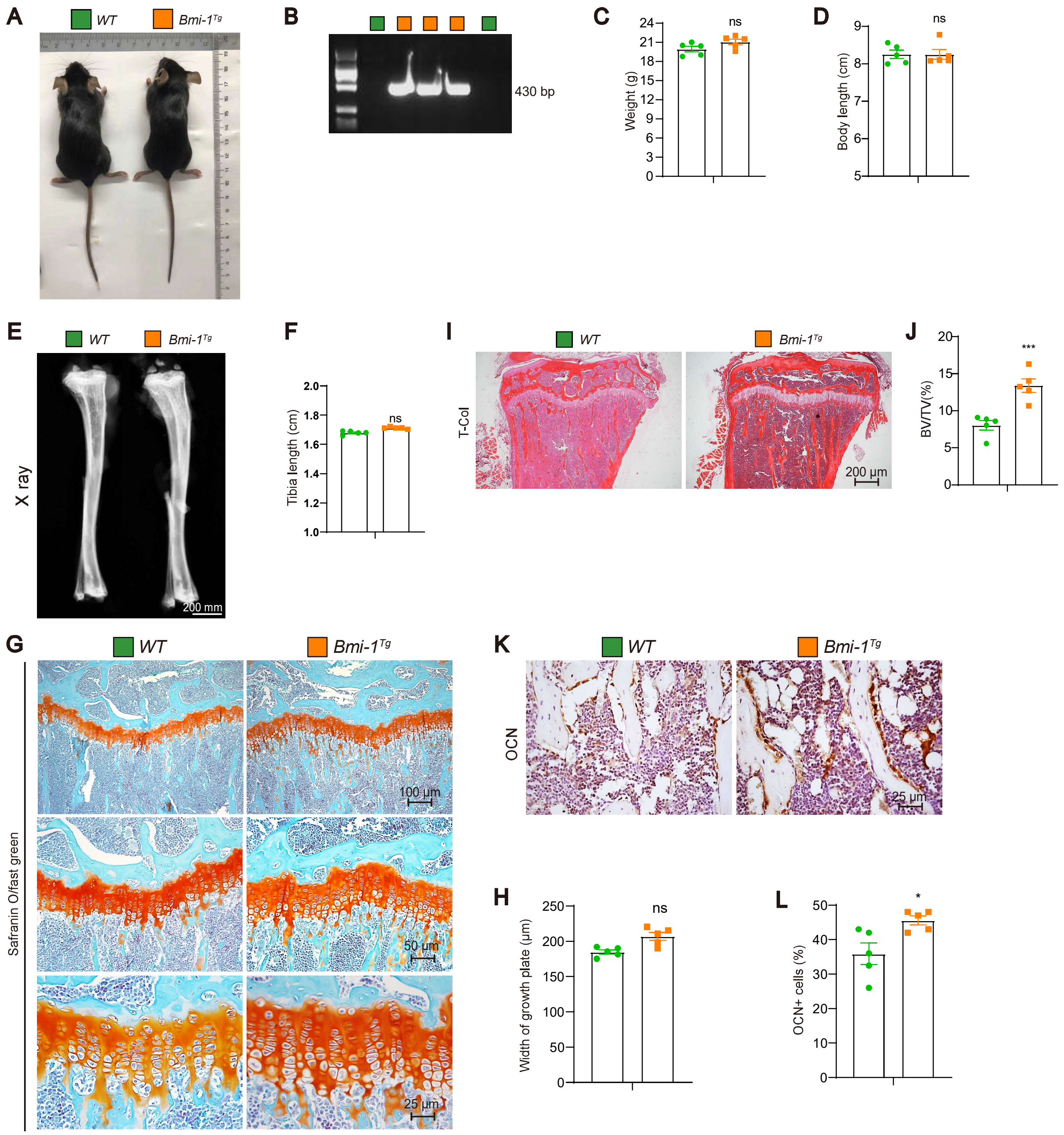

A
WT
Bmi-1Tg
B
430 bp
C
Weight (g)
ns
D
Body length (cm)
ns
E
WT
Bmi-1Tg
X ray
200 mm
F
Tibia length (cm)
ns
I
WT
Bmi-1Tg
T-Col
200 μm
J
BV/TV(%)
***
G
WT
Bmi-1Tg
Safranin O/fast green
100 μm
50 μm
25 μm
K
WT
Bmi-1Tg
OCN
25 μm
H
Width of growth plate (μm)
ns
L
OCN+ cells (%)
*

Figure S9 *Prx1*-driven *Bmi-1* overexpression in BMSCs increases bone mass through promoting osteoblastic bone formation in tibias of 7-month-old mice. (A-C) Representative three-dimensional reconstruction of μCT for tibia in 7-month-old *Bmi-1*^{Tg} and WT mice. (D-E) Quantitative bone formation parameters analysis of BMD, Tb.N, Tb.Th and Tb.Sp in proximal epiphysis or diaphysis for tibia in 7-month-old *Bmi-1*^{Tg} and WT mice. Five mice per group were used for experiments. Values are mean ± SEM from five determinations per group, $^{***}P < 0.001$, compared to WT mice, and unpaired Student's *t*-test for bar graphs.

Figure S9

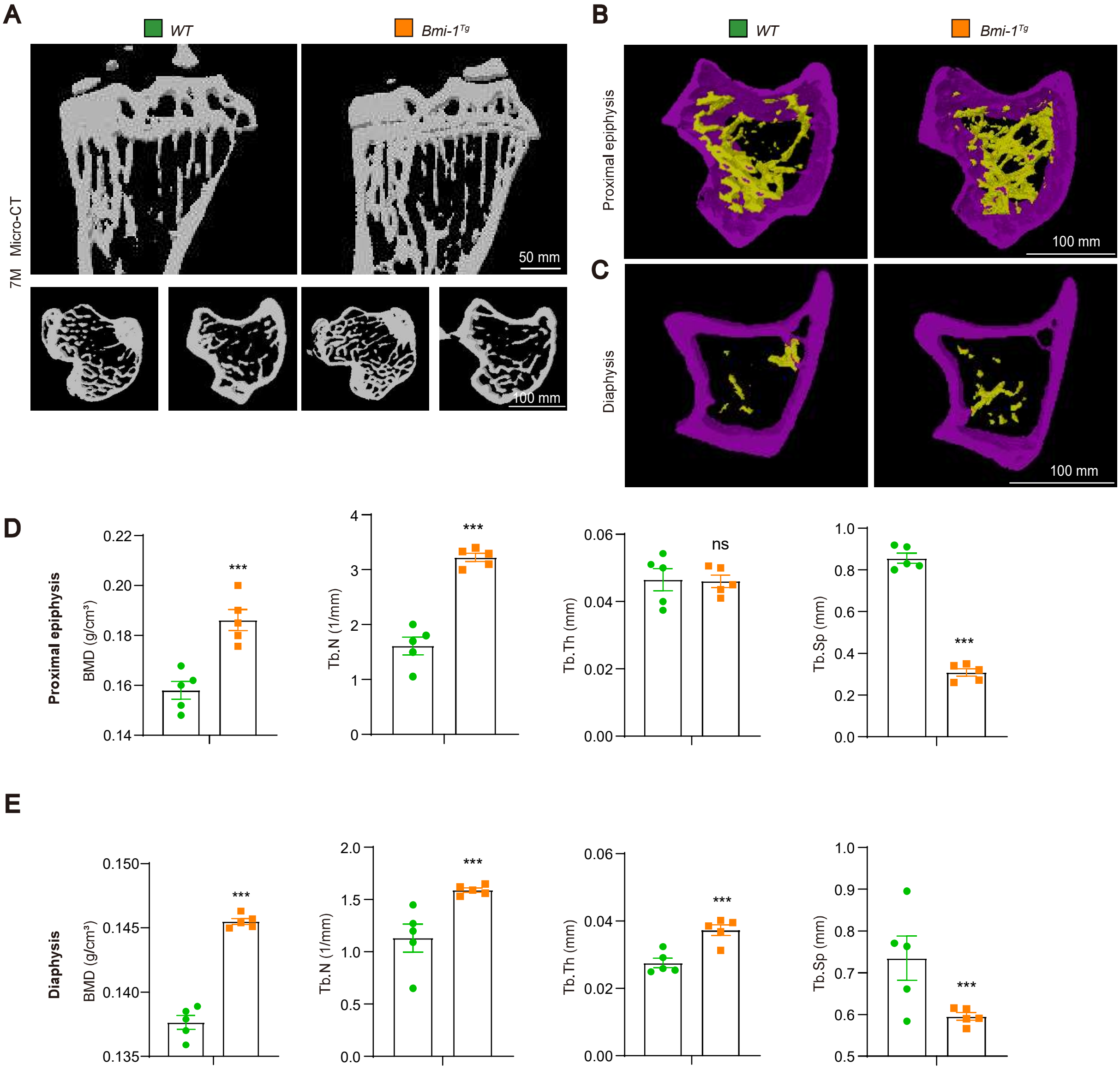

A
WT
Bmi-1Tg
7M Micro-CT
50 mm
100 mm
B
WT
Bmi-1Tg
Proximal epiphysis
100 mm
C
Diaphysis
100 mm
D
Proximal epiphysis
BMD (g/cm³)
Tb.N (1/mm)
Tb.Th (mm)
Tb.Sp (mm)
***
ns
E
Diaphysis
BMD (g/cm³)
Tb.N (1/mm)
Tb.Th (mm)
Tb.Sp (mm)
***

Figure S10 *Prx1*-driven *Bmi-1* overexpression in BMSCs increases bone mass through promoting osteoblastic bone formation in tibias of 12-month-old mice. (A-B) Representative three-dimensional reconstruction of μCT for tibia in 12-month-old *Bmi-1*^{Tg} and WT mice. (C) Quantitative bone formation parameters analysis of BMD, Tb.N, Tb.Sp and Tb.Th for tibia in 12-month-old *Bmi-1*^{Tg} and WT mice. (D-E) Representative three-dimensional reconstruction of μCT for femur in 12-month-old *Bmi-1*^{Tg} and WT mice. (F) Quantitative bone formation parameters analysis of BMD, Tb.N, Tb.Sp and Tb.Th for femur in 12-month-old *Bmi-1*^{Tg} and WT mice. Five mice per group were used for experiments. Values are mean ± SEM from five determinations per group, $^{***}P < 0.001$, compared to WT mice, and unpaired Student's *t*-test for bar graphs.

Figure S10

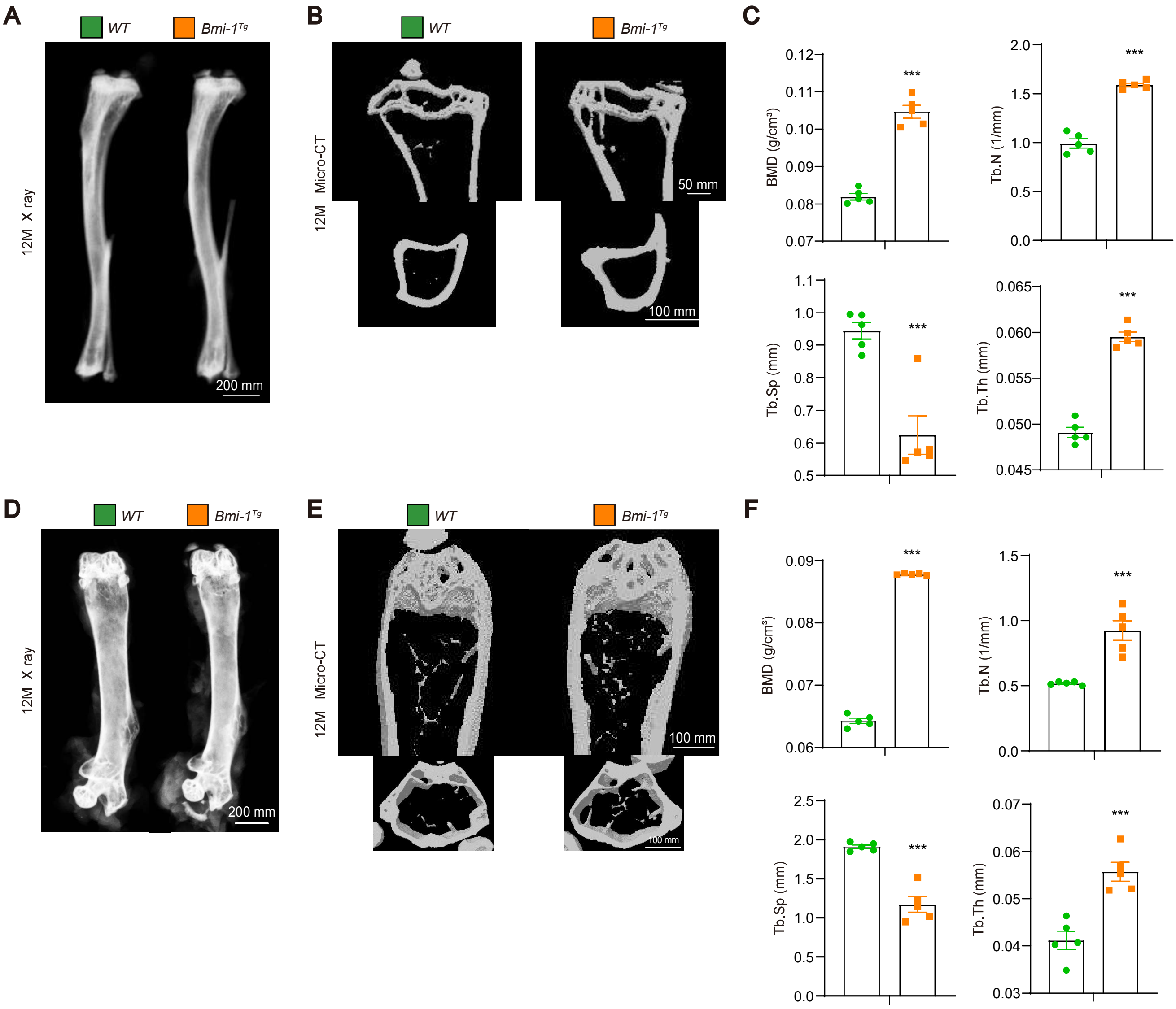
A
WT
Bmi-1Tg
12M X ray
200 mm
B
WT
Bmi-1Tg
12M Micro-CT
50 mm
100 mm
C
BMD (g/cm³)
Tb.N (1/mm)
Tb.Sp (mm)
Tb.Th (mm)
***
D
WT
Bmi-1Tg
12M X ray
200 mm
E
WT
Bmi-1Tg
12M Micro-CT
100 mm
F
BMD (g/cm³)
Tb.N (1/mm)
Tb.Sp (mm)
Tb.Th (mm)
***

Figure S11 Bmi-1-EZH2 represses the transcription of *PPARγ* by promoting H3K27 trimethylation at promoter region. Primary BMSCs extracted from *Bmi-1$^{f/f}$* mice were transfected with GFP control adenovirus or Cre recombinant adenovirus to construct the primary BMSCs with *Bmi-1* knocked-down group (Adv-Cre) or vehicle group (Adv-GFP). (A) An illustration of *Pparg* promoter truncated primers. (B-C) ChIP assays were performed with chromatin prepared from BMSCs. The chromatin was immunoprecipitated with normal rabbit IgG or antibodies against EZH2 and H3K27me3, and precipitated genomic DNA was analyzed as relative enrichment using qPCR and different primers for the different regions of the *Pparg* promoter. Cell experiments were performed with three biological repetitions per group. Values are mean ± SEM from three determinations per group, $^{*}P<0.05$, $^{**}P <0.01$, $^{***}P<0.001$, compared to the Adv-GFP group, unpaired Student's *t*-test for bar graphs.

Figure S11

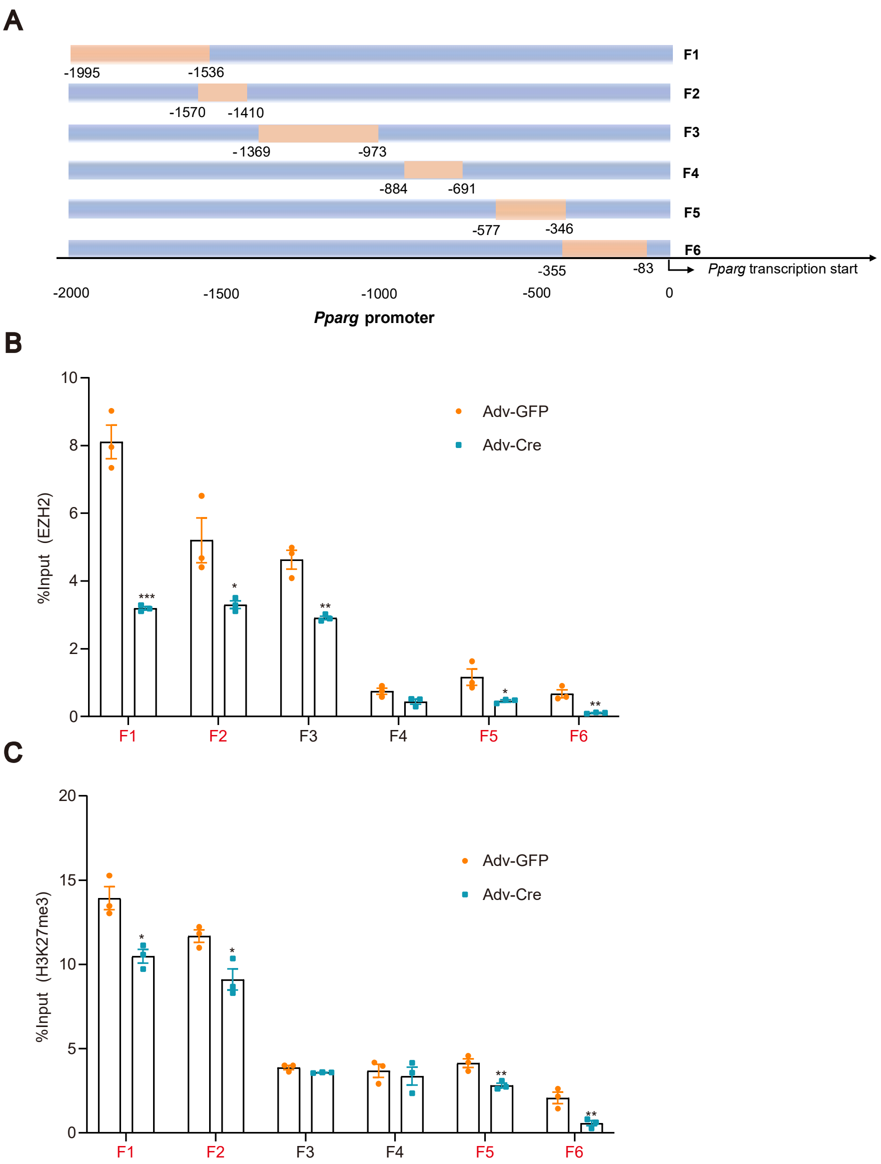

A
F1
-1995
-1536
F2
-1570
-1410
F3
-1369
-973
F4
-884
-691
F5
-577
-346
F6
-355
-83
Pparg transcription start
-2000
-1500
-1000
-500
0
Pparg promoter
B
%Input (EZH2)
Adv-GFP
Adv-Cre
***
*
**
*
**
F1
F2
F3
F4
F5
F6
C
%Input (H3K27me3)
Adv-GFP
Adv-Cre
*
*
**
**
F1
F2
F3
F4
F5
F6

Figure S12 *Prx1*-driven *Bmi-1* overexpression in BMSCs rescues the SOP phenotype in femurs induced by *Prx1*-driven *Bmi-1* knockout in MSCs. The *Bmi-1*$^{f/f}$*Prx1-cre* with *Bmi-1*^{Tg} (*Bmi-1*$^{f/f}$*Prx1-cre & Bmi-1*^{Tg}) mice were generated, and the femur phenotype was compared with that of *Bmi-1*$^{f/f}$ or *Bmi-1*$^{f/f}$ *Prx1-cre* mice. (A-C) Representative three-dimensional reconstruction of μCT. (D-E) Quantitative bone formation parameters analysis of BMD, Tb.N, Tb.Th and Tb.Sp from proximal epiphysis and diaphysis. (F) Quantification of femur length (cm). (G) Representative micrographs from T-Col staining. (H) Quantitative analysis of BV/TV (%) and the percentage of adipocyte area in the proximal femur. (I) Representative micrographs of metaphysis, primary and secondary trabecular bone sections stained with H&E and (J) a quantitative analysis of the number of osteoblasts per tissue area (N.Ob/T.Ar, #/mm^2). (K) Representative micrographs of femur sections immunostained for OCN and Runx2. (L) Quantification for the percentages of OCN- or Runx2-positive osteoblasts. Five mice per group were used for experiments. Statistical analysis was performed with One-way ANOVA test. Values are mean ± SEM from five determinations per group, $^{*}P<0.05$, $^{**}P<0.01$, $^{***}P<0.001$, compared to *Bmi-1*$^{f/f}$ mice. $^{\#}P<0.05$, $^{\#\#}P<0.01$, $^{\#\#\#}P<0.001$, compared to *Bmi-1*$^{f/f}$*Prx1-cre* mice.

Figure S12

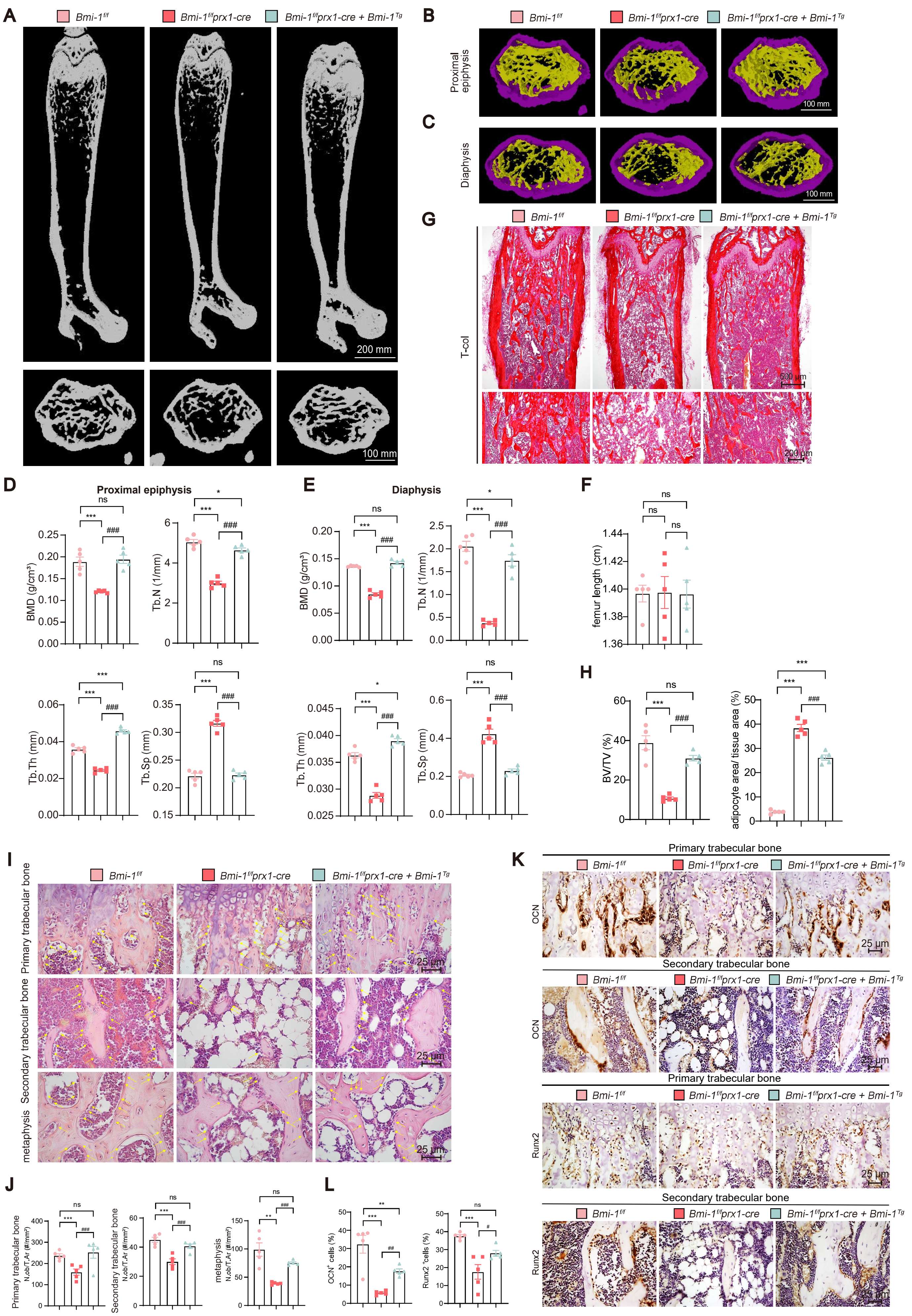

A
Bmi-1f/f
Bmi-1f/fprx1-cre
Bmi-1f/fprx1-cre + Bmi-1Tg
200 mm
100 mm
B
Proximal epiphysis
C
Diaphysis
100 mm
G
T-col
500 μm
200 μm
D
Proximal epiphysis
BMD (g/cm3)
Tb.N (1/mm)
Tb.Th (mm)
Tb.Sp (mm)
E
Diaphysis
F
femur length (cm)
H
BV/TV (%)
adipocyte area/ tissue area (%)
I
Primary trabecular bone
Secondary trabecular bone
metaphysis
25 μm
J
Primary trabecular bone N.ob/T.Ar (#/mm2)
Secondary trabecular bone N.ob/T.Ar (#/mm2)
metaphysis N.ob/T.Ar (#/mm2)
K
Primary trabecular bone
Secondary trabecular bone
OCN
Runx2
25 μm
L
OCN+ cells (%)
Runx2+cells (%)

SI2_Graphical Abstract Figure

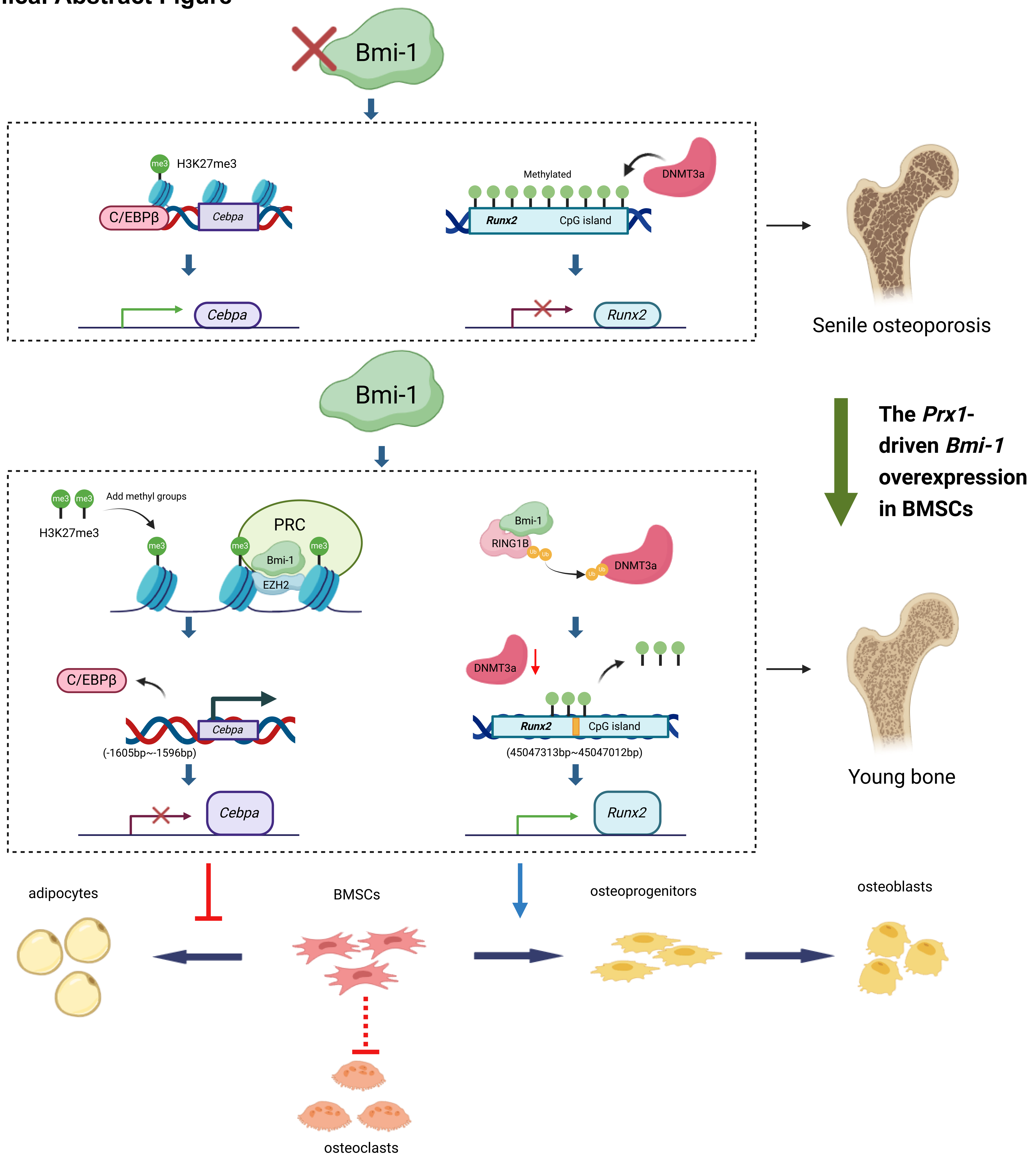

SI3_Graphical Abstract Legend

Bmi-1 epigenetically orchestrated osteogenic and adipogenic differentiation of bone-marrow mesenchymal stem cells (BMSCs) to prevent senile osteoporosis (SOP). Bmi-1-RING1B bound to DNMT3A and promoted its ubiquitination and inhibited DNA methylation of *Runx2* at the region from 45047012 to 45047313 bp, thus promoting the osteogenic differentiation of BMSCs. Moreover, Bmi-1-EZH2 repressed the transcription of *Cebpa* by promoting H3K27 trimethylation at the promoter region -1,605 to -1,596 bp, thus inhibiting the adipogenic differentiation of BMSCs. *Prx1*-driven *Bmi-1* overexpression in BMSCs rescued the SOP phenotype induced by *Prx1*-driven *Bmi-1* knockout in BMSCs. Moreover, BMSCs differentiation regulated by Bmi-1 could reduce osteoclast number and activity for further preventing SOP. (Image created with BioRender.com)

SI4_Table S1 Primers for RT-qPCR

Name	S/AS	Sequence	Primer(bp)	Species	Tm (°C)	Length (bp)
alp	S	5'-ACCATTCCCACGTCTTCACATTT-3'	23	mouse	55	162
	AS	5'-AGACATTCTCTCGTTCACCGCC-3'	22			
Bglap	S	5'-CAGACAAGTCCCACACAGCAGC-3'	22	mouse	60	181
	AS	5'-TGTTCACTACCTTATTGCCCTCC-3'	23			
Sp7	S	5'-AGAGGTTCACTCGCTCTGACGA-3'	22	mouse	55	115
	AS	5'-TTGCTCAAGTGGTCGCTTCTG-3'	21			
Pparg	S	5'-CGAGACCAACAGCTTCTCCTTCTCG-3'	25	mouse	55	90
	AS	5'-TTTCAGAAATGCCTTGCAGTGGT-3'	23			
Cebpa	S	5'-CAAGAACAGCAACGAGTACCG-3'	21	mouse	55	124
	AS	5'-GTCACTGGTCAACTCCAGCAC-3'	21			
Cd36	S	5'-CGATTAACATAAGTAAAGTTGCCATA-3'	26	mouse	60	78
	AS	5'-CGCAGTGACTTTCCCAATAGGAC-3'	23			
Bmi-1	S	5'-GATGCTGCCAATGGCTCCAA-3'	20	mouse	60	186
	AS	5'-TGCTGGGCATCGTAAGTACC-3'	20			
Ezh2	S	5'-AGTGACTTGGATTTTCCAGCAC-3'	22	mouse	58	100
	AS	5'-AATTCTGTTGTAAGGGCGACC-3'	21			
p21	S	5'-CCTGGTGATGTCCGACCTG-3'	19	mouse	55	103
	AS	5'-CCATGAGCGCATCGCAATC-3'	19			
p16	S	5'-CGCAGGTTCTTGGTCACTGT-3'	20	mouse	55	127
	AS	5'-TGTTCACGAAAGCCAGAGCG-3'	20			
Dnmt3a	S	5'-GAGGGAACTGAGACCCCAC-3'	19	mouse	55	216
	AS	5'- CTGGAAGGTGAGTCTTGGCA-3'	20			
β-Actin	S	5'-GGCTGTATTCCCCTCCATCG-3'	20	mouse	60	154
	AS	5'-CCAGTTGGTAACAATGCCATGT-3'	22			

S, sense; AS, antisense, sequence; Tm, annealing temperature; length, amplicon

SI5_Table S2 Primers for ChIP-qPCR

Name	S/AS	Sequence	Primer (bp)	Species	Tm (°C)	Length (bp)
Runx2 F1	S	5’-CACCAGCTACGAGGAAGTCG-3’	20	mouse	55	412
9328~8917bp	AS	5’-ATCGCTGTGCTCAGATGGTC-3’	20			
Runx2 F2	S	5’-CTCTGTCCACTCCTCAACCCC-3’	21	mouse	55	252
8993~8742bp	AS	5’-GTAGCCATTAATTTGGGGCTTT-3’	22			
Runx2 F3	S	5’-CCAAATCACAGTTGCGTCA-3’	19	mouse	55	484
8608~8125bp	AS	5’-GAATCCCCAAAAGATTGTGGT-3’	21			
Runx2 F4	S	5’-TCTCAGCTTTAGCGTCGTCA-3’	20	mouse	55	242
7539~7298bp	AS	5’-AAGGTGCCGGGAGGTAAGT-3’	19			
Runx2 F5	S	5’-TACCTCCCGGCACCTTGAAA-3’	20	mouse	55	302
7313~7012bp	AS	5’-GGTTCAGCAGTCCATCTCGG-3’	20			
Runx2 F6	S	5’-CCTGCGGGGCTCCACTAC-3’	18	mouse	55	452
7029~6578bp	AS	5’-GCATCACAACAGCCACAAGTTA-3’	22			
Runx2 F7	S	5’-CTTGTGGCTGTTGTGATGCG-3’	20	mouse	55	443
6596~6154bp	AS	5’-GGGGGTGACCAGTCTCTTAC-3’	20			
Runx2 F8	S	5’-ATCCTTCAGTCTTCCAGGCG-3’	20	mouse	55	229
5953~5725bp	AS	5’-GGGACACCTAGTTCGGGGTA-3’	20			
Runx2 F9	S	5’-GTCCCTTTCCCGGATTGGTT-3’	20	mouse	55	264
5729~5466bp	AS	5’-CTGCCTGGCCCTGATAGAAG-3’	20			
Runx2 F10	S	5’-CCGGGGTTTCGGCTTCTATC-3’	20	mouse	55	183
5485~5303bp	AS	5’-CCAACCGAAGTTGCTCCACA-3’	20			
Pparg F1	S	5’-TCTAACCCACTGAAGGCAGTAGA-3’	23	mouse	55	460
-1995~-1536bp	AS	5’-TATTCTCCTAAGGCCAGCCCA-3’	21			
Pparg F2	S	5’-ACTGACACAAGGGATGGGCTG-3’	21	mouse	55	161
-1570~-1410bp	AS	5’-AAGTAGCCAACTTCTTGCTCACC-3’	23			
Pparg F3	S	5’-GTGGGATGCTGACCCAAGTC-3’	20	mouse	55	397
-1369~-973bp	AS	5’-TGGTACAAACCATCCTCACCA-3’	21			
Pparg F4	S	5’-AGAGGCCAGTGCCTCCTAAAG-3’	21	mouse	55	194
-884~-691bp	AS	5’-CCTGAAGCCCACATGATAGACA-3’	22			
Pparg F5	S	5’-AGCCAGTGAACAAGGTTAACAA-3’	22	mouse	55	232
-577~-346bp	AS	5’-GCGAATGCCTTAATCAACAGACT-3’	23			
Pparg F6	S	5’-AGGCATTCGCCTTCATAACATT-3’	22	mouse	55	273
-355~-83bp	AS	5’-CGATGTCCATGTCCATGTCTGT-3’	22			
Cebpα F1	S	5’-GGATGTCAGCCTCCTGCTAAT-3’	21	mouse	55	227
-1922~-1695bp	AS	5’-AAACAACCTGGTGTGAGCAGA-3’	21			
Cebpα F2	S	5’-TTTTTATGCGCTCTGCACCG-3’	20	mouse	55	285
-1637~-1353bp	AS	5’-TCCATTTGTCCGTGTTCCGT-3’	20			
Cebpα F3	S	5’-GGTCTTCGGGGTGCAAAAAC-3’	20	mouse	55	245
-1315~-1071bp	AS	5’-TGGGGGCACTAATCTCGGTA-3’	20			
Cebpα F4	S	5’-GGGGATCACAGTCCCCGATT-3’	20	mouse	55	178
-1012~-835bp	AS	5’-GGCTTACCAGGCAGACGTT-3’	19			
Cebpα F5	S	5’-AGCAATCCTATCGCTCTGGC-3’	20	mouse	55	247

-832~-586bp	AS	5’-TTCTCTGAACTCCACGCGAC-3’	20			
Cebpα F6	S	5’-CGCTAGGTTGCTGGTCCAAA-3’	20	mouse	55	238
-538~-301bp	AS	5’-GCCTCTAAGTCACCCACTTCC-3’	22			
Cebpα F7	S	5’-GGAAGTGGGTGACTTAGAGGC-3’	21	mouse	55	192
-321~-130bp	AS	5’-ACTTTCCAAGGCGGTGAGTG-3’	20			

S, sense; AS, antisense, sequence; Tm, annealing temperature; length, amplicon

SI6_Alignment of GATA4 and DNMT3A in Mouse or in Human

1.Alignment of GATA4 and DNMT3A in Mouse

```
CLUSTAL O(1.2.4) multiple sequence alignment


sp|Q08369|GATA4_MOUSE      ------------------------------------------------------------ 0
sp|O88508|DNM3A_MOUSE      MPSSGPGDTSSSSLEREDDRKEGEEQEENRGKEERQEPSATARKVGRPGRKRKHPPVESS 60


sp|Q08369|GATA4_MOUSE      --------MYQSLAMAANHGPP---------------------P---------------- 15
sp|O88508|DNM3A_MOUSE      DTPKDPAVTTKSQPMAQDSGPSDLLPNGDLEKRSEPQPEEGSPAAGQKGGAPAEGEGTET 120
                                     :*  ** : **

sp|Q08369|GATA4_MOUSE      ------------------------------------------------------------ 15
sp|O88508|DNM3A_MOUSE      PPEASRAVENGCCVTKEGRGASAGEGKEQKQTNIESMKMEGSRGRLRGGLGWESSLRQRP 180


sp|Q08369|GATA4_MOUSE      ---GAYEAGGPGAFM-------------------------------------HSAGAASS 35
sp|O88508|DNM3A_MOUSE      MPRLTFQAGDPYYISKRKRDEWLARWKREAEKKAKVIAVMNAVEENQASGESQKVE-EAS 239
                               :::**.*  :                                     :..   :*

sp|Q08369|GATA4_MOUSE      PVYVPTPRVPSS--VLGLSYLQGGGSAAAAGTTSGGSSGAGPSGAGPGTQQGSPGWSQ-- 91
sp|O88508|DNM3A_MOUSE      PPAVQQPTDPASPTVATTPEPVGGDAGDKNATKAADD--EPEYEDGRGFGIGELVWGKLR 297
                           *  *  *  *:*  *       **.:.   .*.:...        * *   *.  *.:

sp|Q08369|GATA4_MOUSE      --------------AGA----EGA--------------------------------AYTP 101
sp|O88508|DNM3A_MOUSE      GFSWWPGRIVSWWMTGRSRAAEGTRWVMWFGDGKFSVVCVEKLMPLSSFCSAFHQATYNK 357
                                         :*     **:                                :*.

sp|Q08369|GATA4_MOUSE      PPVSPRFSFP------GTTGSLAAAAAAAAAREAAAYGSGGG-------AAGAGLAGREQ 148
sp|O88508|DNM3A_MOUSE      QPMYRKAIYEVLQVASSRAGKLFPAC-----HDSDESDSGKAVEVQNKQMIEWALGGFQP 412
                            *:  :  :       . :*.*  *.     :::   .** .           .*.* :

sp|Q08369|GATA4_MOUSE      YGRPGFAG--SYSSPYPAYMADVGASWAAAAAASAGPFDSPVLHSLPGRANPGRHPNL-D 205
sp|O88508|DNM3A_MOUSE      SGPKGLEPPEEEKNPYKEV---YTDMWVEPEAAAYAP---PPPAKKPRKSTT-EKPKVKE 465
                            *  *:    . ..**          *.   **: .*   *   . * ::.  .:*:: :

sp|Q08369|GATA4_MOUSE      MFDD--------------FSEGRECVNCGAMSTPLW-----------------------R 228
sp|O88508|DNM3A_MOUSE      IIDERTRERLVYEVRQKCRNIEDICISCGSLNVTLEHPLFIGGMCQNCKNCFLECAYQYD 525
                           ::*:               .    *:.**::.. *

sp|Q08369|GATA4_MOUSE      RDGTGHYLCNACGLYHKMNGINRPLIKPQRRLSASRRVGLSCANCQTTTT---TLWRRNA 285
sp|O88508|DNM3A_MOUSE      DDGYQSYCTICCGGREVLMCGN------------NNCCRCFCVECVDLLVGPGAAQAAIK 573
                            **   *   .**  . :   *            ..     *.:*    .   :

sp|Q08369|GATA4_MOUSE      EGEPVCNACGL---YMK---LHGVPRPLAMRKEGIQTRKRKPKNLNKSKTPAGPAGETLP 339
sp|O88508|DNM3A_MOUSE      EDPWNCYMCGHKGTYGLLRRREDWPSRLQMFFANNHDQEFDPPKV----YPPVPAEKRKP 629
                           *.   *  **    *      .. *  * *   . : :: .* ::     *  ** :  *

sp|Q08369|GATA4_MOUSE      -----PSSGASSGNSSNATS----SSSSSEEMRPIKTEPGLSSHYGHSSSMSQTFSTVSG 390
sp|O88508|DNM3A_MOUSE      IRVLSLFDGIATGLLVLKDLGIQVDRYIASEVCEDSITVGMVRHQGKIM----------- 678
                                  .* ::*           .   :.*:   .   *:  * *:

sp|Q08369|GATA4_MOUSE      HGPSIHPVLSALKLSPQGYASPVTQTSQASSKQDSWNSLVLAD--SH------------- 435
sp|O88508|DNM3A_MOUSE      ------------------YVGDVRS--VTQKHIQEWGPFDLVIGGSPCNDLSIVNPARKG 718
                                             *.. * .   :..: :.*. : *.   *

sp|Q08369|GATA4_MOUSE      -----GDIITA------------------------------------------------- 441
sp|O88508|DNM3A_MOUSE      LYEGTGRLFFEFYRLLHDARPKEGDDRPFFWLFENVVAMGVSDKRDISRFLESNPVMIDA 778
                                * ::

sp|Q08369|GATA4_MOUSE      ------------------------------------------------------------ 441
sp|O88508|DNM3A_MOUSE      KEVSAAHRARYFWGNLPGMNRPLASTVNDKLELQECLEHGRIAKFSKVRTITTRSNSIKQ 838


sp|Q08369|GATA4_MOUSE      ------------------------------------------------------------ 441
sp|O88508|DNM3A_MOUSE      GKDQHFPVFMNEKEDILWCTEMERVFGFPVHYTDVSNMSRLARQRLLGRSWSVPVIRHLF 898


sp|Q08369|GATA4_MOUSE      ----------   441
sp|O88508|DNM3A_MOUSE      APLKEYFACV   908
```

2.Alignment of GATA4 and DNMT3A in Human

```
CLUSTAL O(1.2.4) multiple sequence alignment


sp|P43694|GATA4_HUMAN      ------------------------------------------------------------ 0
sp|Q9Y6K1|DNM3A_HUMAN      MPAMPSSGPGDTSSSAAEREEDRKDGEEQEEPRGKEERQEPSTTARKVGRPGRKRKHPPV 60


sp|P43694|GATA4_HUMAN      -----------MYQSLAMA---------------------ANHGPPPGAY---------- 18
sp|Q9Y6K1|DNM3A_HUMAN      ESGDTPKDPAVISKSPSMAQDSGASELLPNGDLEKRSEPQPEEGSPAGGQKGGAPAEGEG 120
                                      : :* :**                      :.* * *.

sp|P43694|GATA4_HUMAN      ------------------------------------------------------------ 18
sp|Q9Y6K1|DNM3A_HUMAN      AAETLPEASRAVENGCCTPKEGRGAPAEAGKEQKETNIESMKMEGSRGRLRGGLGWESSL 180


sp|P43694|GATA4_HUMAN      ------------------------------------------------EAGGPGAFMHGA 30
sp|Q9Y6K1|DNM3A_HUMAN      RQRPMPRLTFQAGDPYYISKRKRDEWLARWKREAEKKAKVIAGMNAVEENQGPGESQKVE 240
                                                                           *  ***   :

sp|P43694|GATA4_HUMAN      GAASSPVYVPTPRVPSS--VLGLSYLQGGGAGSASGGASGGSSGGAASGAGPGTQQGSPG 88
sp|Q9Y6K1|DNM3A_HUMAN      EA--SPPAVQQPTDPASPTVATTPEPVGSDAGDKNATKAGDDEPEYE--DGRGFGIGELV 296
                            *  **  *  *  *:*  *       *..**. ..  :*...       * *   *.

sp|P43694|GATA4_HUMAN      WSQ----------------AGAD----GAAYTP------------PPVSPRFSFPGTTG- 115
sp|Q9Y6K1|DNM3A_HUMAN      WGKLRGFSWWPGRIVSWWMTGRSRAAEGTRWVMWFGDGKFSVVCVEKLMPLSSFCSAFHQ 356
                           *.:                :* .    *: :.               : *  ** .:

sp|P43694|GATA4_HUMAN      -----------SLAAAAAAAAAREAAAYSSG----------------GGAAGAGLAGREQ 148
sp|Q9Y6K1|DNM3A_HUMAN      ATYNKQPMYRKAIYEVLQVASSRAGKLFPVCHDSDESDTAKAVEVQNKPMIEWALGGFQP 416
                                      ::  .  .*::* .  :                         .*.* :

sp|P43694|GATA4_HUMAN      YGRAGFAG--SYSSPYPAYMADVGASWAAAAAASAGPFDSPVLHSLPGRANPAARHPNL- 205
sp|Q9Y6K1|DNM3A_HUMAN      SGPKGLEPPEEEKNPYKEV---YTDMWVEPEAAAYAP---PPPAKKPRKS--TAEKPKVK 468
                            *  *:    . ..**          *.   **: .*   *   . * ::  :*.:*::

sp|P43694|GATA4_HUMAN      DMFDD--------------FSEGRECVNCGAMSTPLW----------------------- 228
sp|Q9Y6K1|DNM3A_HUMAN      EIIDERTRERLVYEVRQKCRNIEDICISCGSLNVTLEHPLFVGGMCQNCKNCFLECAYQY 528
                           :::*:               .    *:.**::.. *

sp|P43694|GATA4_HUMAN      RRDGTGHYLCNACGLYHKMNGINRPLIKPQRRLSASRRVGLSCANCQTTTT---TLWRRN 285
sp|Q9Y6K1|DNM3A_HUMAN      DDDGYQSYCTICCGGREVLMCGN------------NNCCRCFCVECVDLLVGPGAAQAAI 576
                             **   *   .**  . :   *            ..     *.:*    .   :

sp|P43694|GATA4_HUMAN      AEGEPVCNACGL---YMKL---HGVPRPLAMRKEGIQTRKRKPKNLNKSKTPAAPSGSES 339
sp|Q9Y6K1|DNM3A_HUMAN      KEDPWNCYMCGHKGTYGLLRRREDWPSRLQMFFANNHDQEFDPP--------------KV 622
                            *.   *  **    *  *   .. *  * *   . : :: .*               :

sp|P43694|GATA4_HUMAN      LPPASGASSNSSNATTSSSEEMRPIKT---EPGLSS-------------HYGHSSSVSQT 383
sp|Q9Y6K1|DNM3A_HUMAN      YPPVPA-------------EKRKPIRVLSLFDGIATGLLVLKDLGIQVDRYIASEVCEDS 669
                            **. .             *: :**:.     *:::             :*  *.  .::

sp|P43694|GATA4_HUMAN      FSVSAMSGHGPSIHPVLSALKLSPQGYASPVSQSPQTSSKQDSWNSLVLADS-------- 435
sp|Q9Y6K1|DNM3A_HUMAN      ITVGMV-RHQGKIM------------YVGDVR--SVTQKHIQEWGPFDLVIGGSPCNDLS 714
                           ::*. :  *  .*             *.. *     *..: :.*. : *. .

sp|P43694|GATA4_HUMAN      ------------HGDIITA----------------------------------------- 442
sp|Q9Y6K1|DNM3A_HUMAN      IVNPARKGLYEGTGRLFFEFYRLLHDARPKEGDDRPFFWLFENVVAMGVSDKRDISRFLE 774
                                        * ::

sp|P43694|GATA4_HUMAN      ------------------------------------------------------------ 442
sp|Q9Y6K1|DNM3A_HUMAN      SNPVMIDAKEVSAAHRARYFWGNLPGMNRPLASTVNDKLELQECLEHGRIAKFSKVRTIT 834


sp|P43694|GATA4_HUMAN      ------------------------------------------------------------ 442
sp|Q9Y6K1|DNM3A_HUMAN      TRSNSIKQGKDQHFPVFMNEKEDILWCTEMERVFGFPVHYTDVSNMSRLARQRLLGRSWS 894


sp|P43694|GATA4_HUMAN      ------------------   442
sp|Q9Y6K1|DNM3A_HUMAN      VPVIRHLFAPLKEYFACV   912
```

“*” indicating a single and fully conserved residue, “:” indicating residue with very similar properties, “.” indicating residue that is weakly similar.

SI7_Predicting the binding sites of C/EBPβ in *Cebpa* or *Pparg* promoter

1.*Cebpa*

Matrix ID	Name	Score	Relative score	Sequence ID	Start	End	Strand	Predicted sequence
MA0466.2	MA0466.2.CEBPB	11.653339	0.942632427917801		1815	1824	-	GTGGCGCAAC
MA0466.2	MA0466.2.CEBPB	9.875581	0.9185619940556174		1815	1824	+	GTTGCGCCAC
MA0466.1	MA0466.1.CEBPB	8.798325	0.9121646304288258		1814	1824	+	CGTTGCGCCAC
MA0466.1	MA0466.1.CEBPB	7.8923893	0.9026684892883058		66	76	+	GCTTGCATAAG
MA0466.2	MA0466.2.CEBPB	7.736664	0.8896015606791997		67	76	-	CTTATGCAAG
MA0466.2	MA0466.2.CEBPB	6.964209	0.8791427049521621		396	405	-	ATGGCTCAAC
MA0466.3	MA0466.3.CEBPB	6.5319104	0.850232371469726		1814	1825	+	CGTTGCGCCACG
MA0466.3	MA0466.3.CEBPB	6.5052366	0.849931305519567		1814	1825	-	CGTGGCGCAACG
MA0466.2	MA0466.2.CEBPB	5.11655	0.8541258345662862		67	76	+	CTTGCATAAG

2.*Pparg*

Matrix ID	Name	Score	Relative score	Sequence ID	Start	End	Strand	Predicted sequence
MA0466.2	MA0466.2.CEBPB	9.804644	0.9176015216158963		330	339	+	ATGACACAAT
MA0466.2	MA0466.2.CEBPB	6.3889585	0.8713539465960166		338	347	-	TTTACAAAAT
MA0466.2	MA0466.2.CEBPB	5.7441745	0.8626237230387835		1088	1097	-	CTTGCTCAAG
MA0466.1	MA0466.1.CEBPB	5.6612563	0.8792814394473567		1088	1098	-	TCTTGCTCAAG
MA0466.2	MA0466.2.CEBPB	5.4630036	0.8588167358383406		1088	1097	+	CTTGAGCAAG
MA0466.2	MA0466.2.CEBPB	5.0967236	0.8538573896019048		433	442	+	CTGACACAAG

SI8_Supplementary table of DEGs (GSE34303)

	ID	adj.P.Val	P.Value	t	B	Log2FC	Gene.syml	Gene.title
1	1865	5.41E-05	2.10E-09	-15.1	11.329	-1.29	RGS7	regulator of G-protein signaling 7
2	35994	5.41E-05	2.49E-09	-14.9	11.1983	-1.54	MYOZ2	myozenin 2
3	7177	5.41E-05	5.04E-09	-14.1	10.6395	-0.86		
4	33494	5.41E-05	5.25E-09	-14	10.6071	-1.07	HDAC9	histone deacetylase 9
5	12579	5.41E-05	6.65E-09	-13.7	10.4156	-0.938	KRT18	keratin 18
6	4991	5.41E-05	7.21E-09	-13.6	10.35	-1.26	SCN9A	sodium voltage-gated channel alpha subunit 9
7	9431	6.05E-05	9.42E-09	-13.3	10.1323	-0.952		
8	39045	6.57E-05	1.22E-08	-13	9.9194	-1.05		
9	1543	6.57E-05	1.43E-08	-12.9	9.78572	-0.967	KRT18	keratin 18
10	1300	6.57E-05	1.46E-08	-12.9	9.76985	-0.944		
11	44854	6.76E-05	1.79E-08	-12.6	9.59723	-0.825		
12	6876	6.76E-05	2.47E-08	-12.3	9.32695	-0.838	KRT18	keratin 18
13	13270	6.76E-05	2.56E-08	-12.2	9.29614	-0.997		
14	9612	6.76E-05	3.08E-08	-12	9.13823	-0.99		
15	43233	6.76E-05	3.16E-08	-12	9.11561	-1.08		
16	10236	6.76E-05	3.20E-08	-12	9.10487	-0.851		
17	14951	6.76E-05	3.48E-08	-11.9	9.03438	-0.956		
18	33506	6.76E-05	3.61E-08	-11.9	9.00181	-1.02		
19	27799	6.76E-05	3.69E-08	-11.9	8.98347	-0.853		
20	32453	6.76E-05	3.72E-08	-11.9	8.97626	-1.07		
21	36248	6.76E-05	3.83E-08	-11.8	8.95182	-1.05		
22	2004	6.76E-05	4.12E-08	-11.7	8.88721	-1		
23	21911	6.76E-05	4.20E-08	-11.7	8.87063	-0.778	NTN4	netrin 4
24	22443	6.76E-05	4.30E-08	-11.7	8.85106	-1.09		
25	12388	6.76E-05	4.37E-08	-11.7	8.83781	-0.845		
26	42230	6.76E-05	4.41E-08	-11.7	8.82945	-0.91		
27	11068	6.76E-05	4.51E-08	-11.7	8.81021	-0.991		
28	652	6.76E-05	4.61E-08	-11.6	8.79138	-0.972		
29	3152	6.76E-05	4.65E-08	-11.6	8.78286	-1.08		
30	962	6.76E-05	4.66E-08	-11.6	8.78119	-1.09		
31	13566	6.76E-05	4.70E-08	11.6	8.77506	1	PLBD1	phospholipase B domain containing 1
32	44894	6.76E-05	4.90E-08	-11.6	8.73786	-1		
33	7910	6.76E-05	5.09E-08	-11.5	8.70451	-1.03		
34	37534	6.76E-05	5.15E-08	-11.5	8.69542	-0.871	GSG1	germ cell associated 1
35	19381	6.76E-05	5.42E-08	-11.5	8.65031	-0.887		
36	12862	6.76E-05	5.57E-08	-11.4	8.62723	-1.08		
37	30623	6.76E-05	5.60E-08	-11.4	8.62153	-0.939		
38	36933	6.76E-05	5.98E-08	-11.4	8.565	-0.688		
39	10934	6.76E-05	6.07E-08	-11.4	8.55249	-0.77		
40	30903	6.76E-05	6.13E-08	-11.3	8.54358	-0.953		
41	29205	6.76E-05	6.16E-08	-11.3	8.53923	-1.49	SCN9A	sodium voltage-gated channel alpha subunit 9
42	1482	6.79E-05	6.41E-08	-11.3	8.50374	-1.01		
43	35051	6.79E-05	6.49E-08	-11.3	8.49355	-1.07		
44	41172	7.74E-05	7.57E-08	-11.1	8.35872	-0.869		
45	38806	8.41E-05	8.41E-08	-11	8.26605	-0.972		
46	2461	8.81E-05	9.00E-08	-11	8.20566	-1.01		

47	19480	8.91E-05	9.30E-08	-10.9	8.17667	-1.02		
48	2778	0.00011	1.19E-07	-10.7	7.96174	-0.844		
49	4835	0.00012	1.33E-07	-10.6	7.85694	-0.984		
50	23587	0.00012	1.35E-07	-10.6	7.84724	-0.867		
51	24721	0.00012	1.42E-07	-10.5	7.80248	-0.869		
52	16421	0.00013	1.50E-07	10.5	7.7513	1.15	KLF8	Kruppel like factor 8
53	3039	0.00019	2.31E-07	-10.1	7.36342	-1.61	SCN9A	sodium voltage-gated channel alpha subunit 9
54	23353	0.00023	2.83E-07	9.91	7.17992	0.999	EFHC2	EF-hand domain containing 2
55	11650	0.00025	3.10E-07	-9.83	7.0973	-0.61		
56	29623	0.00027	3.41E-07	-9.75	7.00983	-0.887		
57	17405	0.00029	3.82E-07	-9.65	6.90578	-0.632		
58	16488	0.00029	3.86E-07	-9.64	6.8982	-0.946		
59	10754	0.00046	6.08E-07	-9.25	6.47954	-1.04	KRT18	keratin 18
60	41797	5.00E-04	6.89E-07	9.15	6.36484	0.726	ANOS1	anosmin 1
61	24033	0.00052	7.40E-07	9.09	6.29801	0.854	EFHC2	EF-hand domain containing 2
62	31449	0.00052	7.40E-07	-9.09	6.298	-0.528	CCND1	cyclin D1
63	16624	0.00055	7.99E-07	-9.02	6.22661	-0.718	FZD4	frizzled class receptor 4
64	4103	0.00055	8.05E-07	-9.02	6.22067	-1.77		
65	1075	0.00059	8.72E-07	8.95	6.14593	1.21	CRISPLD1	cysteine rich secretory protein LCCL domain containing 1
66	37038	0.00059	8.95E-07	-8.93	6.12137	-0.819		
67	10671	0.00062	9.50E-07	-8.88	6.06624	-1.27		
68	16475	0.00066	1.04E-06	8.81	5.98158	0.519	MDFI	MyoD family inhibitor
69	28552	0.00066	1.05E-06	-8.8	5.96933	-0.796		
70	23391	0.00079	1.29E-06	-8.64	5.78322	-0.936	FOLR3	folate receptor 3
71	16239	0.00084	1.38E-06	8.58	5.71665	0.828	GCA	grancalcin
72	42199	0.00086	1.44E-06	8.55	5.67518	0.924	WISP1	WNT1 inducible signaling pathway protein 1
73	25557	0.00093	1.61E-06	8.46	5.57473	0.909	IRAK3	interleukin 1 receptor associated kinase 3
74	5021	0.00094	1.65E-06	8.44	5.55009	0.63	HLA-DPB	major histocompatibility complex, class II, DP beta 1
75	39672	0.00098	1.75E-06	-8.4	5.49629	-0.787		
76	32276	0.00099	1.81E-06	8.37	5.46378	1.36	NUDT10	nudix hydrolase 10
77	16833	0.00099	1.81E-06	-8.37	5.46301	-0.544	CCND1	cyclin D1
78	10700	0.001	1.84E-06	-8.35	5.44518	-0.918		
79	18518	0.00102	1.91E-06	-8.33	5.41352	-0.71	HIST2H2A	histone cluster 2, H2aa4
80	29198	0.00102	1.94E-06	-8.32	5.39948	-0.596	HIST2H2A	histone cluster 2, H2aa4
81	39023	0.00102	1.95E-06	-8.31	5.39466	-0.564	C15orf52	chromosome 15 open reading frame 52
82	28371	0.00106	2.05E-06	-8.27	5.34334	-0.624	LOC1005	uncharacterized LOC100507487
83	28741	0.00106	2.06E-06	-8.27	5.33944	-0.538	HIST1H2B	histone cluster 1, H2bk
84	24200	0.00109	2.17E-06	8.23	5.29157	0.994	WISP1	WNT1 inducible signaling pathway protein 1
85	23893	0.00113	2.34E-06	-8.17	5.22151	-0.793	RDH10	retinol dehydrogenase 10 (all-trans)
86	17691	0.00115	2.41E-06	8.15	5.19086	1.03	SLC1A3	solute carrier family 1 member 3
87	27499	0.00115	2.43E-06	-8.14	5.18356	-0.523	CCND1	cyclin D1
88	34835	0.00115	2.49E-06	-8.12	5.16241	-0.623	CEACAM3	carcinoembryonic antigen related cell adhesion molecule 3
89	6635	0.00117	2.56E-06	-8.1	5.13414	-0.537	CCND1	cyclin D1
90	42511	0.00117	2.56E-06	-8.1	5.13394	-0.506	CCND1	cyclin D1
91	23541	0.00117	2.64E-06	8.08	5.10708	0.713	DMC1	DNA meiotic recombinase 1
92	27660	0.00117	2.66E-06	8.07	5.09898	0.906	IRAK3	interleukin 1 receptor associated kinase 3
93	34146	0.00117	2.69E-06	-8.06	5.08906	-0.554		

94	35304	0.00118	2.74E-06	-8.05	5.07268	-0.81	SH3TC2	SH3 domain and tetratricopeptide repeats 2
95	16453	0.0012	2.80E-06	-8.03	5.05195	-0.535	CCND1	cyclin D1
96	2001	0.00121	2.90E-06	8.01	5.0157	0.566	CDKN2C	cyclin dependent kinase inhibitor 2C
97	35603	0.00121	2.91E-06	8	5.01489	0.597	LFNG	LFNG O-fucosylpeptide 3-beta-N-acetylglucosaminyltransferase
98	31973	0.00123	3.02E-06	7.98	4.97848	0.834	LEF1	lymphoid enhancer binding factor 1
99	9801	0.00123	3.10E-06	-7.96	4.95347	-0.554		
100	28771	0.00123	3.11E-06	-7.95	4.95059	-0.554		
101	29196	0.00124	3.16E-06	-7.94	4.93549	-0.839	RGS5	regulator of G-protein signaling 5
102	1985	0.00124	3.21E-06	7.93	4.92051	0.986	LSP1	lymphocyte-specific protein 1
103	3809	0.00124	3.22E-06	-7.93	4.91916	-0.533		
104	24354	0.00129	3.39E-06	7.89	4.86881	0.513	MNS1	meiosis specific nuclear structural 1
105	35069	0.0013	3.46E-06	-7.87	4.84936	-0.744	OXTR	oxytocin receptor
106	5529	0.0013	3.48E-06	-7.87	4.84484	-0.529	CCND1	cyclin D1
107	16384	0.00133	3.57E-06	7.85	4.81984	0.634	TNC	tenascin C
108	2936	0.00138	3.77E-06	-7.81	4.76846	-0.544		
109	11337	0.00141	3.87E-06	7.79	4.74208	0.582	TK1	thymidine kinase 1
110	25810	0.00142	3.96E-06	-7.77	4.72004	-0.557		
111	1605	0.00144	4.09E-06	-7.75	4.69119	-0.542	CCND1	cyclin D1
112	3012	0.00144	4.12E-06	-7.74	4.68317	-0.765	RDH10	retinol dehydrogenase 10 (all-trans)
113	4522	0.00144	4.15E-06	7.74	4.67656	0.601		
114	14267	0.00144	4.17E-06	7.74	4.67233	0.786	EYA1	EYA transcriptional coactivator and phosphatase 1
115	28800	0.00146	4.28E-06	7.72	4.6467	1.02	PRKAR2B	protein kinase cAMP-dependent type II regulatory subunit beta
116	44008	0.00147	4.35E-06	-7.7	4.6314	-0.932		
117	30942	0.00147	4.40E-06	-7.7	4.6216	-0.561		
118	44635	0.00147	4.43E-06	7.69	4.61514	0.632		
119	28565	0.00147	4.45E-06	-7.69	4.60975	-0.533	CCND1	cyclin D1
120	1247	0.00151	4.63E-06	-7.66	4.57277	-1.02	SDPR	serum deprivation response
121	3674	0.00158	4.94E-06	-7.61	4.50967	-0.548		
122	42741	0.00158	4.95E-06	-7.61	4.50795	-0.537	CCND1	cyclin D1
123	10389	0.00161	5.18E-06	7.58	4.46557	0.59	BIRC5	baculoviral IAP repeat containing 5
124	44651	0.00161	5.18E-06	-7.58	4.46555	-0.523	C8orf34	chromosome 8 open reading frame 34
125	33712	0.00161	5.24E-06	-7.57	4.45287	-0.547		
126	12384	0.00161	5.26E-06	7.56	4.44944	0.543	PRIM1	primase (DNA) subunit 1
127	3043	0.00163	5.39E-06	-7.55	4.42698	-0.726	SGIP1	SH3 domain GRB2 like endophilin interacting protein 1
128	28938	0.00163	5.43E-06	7.54	4.42	0.594	BIRC5	baculoviral IAP repeat containing 5
129	29399	0.00164	5.49E-06	7.53	4.40892	0.694	CYP7B1	cytochrome P450 family 7 subfamily B member 1
130	43966	0.00165	5.57E-06	7.52	4.39487	0.87	HLA-DPB1	major histocompatibility complex, class II, DP beta 1
131	20540	0.00179	6.41E-06	-7.42	4.26073	-0.559		
132	15797	0.00179	6.42E-06	7.42	4.25923	0.589	BIRC5	baculoviral IAP repeat containing 5
133	229	0.00182	6.58E-06	7.4	4.23513	0.89	KCNJ15	potassium voltage-gated channel subfamily J member 15
134	30653	0.00182	6.61E-06	7.4	4.23171	0.602	BIRC5	baculoviral IAP repeat containing 5
135	20584	0.00184	6.75E-06	-7.38	4.21152	-0.603	IL17D	interleukin 17D
136	41344	0.00187	6.99E-06	-7.36	4.17728	-0.948	EDN1	endothelin 1
137	14273	0.00187	6.99E-06	-7.36	4.17705	-0.557	HIST2H2B	histone cluster 2, H2be
138	29056	0.0019	7.31E-06	7.33	4.13465	0.614	BIRC5	baculoviral IAP repeat containing 5
139	34008	0.00191	7.38E-06	7.32	4.12594	0.588	BIRC5	baculoviral IAP repeat containing 5
140	16263	0.00192	7.47E-06	-7.31	4.11361	-0.959	EDN1	endothelin 1

141	11421	0.00192	7.53E-06	7.31	4.10587	0.506	KIF20A	kinesin family member 20A
142	17502	0.00192	7.66E-06	7.29	4.08896	0.713	MRO	maestro
143	3442	0.00192	7.71E-06	-7.29	4.08274	-0.965	EDN1	endothelin 1
144	802	0.00192	7.74E-06	-7.29	4.07929	-0.98	EDN1	endothelin 1
145	33234	0.00192	7.77E-06	7.28	4.07578	0.59	BIRC5	baculoviral IAP repeat containing 5
146	21277	0.00192	7.86E-06	7.28	4.06466	0.59	BIRC5	baculoviral IAP repeat containing 5
147	16566	0.00194	8.04E-06	7.26	4.04315	0.502		
148	5239	0.00196	8.23E-06	-7.24	4.02097	-0.953	EDN1	endothelin 1
149	21178	0.00196	8.25E-06	-7.24	4.01854	-0.659	USP53	ubiquitin specific peptidase 53
150	24624	0.00196	8.43E-06	7.23	3.99743	0.597	BUB1	BUB1 mitotic checkpoint serine/threonine kinase
151	38896	0.00196	8.46E-06	-7.22	3.99352	-0.833	GDF15	growth differentiation factor 15
152	2867	0.002	8.64E-06	7.21	3.97349	0.5	KIF20A	kinesin family member 20A
153	10725	0.00204	8.98E-06	-7.18	3.93712	-0.52	HIST1H1C	histone cluster 1, H1c
154	36067	0.00206	9.16E-06	7.17	3.91764	0.587	BIRC5	baculoviral IAP repeat containing 5
155	39605	0.00208	9.42E-06	-7.15	3.89008	-0.965	PLAT	plasminogen activator, tissue type
156	6799	0.00208	9.43E-06	7.15	3.88972	0.523	PRIM1	primase (DNA) subunit 1
157	43154	0.00208	9.43E-06	7.15	3.88935	0.771		
158	6554	0.00208	9.50E-06	7.14	3.8826	0.908	SLC1A3	solute carrier family 1 member 3
159	23695	0.00208	9.67E-06	-7.13	3.8651	-0.948	EDN1	endothelin 1
160	13083	0.00209	9.79E-06	-7.12	3.85296	-1.17	ARHGAP2	Rho GTPase activating protein 29
161	18972	0.00209	9.83E-06	7.12	3.84929	0.501	KIF20A	kinesin family member 20A
162	3547	0.00209	9.94E-06	-7.11	3.83908	-0.933	PLAT	plasminogen activator, tissue type
163	16103	0.00209	9.99E-06	-7.11	3.83404	-0.958	EDN1	endothelin 1
164	35141	0.00209	1.00E-05	-7.11	3.83117	-0.952		
165	39984	0.00209	1.00E-05	-7.1	3.82867	-0.617	ATP6V0A4	ATPase H+ transporting V0 subunit a4
166	39475	0.00213	1.03E-05	-7.08	3.80246	-0.952	PLAT	plasminogen activator, tissue type
167	19059	0.00215	1.05E-05	-7.08	3.78986	-0.963	EDN1	endothelin 1
168	31326	0.00217	1.07E-05	7.06	3.7674	0.606	BRIP1	BRCA1 interacting protein C-terminal helicase 1
169	16086	0.00217	1.09E-05	-7.05	3.75292	-0.966	EDN1	endothelin 1
170	11647	0.00217	1.09E-05	-7.05	3.751	-0.95	EDN1	endothelin 1
171	17452	0.00217	1.09E-05	-7.05	3.74859	-0.944		
172	5886	0.00217	1.10E-05	-7.04	3.73947	-0.957	PLAT	plasminogen activator, tissue type
173	14672	0.00217	1.11E-05	-7.04	3.73506	-0.959	PLAT	plasminogen activator, tissue type
174	1641	0.00219	1.13E-05	-7.02	3.71885	-0.668	PSTPIP2	proline-serine-threonine phosphatase interacting protein 2
175	6620	0.00219	1.13E-05	-7.02	3.7177	-0.938	PLAT	plasminogen activator, tissue type
176	12189	0.00219	1.14E-05	7.01	3.70262	0.593	BIRC5	baculoviral IAP repeat containing 5
177	31116	0.00219	1.15E-05	-7.01	3.69844	-0.967	PLAT	plasminogen activator, tissue type
178	33474	0.00219	1.17E-05	-7	3.68144	-0.669	PAPPA	pappalysin 1
179	14469	0.0022	1.18E-05	-6.99	3.67258	-0.955	PLAT	plasminogen activator, tissue type
180	42676	0.0022	1.18E-05	6.99	3.67072	0.505	KIF20A	kinesin family member 20A
181	12667	0.00223	1.21E-05	6.97	3.64859	0.503	KIF20A	kinesin family member 20A
182	35826	0.00227	1.24E-05	-6.96	3.6283	-0.969	PLAT	plasminogen activator, tissue type
183	38632	0.00228	1.24E-05	6.95	3.62148	1.07	SYNDIG1	synapse differentiation inducing 1
184	14074	0.00231	1.29E-05	6.93	3.58943	0.848	E2F8	E2F transcription factor 8
185	13115	0.00231	1.29E-05	6.93	3.5854	0.508	KIF20A	kinesin family member 20A
186	25039	0.00231	1.29E-05	-6.93	3.5839	-0.715	RGS5	regulator of G-protein signaling 5
187	10068	0.00231	1.30E-05	6.93	3.58114	0.591	SPC24	SPC24, NDC80 kinetochore complex component

188	10448	0.00232	1.32E-05	6.92	3.56843	0.503	DEPDC1	DEP domain containing 1
189	30495	0.00232	1.32E-05	6.92	3.56733	0.547	PRIM1	primase (DNA) subunit 1
190	3246	0.00232	1.33E-05	-6.91	3.5581	-0.684	USP53	ubiquitin specific peptidase 53
191	11206	0.00232	1.34E-05	-6.91	3.5524	-0.7	KCTD16	potassium channel tetramerization domain containing 16
192	24111	0.00233	1.36E-05	6.9	3.53888	0.828	SLC25A27	solute carrier family 25 member 27
193	23213	0.00233	1.36E-05	-6.89	3.53756	-0.952		
194	20760	0.0024	1.41E-05	6.87	3.50254	0.79	BACH2	BTB domain and CNC homolog 2
195	44429	0.00241	1.42E-05	6.86	3.49252	0.874	CHST6	carbohydrate sulfotransferase 6
196	1612	0.00243	1.45E-05	-6.85	3.47559	-0.8	PGBD3///I	piggyBac transposable element derived 3///ERCC excision repair 6, chromatin remodeling factor
197	15025	0.00243	1.45E-05	6.85	3.47488	0.88	LRRC6	leucine rich repeat containing 6
198	30641	0.00247	1.49E-05	-6.83	3.4482	-0.729	SH3TC2	SH3 domain and tetratricopeptide repeats 2
199	33203	0.00248	1.50E-05	6.83	3.44386	0.513	PRIM1	primase (DNA) subunit 1
200	20651	0.0025	1.53E-05	-6.81	3.41976	-0.737	KRT16	keratin 16
201	41345	0.00251	1.56E-05	6.8	3.40586	0.965	BAALC	brain and acute leukemia, cytoplasmic
202	9442	0.00251	1.56E-05	6.8	3.4048	0.548	PRIM1	primase (DNA) subunit 1
203	1173	0.00252	1.56E-05	6.8	3.40044	1.34	CH25H	cholesterol 25-hydroxylase
204	16004	0.00252	1.58E-05	6.79	3.38909	0.926	WISP1	WNT1 inducible signaling pathway protein 1
205	24039	0.00252	1.58E-05	6.79	3.38866	0.532	PRIM1	primase (DNA) subunit 1
206	26940	0.00252	1.60E-05	-6.78	3.38001	-0.993		
207	10011	0.00257	1.65E-05	-6.76	3.34983	-0.555	HECW2	HECT, C2 and WW domain containing E3 ubiquitin protein ligase 2
208	22838	0.00259	1.69E-05	6.75	3.3279	0.73		
209	30658	0.00259	1.69E-05	-6.75	3.32764	-0.725		
210	39692	0.00264	1.74E-05	6.73	3.29941	0.605	KIF15	kinesin family member 15
211	10028	0.00264	1.74E-05	6.73	3.2989	1.06	ARHGAP2	Rho GTPase activating protein 28
212	11229	0.00266	1.77E-05	6.72	3.28189	0.658	NRG1	neuregulin 1
213	36686	0.0027	1.80E-05	-6.7	3.26581	-0.962	PLAT	plasminogen activator, tissue type
214	17922	0.00271	1.81E-05	6.7	3.25743	0.502	PRIM1	primase (DNA) subunit 1
215	10012	0.00273	1.83E-05	-6.69	3.24662	-0.613	GALNT5	polypeptide N-acetylgalactosaminyltransferase 5
216	13155	0.00279	1.88E-05	-6.67	3.22345	-0.616	BMP6	bone morphogenetic protein 6
217	28683	0.00285	1.93E-05	6.66	3.19667	0.673	CENPF	centromere protein F
218	40964	0.00285	1.93E-05	-6.66	3.19552	-0.586		
219	12747	0.00286	1.94E-05	6.65	3.1893	0.586	SKA3	spindle and kinetochore associated complex subunit 3
220	6155	0.00288	1.96E-05	6.65	3.18082	1.1		
221	23754	0.00288	1.97E-05	6.64	3.17714	0.64	MIR924H(	MIR924 host gene
222	43482	0.00291	2.07E-05	6.61	3.12813	0.536	CHST1	carbohydrate sulfotransferase 1
223	16543	0.00297	2.14E-05	6.59	3.09588	0.807	KNL1	kinetochore scaffold 1
224	38259	0.00301	2.19E-05	6.57	3.07554	0.605	PLCG2	phospholipase C gamma 2
225	44463	0.00301	2.19E-05	-6.57	3.07209	-1	LINC0102	long intergenic non-protein coding RNA 1021
226	16646	0.00304	2.24E-05	-6.56	3.05264	-0.9	SGIP1	SH3 domain GRB2 like endophilin interacting protein 1
227	33248	0.00304	2.24E-05	6.56	3.05106	1.02	GRIA1	glutamate ionotropic receptor AMPA type subunit 1
228	39088	0.00306	2.27E-05	6.55	3.04048	0.512	NDC80	NDC80, kinetochore complex component
229	25400	0.00306	2.29E-05	6.54	3.03021	1.38	DDIT4L	DNA damage inducible transcript 4 like
230	28830	0.00306	2.29E-05	6.54	3.02977	0.524	PRIM1	primase (DNA) subunit 1
231	32544	0.00306	2.30E-05	6.54	3.02501	0.553		
232	16755	0.00306	2.31E-05	6.54	3.02345	0.631	CENPA	centromere protein A
233	38108	0.00306	2.32E-05	6.53	3.01671	0.633	HIST1H1A	histone cluster 1, H1a
234	11483	0.00306	2.32E-05	6.53	3.01608	0.77	BRCA2	BRCA2, DNA repair associated

235	43025	0.00312	2.39E-05	-6.51	2.98839	-1.03	FGL2	fibrinogen like 2
236	1774	0.00313	2.41E-05	-6.51	2.98136	-0.611	MAP3K7C	MAP3K7 C-terminal like
237	32847	0.0032	2.50E-05	6.48	2.94469	0.72	FAM111B	family with sequence similarity 111 member B
238	41394	0.0032	2.51E-05	6.48	2.94038	0.744	TMSB15A	thymosin beta 15a
239	43572	0.0032	2.52E-05	6.48	2.93564	0.752	CDCA7	cell division cycle associated 7
240	13501	0.0032	2.55E-05	6.47	2.92426	0.528	DTL	denticleless E3 ubiquitin protein ligase homolog
241	33136	0.0032	2.56E-05	6.47	2.92228	0.686	ARHGAP4	Rho GTPase activating protein 44
242	21855	0.0032	2.56E-05	6.47	2.92204	0.559	RRM2	ribonucleotide reductase regulatory subunit M2
243	1512	0.0032	2.58E-05	6.46	2.91471	0.516	PRIM1	primase (DNA) subunit 1
244	4651	0.0032	2.58E-05	6.46	2.91421	1.03	HTRA3	HtrA serine peptidase 3
245	9288	0.00325	2.64E-05	6.45	2.89154	0.913	CKM	creatine kinase, M-type
246	11208	0.00326	2.67E-05	-6.44	2.88289	-1.15		
247	44552	0.00326	2.67E-05	6.44	2.88248	0.579	EZH2	enhancer of zeste 2 polycomb repressive complex 2 subunit
248	40030	0.00326	2.67E-05	-6.44	2.88118	-0.694		
249	26345	0.00329	2.72E-05	-6.43	2.86237	-0.908		
250	8432	0.00337	2.83E-05	6.4	2.82612	0.54	NEK2	NIMA related kinase 2
251	40136	0.00337	2.85E-05	6.4	2.81832	0.504	NCAPH	non-SMC condensin I complex subunit H
252	8796	0.00337	2.86E-05	-6.4	2.81468	-0.678	CFH	complement factor H
253	38205	0.00337	2.87E-05	6.39	2.81031	0.511	CCNB2	cyclin B2
254	22608	0.00337	2.87E-05	6.39	2.81023	0.636	WDHD1	WD repeat and HMG-box DNA binding protein 1
255	38270	0.00338	2.90E-05	-6.39	2.80045	-1.12	DAW1	dynein assembly factor with WD repeats 1
256	30419	0.0034	2.92E-05	6.38	2.79324	0.517	PRIM1	primase (DNA) subunit 1
257	30872	0.0034	2.94E-05	-6.38	2.7869	-0.707	LRRN3	leucine rich repeat neuronal 3
258	12389	0.0034	2.95E-05	6.38	2.7848	0.848	RBPMS2	RNA binding protein with multiple splicing 2
259	22006	0.00348	3.04E-05	6.36	2.75621	0.538	MT1F	metallothionein 1F
260	15180	0.0035	3.07E-05	6.35	2.7467	0.696	MARK1	microtubule affinity regulating kinase 1
261	12137	0.0035	3.07E-05	6.35	2.74519	0.604		
262	35929	0.00356	3.17E-05	-6.33	2.71305	-0.657	DMD	dystrophin
263	17098	0.00356	3.18E-05	-6.33	2.7115	-1		
264	6383	0.00356	3.18E-05	6.33	2.71035	0.951	MATN2	matrilin 2
265	43842	0.00362	3.24E-05	6.31	2.69119	0.561		
266	14812	0.00364	3.28E-05	-6.31	2.68057	-0.551	AP1S3	adaptor related protein complex 1 sigma 3 subunit
267	30883	0.00365	3.33E-05	6.3	2.66728	0.655	HMGB2	high mobility group box 2
268	16196	0.00369	3.38E-05	6.29	2.65223	0.563	RADIL	Rap associating with DIL domain
269	35885	0.00369	3.38E-05	-6.29	2.65186	-0.656	LINC0161!	long intergenic non-protein coding RNA 1615
270	32524	0.00371	3.41E-05	6.28	2.64345	0.596	KIAA0101	KIAA0101
271	10106	0.00378	3.50E-05	-6.26	2.61852	-0.552	CFHR3	complement factor H related 3
272	9436	0.00378	3.50E-05	6.26	2.618	0.551	DTL	denticleless E3 ubiquitin protein ligase homolog
273	34135	0.00383	3.58E-05	-6.25	2.59556	-0.522		
274	41304	0.00383	3.58E-05	-6.25	2.59479	-1.15	PSG1	pregnancy specific beta-1-glycoprotein 1
275	22798	0.00385	3.62E-05	6.24	2.58466	0.56	HMMR	hyaluronan mediated motility receptor
276	10512	0.00388	3.66E-05	6.23	2.57277	0.629		
277	6890	0.00388	3.68E-05	6.23	2.5672	0.682	CENPF	centromere protein F
278	41762	0.00389	3.71E-05	-6.23	2.56166	-1.33	F3	coagulation factor III, tissue factor
279	44362	0.00398	3.83E-05	-6.21	2.52964	-0.808		
280	16668	0.00399	3.86E-05	6.2	2.52187	0.504	FOXM1	forkhead box M1
281	22071	0.00399	3.87E-05	-6.2	2.51831	-0.644	KRT14	keratin 14

282	10813	0.00399	3.88E-05	6.2	2.51757	0.549	KNL1	kinetochore scaffold 1
283	16440	0.00403	3.97E-05	-6.18	2.49321	-1.04		
284	32342	0.00403	3.98E-05	-6.18	2.49063	-0.707	ZG16B	zymogen granule protein 16B
285	16625	0.00403	3.99E-05	6.18	2.48856	0.602	NDC80	NDC80, kinetochore complex component
286	40966	0.00403	4.00E-05	6.18	2.48751	0.511		
287	38855	0.00407	4.06E-05	-6.17	2.47323	-1.11	ARHGAP2	Rho GTPase activating protein 29
288	41900	0.00407	4.07E-05	-6.17	2.4711	-0.596	HECW2	HECT, C2 and WW domain containing E3 ubiquitin protein ligase 2
289	40691	0.00407	4.07E-05	-6.17	2.46889	-0.523	CCND1	cyclin D1
290	11772	0.00411	4.16E-05	6.15	2.44955	0.579	CDC45	cell division cycle 45
291	23438	0.00414	4.19E-05	-6.15	2.44181	-0.966	MBP	myelin basic protein
292	23059	0.00417	4.26E-05	-6.14	2.42433	-0.571	AP1S3	adaptor related protein complex 1 sigma 3 subunit
293	23878	0.00419	4.32E-05	6.13	2.41079	0.772	DNM3	dynamin 3
294	1145	0.00422	4.37E-05	6.12	2.40051	0.523	DTL	denticleless E3 ubiquitin protein ligase homolog
295	34213	0.00423	4.40E-05	6.12	2.3933	0.598	CXCL12	C-X-C motif chemokine ligand 12
296	7966	0.00433	4.52E-05	6.1	2.36649	0.539	DTL	denticleless E3 ubiquitin protein ligase homolog
297	34070	0.00439	4.59E-05	-6.09	2.35162	-0.576		
298	16818	0.0044	4.61E-05	-6.09	2.34714	-0.511	NEDD4L	neural precursor cell expressed, developmentally down-regulated 4-like, E3 ubiquitin protein ligase
299	3203	0.00454	4.79E-05	6.06	2.31129	0.608	BRCA2	BRCA2, DNA repair associated
300	29493	0.00457	4.84E-05	-6.06	2.30004	-1.06	FGD4	FYVE, RhoGEF and PH domain containing 4
301	11025	0.00457	4.85E-05	6.05	2.29828	0.551	CCNB1	cyclin B1
302	16730	0.00477	5.13E-05	6.02	2.24425	0.657	AR	androgen receptor
303	7074	0.00479	5.16E-05	6.02	2.23819	0.546	DLGAP5	DLG associated protein 5
304	14209	0.00481	5.21E-05	6.01	2.22796	0.557	ARHGEF39	Rho guanine nucleotide exchange factor 39
305	38552	0.00481	5.26E-05	6	2.21877	0.677		
306	43187	0.00481	5.27E-05	6	2.21671	0.614	NEURL1B	neuralized E3 ubiquitin protein ligase 1B
307	38594	0.00481	5.33E-05	-5.99	2.20544	-0.748	WDR66	WD repeat domain 66
308	29071	0.00481	5.33E-05	5.99	2.20528	0.523	CDCA5	cell division cycle associated 5
309	35711	0.00481	5.34E-05	5.99	2.20439	0.697		
310	21128	0.00481	5.36E-05	-5.99	2.19997	-0.771	PSG6	pregnancy specific beta-1-glycoprotein 6
311	6306	0.00482	5.39E-05	-5.99	2.19512	-0.676		
312	16020	0.00484	5.42E-05	5.98	2.19013	1.07	MXRA5	matrix remodeling associated 5
313	24582	0.00487	5.47E-05	5.98	2.18148	0.791	GUCY1B3	guanylate cyclase 1 soluble subunit beta
314	19765	0.00492	5.54E-05	-5.97	2.16915	-1.12	FGD4	FYVE, RhoGEF and PH domain containing 4
315	4534	0.00498	5.63E-05	5.96	2.15183	1.3		
316	10735	0.005	5.68E-05	5.95	2.14473	0.849	IL7	interleukin 7
317	32816	0.005	5.68E-05	-5.95	2.14372	-0.809	KCTD4	potassium channel tetramerization domain containing 4
318	32587	0.00501	5.70E-05	-5.95	2.14036	-0.59	KRT14	keratin 14
319	2531	0.00503	5.73E-05	5.95	2.13561	0.912	EPSTI1	epithelial stromal interaction 1 (breast)
320	5566	0.00514	5.93E-05	5.93	2.10151	0.514	CIT	citron rho-interacting serine/threonine kinase
321	7896	0.00517	6.00E-05	5.92	2.08992	0.824	COL10A1	collagen type X alpha 1 chain
322	38285	0.00517	6.01E-05	5.92	2.08862	0.586	FAM64A	family with sequence similarity 64 member A
323	35127	0.00518	6.06E-05	5.91	2.0811	0.598	MAP2K6	mitogen-activated protein kinase kinase 6
324	3778	0.0052	6.11E-05	5.91	2.07236	0.783	PCSK1	proprotein convertase subtilisin/kexin type 1
325	9527	0.00522	6.16E-05	5.9	2.06449	0.52	SMC4	structural maintenance of chromosomes 4
326	43277	0.00524	6.21E-05	5.9	2.05688	0.55	UHRF1	ubiquitin like with PHD and ring finger domains 1
327	22631	0.00526	6.25E-05	5.89	2.04998	0.502	OIP5	Opa interacting protein 5
328	15822	0.00531	6.37E-05	5.88	2.03099	0.538	DTL	denticleless E3 ubiquitin protein ligase homolog

329	37527	0.00533	6.41E-05	-5.88	2.02467	-0.58	PRSS12	protease, serine 12
330	10443	0.00543	6.61E-05	5.86	1.99507	0.531	DTL	denticleless E3 ubiquitin protein ligase homolog
331	42687	0.00543	6.62E-05	5.86	1.99376	0.669	BRCA2	BRCA2, DNA repair associated
332	10990	0.00543	6.64E-05	5.86	1.99089	0.529	DTL	denticleless E3 ubiquitin protein ligase homolog
333	30255	0.00543	6.65E-05	5.86	1.98901	0.573	CD40	CD40 molecule
334	29343	0.00551	6.81E-05	5.84	1.96659	0.633	ZNF257	zinc finger protein 257
335	9717	0.00557	6.93E-05	-5.83	1.94827	-1.41	SERPINB2	serpin family B member 2
336	3381	0.00562	7.01E-05	5.82	1.93746	0.543	CYB5R2	cytochrome b5 reductase 2
337	18409	0.00569	7.12E-05	5.81	1.92267	0.525	SLC9A9	solute carrier family 9 member A9
338	20390	0.0057	7.14E-05	5.81	1.91922	0.531	KIF2C	kinesin family member 2C
339	21075	0.00574	7.25E-05	5.8	1.90506	0.526	TOP2A	topoisomerase (DNA) II alpha
340	43398	0.00574	7.26E-05	-5.8	1.9036	-0.618	GPRC5A	G protein-coupled receptor class C group 5 member A
341	28786	0.00576	7.29E-05	-5.8	1.89927	-0.625	IFIT2	interferon induced protein with tetratricopeptide repeats 2
342	35942	0.00583	7.45E-05	-5.78	1.87814	-1.22	CD36	CD36 molecule
343	17840	0.00591	7.59E-05	5.77	1.86	0.586	FAM64A	family with sequence similarity 64 member A
344	22539	0.00591	7.61E-05	5.77	1.85696	0.589		
345	29168	0.00591	7.62E-05	5.77	1.85645	0.536		
346	10682	0.00591	7.63E-05	5.77	1.85407	0.603	ESCO2	establishment of sister chromatid cohesion N-acetyltransferase 2
347	40148	0.00593	7.69E-05	5.76	1.84704	0.752	BRCA2	BRCA2, DNA repair associated
348	14168	0.00593	7.69E-05	5.76	1.84674	1.05	GBP5	guanylate binding protein 5
349	41953	0.00595	7.74E-05	5.76	1.84113	0.949	KCNMB1	potassium calcium-activated channel subfamily M regulatory beta subunit 1
350	29457	0.006	7.82E-05	5.75	1.83042	0.525	DTL	denticleless E3 ubiquitin protein ligase homolog
351	8153	0.00601	7.85E-05	5.75	1.82605	0.539	KIF11	kinesin family member 11
352	2692	0.00604	7.94E-05	-5.75	1.81606	-0.574	LOC54147	uncharacterized LOC541472
353	31488	0.00607	8.01E-05	5.74	1.80656	0.529	DTL	denticleless E3 ubiquitin protein ligase homolog
354	23837	0.00607	8.07E-05	5.73	1.79953	0.739	MATN2	matrilin 2
355	22840	0.00608	8.14E-05	5.73	1.79083	0.958	FRAS1	Fraser extracellular matrix complex subunit 1
356	8882	0.00614	8.27E-05	5.72	1.77535	0.871		
357	35202	0.00626	8.52E-05	-5.7	1.74637	-0.697		
358	7921	0.00636	8.82E-05	-5.68	1.71276	-0.553	NEDD4L	neural precursor cell expressed, developmentally down-regulated 4-like, E3 ubiquitin protein ligase
359	39351	0.00636	8.83E-05	5.68	1.71108	0.506	NIM1K	NIM1 serine/threonine protein kinase
360	3846	0.0065	9.10E-05	5.66	1.68202	0.586	ASPM	abnormal spindle microtubule assembly
361	13380	0.0065	9.15E-05	5.66	1.6766	0.845	RUNX2	runt related transcription factor 2
362	28138	0.0065	9.15E-05	5.66	1.67638	0.594	FAM64A	family with sequence similarity 64 member A
363	26748	0.0065	9.19E-05	5.65	1.67175	0.67	TNC	tenascin C
364	27694	0.0065	9.21E-05	5.65	1.67002	0.696	FGF14-AS	FGF14 antisense RNA 2
365	19892	0.00659	9.38E-05	5.64	1.6521	0.591	FAM64A	family with sequence similarity 64 member A
366	10092	0.0066	9.41E-05	-5.64	1.64862	-0.917	PTPRR	protein tyrosine phosphatase, receptor type R
367	27518	0.00665	9.51E-05	5.63	1.63837	0.513	MMS22L	MMS22 like, DNA repair protein
368	15534	0.00666	9.55E-05	5.63	1.63468	0.582	FAM64A	family with sequence similarity 64 member A
369	27313	0.00667	9.58E-05	5.63	1.63125	0.593	FAM64A	family with sequence similarity 64 member A
370	8370	0.00667	9.61E-05	-5.63	1.62769	-0.522	TLR4	toll like receptor 4
371	41656	0.00667	9.63E-05	5.63	1.62623	0.507	KIF14	kinesin family member 14
372	14268	0.00667	9.64E-05	5.63	1.6252	0.603	CDCA8	cell division cycle associated 8
373	14242	0.00669	9.69E-05	-5.62	1.61938	-1.01	ANKRD1	ankyrin repeat domain 1
374	28920	0.00671	9.77E-05	5.62	1.61149	0.641	TP63	tumor protein p63
375	32512	0.00671	9.79E-05	5.62	1.60993	0.648	EPHX2	epoxide hydrolase 2

376	39794	0.0068	9.95E-05	5.61	1.59391	0.765	MAF	MAF bZIP transcription factor
377	36600	0.00683	0.0001	5.6	1.58155	0.536	METTL7A	methyltransferase like 7A
378	4401	0.00683	0.0001	-5.6	1.57916	-0.649	CFH	complement factor H
379	8793	0.00683	0.0001	-5.59	1.57304	-1.51	PCDH10	protocadherin 10
380	4672	0.00687	0.0001	5.59	1.56266	0.586	FAM64A	family with sequence similarity 64 member A
381	16932	0.00693	0.00011	5.58	1.5449	0.519	HOMER2	homer scaffolding protein 2
382	33827	0.007	0.00011	5.57	1.5309	0.706	ADAMTS9	ADAM metallopeptidase with thrombospondin type 1 motif 9
383	31924	0.007	0.00011	-5.57	1.52922	-0.5	TLR4	toll like receptor 4
384	29815	0.00709	0.00011	5.56	1.515	0.633	C4orf26	chromosome 4 open reading frame 26
385	2128	0.00709	0.00011	5.56	1.51288	0.525	NEK2	NIMA related kinase 2
386	15907	0.00713	0.00011	5.55	1.50404	0.513	ZWINT	ZW10 interacting kinetochore protein
387	8891	0.00718	0.00011	-5.54	1.49396	-1.09	PSG7	pregnancy specific beta-1-glycoprotein 7 (gene/pseudogene)
388	10014	0.00718	0.00011	5.54	1.49049	0.553		
389	26050	0.0072	0.00011	5.54	1.48455	0.617		
390	2188	0.00727	0.00011	5.53	1.4712	0.776	FGFR2	fibroblast growth factor receptor 2
391	289	0.00728	0.00011	5.53	1.46387	0.508	BRCA2	BRCA2, DNA repair associated
392	35523	0.00729	0.00011	5.52	1.4596	0.527	NRG2	neuregulin 2
393	6973	0.00729	0.00011	5.52	1.45718	0.58	FAM64A	family with sequence similarity 64 member A
394	20695	0.00729	0.00012	5.52	1.45575	0.581	FAM64A	family with sequence similarity 64 member A
395	28331	0.00729	0.00012	5.52	1.45524	0.576	CD40	CD40 molecule
396	5821	0.00734	0.00012	5.51	1.44659	0.537	CD40	CD40 molecule
397	18616	0.00738	0.00012	-5.51	1.43872	-0.719	GCKR	glucokinase (hexokinase 4) regulator
398	14163	0.00738	0.00012	5.51	1.43612	0.85	CDCA7	cell division cycle associated 7
399	10020	0.00738	0.00012	5.51	1.43336	0.729	BRCA2	BRCA2, DNA repair associated
400	17810	0.0074	0.00012	-5.5	1.42818	-0.502	TLR4	toll like receptor 4
401	879	0.00741	0.00012	5.5	1.42112	0.586	PLK4	polo like kinase 4
402	27260	0.00744	0.00012	5.49	1.40555	0.642	DBF4	DBF4 zinc finger
403	13923	0.00747	0.00012	5.48	1.3946	0.989	EPSTI1	epithelial stromal interaction 1 (breast)
404	33448	0.00756	0.00012	5.47	1.37602	0.621	MKI67	marker of proliferation Ki-67
405	27382	0.00757	0.00012	5.47	1.37383	0.506	WDR76	WD repeat domain 76
406	22985	0.00765	0.00013	-5.46	1.35612	-0.968	TMEM178	transmembrane protein 178B
407	33268	0.00765	0.00013	5.46	1.35378	0.554	NCAPG	non-SMC condensin I complex subunit G
408	19831	0.00766	0.00013	-5.46	1.35142	-0.788		
409	41309	0.00767	0.00013	-5.45	1.34916	-0.504	CDH1	cadherin 1
410	20121	0.00768	0.00013	5.45	1.34447	0.518	DTL	denticleless E3 ubiquitin protein ligase homolog
411	9953	0.00769	0.00013	-5.45	1.34107	-0.734	PSG8	pregnancy specific beta-1-glycoprotein 8
412	30426	0.00771	0.00013	5.45	1.33627	0.56	SPAG5	sperm associated antigen 5
413	27960	0.00773	0.00013	-5.44	1.3319	-0.633	M1AP	meiosis 1 associated protein
414	972	0.00773	0.00013	5.44	1.32819	0.599	SPC25	SPC25, NDC80 kinetochore complex component
415	43130	0.00774	0.00013	5.44	1.32268	0.514	POMC	proopiomelanocortin
416	15662	0.00783	0.00013	-5.43	1.30843	-1.2	FAM167A	family with sequence similarity 167 member A
417	31817	0.00783	0.00013	5.43	1.30521	0.528	PLK4	polo like kinase 4
418	4780	0.00783	0.00013	5.43	1.30457	0.859	HEYL	hes related family bHLH transcription factor with YRPW motif-like
419	25652	0.00787	0.00014	5.42	1.29531	0.581	FANCD2	Fanconi anemia complementation group D2
420	2855	0.00788	0.00014	-5.42	1.29184	-0.65	KCTD4	potassium channel tetramerization domain containing 4
421	42175	0.00801	0.00014	5.4	1.26497	1.21	KRBOX1	KRAB box domain containing 1
422	9013	0.00802	0.00014	-5.4	1.25982	-1.61	SERPINB2	serpin family B member 2

423	27754	0.00807	0.00014	5.4	1.25253	0.534	TTK	TTK protein kinase
424	37144	0.00818	0.00014	5.38	1.23301	0.578	FAM64A	family with sequence similarity 64 member A
425	42928	0.00818	0.00014	-5.38	1.23203	-0.743	TSC22D1-	TSC22D1 antisense RNA 1
426	1798	0.00824	0.00015	5.38	1.21968	0.601	HCAR1	hydroxycarboxylic acid receptor 1
427	20498	0.00824	0.00015	5.37	1.21782	0.659	4-Sep	septin 4
428	33091	0.00828	0.00015	5.37	1.20984	0.582	ANKRD65	ankyrin repeat domain 65
429	30470	0.00835	0.00015	-5.36	1.20016	-0.914		
430	37919	0.00836	0.00015	-5.36	1.19079	-0.916	FZD4	frizzled class receptor 4
431	19570	0.00847	0.00015	-5.35	1.17343	-0.629	TMEM200	transmembrane protein 200A
432	31064	0.0085	0.00015	5.34	1.16821	0.503		
433	21391	0.00862	0.00016	-5.33	1.15191	-0.521	STC2	stanniocalcin 2
434	13913	0.00866	0.00016	5.33	1.14328	0.6		
435	3795	0.00867	0.00016	5.33	1.13989	0.521	MCM10	minichromosome maintenance 10 replication initiation factor
436	39786	0.00869	0.00016	-5.33	1.13648	-0.987	OXTR	oxytocin receptor
437	42520	0.0088	0.00016	5.31	1.11907	0.655		
438	13971	0.00886	0.00016	-5.31	1.10833	-1.04	KCTD16	potassium channel tetramerization domain containing 16
439	30691	0.00887	0.00016	-5.31	1.10355	-1.07	CD36	CD36 molecule
440	29675	0.0091	0.00017	-5.29	1.07245	-0.764	PCDH10	protocadherin 10
441	40215	0.00917	0.00017	-5.28	1.05672	-0.655	BMPER	BMP binding endothelial regulator
442	37354	0.00922	0.00017	-5.27	1.04625	-0.687	NPAS1	neuronal PAS domain protein 1
443	28861	0.00922	0.00017	5.27	1.04404	0.657	B4GALT6	beta-1,4-galactosyltransferase 6
444	9410	0.00923	0.00017	5.27	1.04173	0.639	MBNL3	muscleblind like splicing regulator 3
445	5424	0.00923	0.00018	-5.27	1.03709	-0.713	LINC0111	long intergenic non-protein coding RNA 1111
446	7938	0.00928	0.00018	-5.26	1.02685	-0.657	CD34	CD34 molecule
447	40112	0.00928	0.00018	5.26	1.02525	0.568	BRCA2	BRCA2, DNA repair associated
448	27291	0.00928	0.00018	5.26	1.02483	0.501	BCL2	BCL2, apoptosis regulator
449	20791	0.00957	0.00019	5.22	0.96101	0.542	BRCA2	BRCA2, DNA repair associated
450	11168	0.00959	0.00019	-5.22	0.9575	-0.575		
451	28404	0.00962	0.00019	-5.21	0.95239	-0.962	C5orf46	chromosome 5 open reading frame 46
452	26732	0.00965	0.00019	-5.21	0.94464	-0.781	TMEFF2	transmembrane protein with EGF like and two follistatin like domains 2
453	8748	0.00965	0.00019	-5.21	0.94355	-0.853	TM4SF4	transmembrane 4 L six family member 4
454	25559	0.00987	0.0002	5.19	0.91455	0.515	STK24-AS	STK24 antisense RNA 1
455	35129	0.01005	0.0002	5.18	0.89205	0.794	AR	androgen receptor
456	30383	0.01015	0.00021	-5.17	0.87764	-1.15	LINC0101	long intergenic non-protein coding RNA 1013
457	19208	0.01016	0.00021	5.16	0.87057	0.846	MAF	MAF bZIP transcription factor
458	18901	0.01016	0.00021	5.16	0.86369	0.539	FAM83D	family with sequence similarity 83 member D
459	33419	0.01025	0.00021	5.15	0.84968	0.551	BCL2	BCL2, apoptosis regulator
460	4227	0.01028	0.00021	5.15	0.84588	0.572	CD40	CD40 molecule
461	41190	0.01043	0.00022	5.14	0.8284	0.546	CADPS2	calcium dependent secretion activator 2
462	14614	0.0105	0.00022	5.13	0.8202	1.32	ACKR3	atypical chemokine receptor 3
463	13781	0.0105	0.00022	5.13	0.81911	0.523	PRRT2	proline rich transmembrane protein 2
464	43681	0.01054	0.00022	-5.13	0.81457	-1.33	PCDH10	protocadherin 10
465	22589	0.01069	0.00023	-5.12	0.78973	-0.507		
466	34682	0.01076	0.00023	5.11	0.77427	0.549	BUB1B	BUB1 mitotic checkpoint serine/threonine kinase B
467	1471	0.01078	0.00023	5.11	0.77035	0.759		
468	21179	0.01078	0.00023	-5.1	0.76666	-0.807	RNF144B	ring finger protein 144B
469	18338	0.01089	0.00024	5.09	0.7499	0.532	GBP2	guanylate binding protein 2

470	14241	0.01107	0.00024	-5.08	0.72697	-1.14	PSG8	pregnancy specific beta-1-glycoprotein 8
471	35495	0.0111	0.00024	5.07	0.71767	0.518	ASPM	abnormal spindle microtubule assembly
472	23740	0.01122	0.00025	-5.06	0.69671	-0.504	TIMP3	TIMP metallopeptidase inhibitor 3
473	1451	0.01128	0.00025	-5.05	0.68228	-0.883	CYP26B1	cytochrome P450 family 26 subfamily B member 1
474	29549	0.01128	0.00025	5.05	0.68209	0.554	ZNF560	zinc finger protein 560
475	40478	0.01138	0.00026	-5.04	0.65934	-0.893		
476	8880	0.01156	0.00026	-5.03	0.63583	-0.976	PSG5	pregnancy specific beta-1-glycoprotein 5
477	3618	0.01162	0.00027	5.02	0.62659	0.542	ATAD5	ATPase family, AAA domain containing 5
478	12248	0.01171	0.00027	5.01	0.60699	1.24	UNC5C	unc-5 netrin receptor C
479	25597	0.01173	0.00027	5.01	0.60351	0.821	AR	androgen receptor
480	15703	0.01209	0.00028	4.98	0.56308	0.557	SHF	Src homology 2 domain containing F
481	12446	0.01237	0.00029	-4.96	0.53259	-0.74	DMD	dystrophin
482	39548	0.01237	0.00029	4.96	0.52869	0.502	KIF20B	kinesin family member 20B
483	40783	0.01237	0.0003	4.96	0.52522	0.516	MCM10	minichromosome maintenance 10 replication initiation factor
484	37026	0.01237	0.0003	-4.96	0.52473	-0.699	LYPD5	LY6/PLAUR domain containing 5
485	11423	0.01237	0.0003	4.96	0.5212	0.587	TRAF3IP2	TRAF3 interacting protein 2
486	33571	0.01237	0.0003	-4.96	0.51922	-0.529	CDH1	cadherin 1
487	27648	0.01237	0.0003	4.96	0.51871	0.765	MAF	MAF bZIP transcription factor
488	4178	0.01237	0.0003	4.96	0.51783	0.802	FAM20A	family with sequence similarity 20 member A
489	19335	0.01245	0.0003	-4.95	0.50944	-0.72	GPRC5A	G protein-coupled receptor class C group 5 member A
490	14988	0.01262	0.00031	4.94	0.48929	0.581	NEIL3	nei like DNA glycosylase 3
491	22534	0.01268	0.00031	4.94	0.48284	0.573	BCL2	BCL2, apoptosis regulator
492	36335	0.013	0.00032	4.91	0.44787	0.524	DEPDC1	DEP domain containing 1
493	12871	0.013	0.00032	4.91	0.44609	0.83	NAV2	neuron navigator 2
494	18260	0.013	0.00032	4.91	0.4456	0.541	SETBP1	SET binding protein 1
495	35940	0.01306	0.00032	4.91	0.43316	0.773	AR	androgen receptor
496	22687	0.01318	0.00033	-4.9	0.42025	-0.75		
497	14009	0.01318	0.00033	4.9	0.42016	0.539	FAM124A	family with sequence similarity 124 member A
498	36635	0.01321	0.00033	4.9	0.41598	0.89	FAM20A	family with sequence similarity 20 member A
499	3835	0.01347	0.00034	-4.88	0.38923	-0.847	PSG11	pregnancy specific beta-1-glycoprotein 11
500	16457	0.01369	0.00035	-4.86	0.3624	-0.947	TINAGL1	tubulointerstitial nephritis antigen like 1
501	14634	0.01395	0.00036	4.85	0.33811	0.764		
502	38852	0.01395	0.00036	4.85	0.33747	0.567	SKA1	spindle and kinetochore associated complex subunit 1
503	12732	0.01395	0.00036	-4.85	0.33586	-0.544	PRSS12	protease, serine 12
504	19216	0.01395	0.00036	4.85	0.33192	0.861	AR	androgen receptor
505	32258	0.01407	0.00036	4.84	0.31801	0.753	RBP1	retinol binding protein 1
506	43280	0.01407	0.00036	-4.84	0.31765	-0.723		
507	16435	0.01407	0.00036	4.84	0.31751	0.578	RFC3	replication factor C subunit 3
508	33261	0.01407	0.00036	4.84	0.31717	0.752	MAFB	MAF bZIP transcription factor B
509	8079	0.01407	0.00036	-4.84	0.31563	-0.935	PNMA2	paraneoplastic Ma antigen 2
510	31574	0.01418	0.00037	-4.83	0.30064	-0.99	SLC7A14	solute carrier family 7 member 14
511	32642	0.01418	0.00037	4.83	0.30021	0.628		
512	20031	0.01419	0.00037	4.83	0.29643	0.574	SETBP1	SET binding protein 1
513	40662	0.01419	0.00037	4.83	0.29505	0.643	BCL2	BCL2, apoptosis regulator
514	37867	0.01419	0.00037	-4.82	0.29339	-0.906	PRR15	proline rich 15
515	26626	0.01419	0.00037	4.82	0.29311	0.691	SALL1	spalt like transcription factor 1
516	36086	0.01419	0.00038	4.82	0.28792	0.698		

517	6678	0.01423	0.00038	4.82	0.28404	0.523		
518	2213	0.01437	0.00038	4.81	0.26904	0.515	CDK1	cyclin dependent kinase 1
519	19299	0.01471	0.0004	-4.79	0.23509	-0.775	ALDH1A3	aldehyde dehydrogenase 1 family member A3
520	753	0.01496	0.00041	4.77	0.20655	0.533		
521	33550	0.01496	0.00041	-4.77	0.20334	-0.812	STYK1	serine/threonine/tyrosine kinase 1
522	40277	0.01503	0.00041	4.77	0.19505	0.737	RIBC2	RIB43A domain with coiled-coils 2
523	29556	0.01503	0.00041	4.77	0.19478	0.584	ZNF367	zinc finger protein 367
524	23036	0.01522	0.00042	-4.76	0.17816	-0.767		
525	18866	0.01527	0.00042	4.75	0.17282	0.608	ZNF214	zinc finger protein 214
526	3325	0.01534	0.00042	4.75	0.16587	1.08		
527	20746	0.01548	0.00043	-4.74	0.15311	-0.5		
528	41715	0.01559	0.00043	-4.74	0.14482	-0.635	PSG9	pregnancy specific beta-1-glycoprotein 9
529	23835	0.01566	0.00044	4.73	0.13814	0.564	RPS6KA5	ribosomal protein S6 kinase A5
530	30285	0.01572	0.00044	4.73	0.1307	0.585	TRPC3	transient receptor potential cation channel subfamily C member 3
531	41989	0.01572	0.00044	4.73	0.12946	0.856	JAM2	junctional adhesion molecule 2
532	29652	0.01585	0.00045	4.72	0.11634	0.761	WNK3	WNK lysine deficient protein kinase 3
533	23450	0.01588	0.00045	-4.72	0.10451	-0.549	KCNJ16	potassium voltage-gated channel subfamily J member 16
534	33425	0.01591	0.00045	4.71	0.10132	0.511	GLI3	GLI family zinc finger 3
535	33179	0.01598	0.00046	4.71	0.09598	0.508		
536	16889	0.01608	0.00046	-4.7	0.08668	-0.566	TXNRD1	thioredoxin reductase 1
537	40880	0.01636	0.00047	-4.69	0.05699	-0.644	NLRP10	NLR family pyrin domain containing 10
538	30851	0.01651	0.00048	4.68	0.03962	0.551	ARHGAP1	Rho GTPase activating protein 11A
539	30680	0.01653	0.00048	4.68	0.03517	0.508	CDK1	cyclin dependent kinase 1
540	37268	0.01668	0.00049	4.67	0.02316	0.65	TGFB3	transforming growth factor beta 3
541	25690	0.01678	0.0005	4.66	0.01297	0.642	GALNT15	polypeptide N-acetylgalactosaminyltransferase 15
542	22748	0.01678	0.0005	-4.66	0.01173	-0.564	TLL2	tolloid like 2
543	6646	0.01689	0.0005	4.66	0.00011	0.508	CDK1	cyclin dependent kinase 1
544	38627	0.01699	0.00051	4.65	-0.0078	0.657	ZNF521	zinc finger protein 521
545	44121	0.01725	0.00052	-4.64	-0.0329	-0.649	CFAP70	cilia and flagella associated protein 70
546	30357	0.01725	0.00052	-4.64	-0.0338	-0.526		
547	42821	0.01741	0.00053	-4.62	-0.0535	-0.548	TNFRSF11	TNF receptor superfamily member 11a
548	21467	0.01763	0.00054	4.61	-0.0738	0.526	PBK	PDZ binding kinase
549	12438	0.01766	0.00054	-4.61	-0.0795	-0.904	PAGE5	PAGE family member 5
550	35654	0.01769	0.00055	4.61	-0.0845	0.769	AR	androgen receptor
551	24761	0.01776	0.00055	4.6	-0.0921	0.502	CDK1	cyclin dependent kinase 1
552	38968	0.01787	0.00056	4.59	-0.1063	0.813	CCDC102I	coiled-coil domain containing 102B
553	20013	0.01789	0.00056	4.59	-0.1097	1.07	PCSK1	proprotein convertase subtilisin/kexin type 1
554	41741	0.01789	0.00056	-4.59	-0.1099	-0.627	LINC0084:	long intergenic non-protein coding RNA 842
555	24589	0.01815	0.00058	4.58	-0.1351	0.631		
556	37635	0.01817	0.00058	4.58	-0.1376	0.606	ENPEP	glutamyl aminopeptidase
557	30523	0.01828	0.00059	-4.57	-0.1539	-0.882	LINC0101:	long intergenic non-protein coding RNA 1013
558	19935	0.01869	0.00061	4.55	-0.1897	0.597	MLIP	muscular LMNA-interacting protein
559	1682	0.01893	0.00063	4.53	-0.2192	0.531	CD40	CD40 molecule
560	44135	0.01896	0.00063	4.53	-0.2215	0.594		
561	22727	0.01902	0.00063	4.52	-0.2279	0.989	TMEM119	transmembrane protein 119
562	22689	0.01911	0.00064	4.52	-0.2354	0.811	DAPL1	death associated protein like 1
563	32992	0.01913	0.00064	4.52	-0.2375	0.502	CDK1	cyclin dependent kinase 1

564	44235	0.01929	0.00065	4.51	-0.2523	0.779	AR	androgen receptor
565	22885	0.01929	0.00065	4.51	-0.2528	0.783	AR	androgen receptor
566	15445	0.01933	0.00065	4.51	-0.2609	0.632	BCL2	BCL2, apoptosis regulator
567	22077	0.0194	0.00066	4.5	-0.2679	0.549	CD40	CD40 molecule
568	32002	0.0194	0.00066	4.5	-0.2701	0.691	IL17RD	interleukin 17 receptor D
569	9546	0.0194	0.00066	-4.5	-0.2701	-0.529	TLR4	toll like receptor 4
570	42227	0.01942	0.00066	4.5	-0.2722	0.632	MT1JP	metallothionein 1J, pseudogene
571	24458	0.01944	0.00066	4.5	-0.2744	0.82	AR	androgen receptor
572	30420	0.01945	0.00066	4.5	-0.2756	0.676	PARD6G	par-6 family cell polarity regulator gamma
573	15439	0.01959	0.00067	4.49	-0.2883	0.59	TMPO	thymopoietin
574	7964	0.01987	0.00069	4.48	-0.3125	0.584	BCL2	BCL2, apoptosis regulator
575	38919	0.01987	0.00069	-4.48	-0.3152	-0.932	DNER	delta/notch like EGF repeat containing
576	43517	0.01994	0.0007	4.47	-0.3216	0.506	CDK1	cyclin dependent kinase 1
577	28609	0.02	0.0007	4.47	-0.3264	0.774	ZBED6CL	ZBED6 C-terminal like
578	32215	0.0201	0.00071	4.46	-0.3355	0.848	FGFR2	fibroblast growth factor receptor 2
579	43582	0.0203	0.00072	4.45	-0.3532	0.552	CDK1	cyclin dependent kinase 1
580	33679	0.02053	0.00073	4.44	-0.3721	0.508	FZD3	frizzled class receptor 3
581	33503	0.02053	0.00073	4.44	-0.3723	0.728	AR	androgen receptor
582	31623	0.02061	0.00074	4.44	-0.3779	0.533	CDKN1C	cyclin dependent kinase inhibitor 1C
583	34604	0.02065	0.00074	4.44	-0.3815	0.528	CKAP2L	cytoskeleton associated protein 2 like
584	7991	0.02065	0.00074	-4.44	-0.382	-0.628	TEK	TEK receptor tyrosine kinase
585	38410	0.02065	0.00074	4.44	-0.3821	0.68		
586	28726	0.0207	0.00074	-4.44	-0.3857	-0.603	COBLL1	cordon-bleu WH2 repeat protein like 1
587	7962	0.02082	0.00075	-4.43	-0.3938	-0.583	CCDC190	coiled-coil domain containing 190
588	16295	0.02082	0.00075	4.43	-0.395	0.66	TGFB3	transforming growth factor beta 3
589	3057	0.02101	0.00076	4.42	-0.4109	0.612	ENOX1	ecto-NOX disulfide-thiol exchanger 1
590	11664	0.02102	0.00076	-4.42	-0.4123	-0.727		
591	12074	0.02104	0.00076	4.42	-0.414	0.681	BCL2	BCL2, apoptosis regulator
592	35617	0.02105	0.00077	-4.42	-0.4161	-0.535		
593	23320	0.02111	0.00077	-4.42	-0.4204	-1.08	SYT1	synaptotagmin 1
594	31646	0.02111	0.00077	4.41	-0.4214	0.71	DCSTAMP	dendrocyte expressed seven transmembrane protein
595	13412	0.02124	0.00078	4.41	-0.434	0.634	KNL1	kinetochore scaffold 1
596	13783	0.02152	0.0008	-4.4	-0.4542	-0.707	ARHGAP2	Rho GTPase activating protein 29
597	13383	0.02176	0.00081	4.39	-0.4725	0.654	BRCA2	BRCA2, DNA repair associated
598	42069	0.02178	0.00081	4.38	-0.4761	0.518		
599	28228	0.02191	0.00083	4.38	-0.4908	0.548	TGFB3	transforming growth factor beta 3
600	32316	0.02198	0.00083	-4.37	-0.4945	-0.916	LINC0113	long intergenic non-protein coding RNA 1133
601	31187	0.02204	0.00083	4.37	-0.499	0.545		
602	15581	0.02204	0.00083	4.37	-0.5003	0.604	GS1-24F4	uncharacterized LOC100652791
603	6286	0.02204	0.00083	-4.37	-0.5006	-0.631		
604	16417	0.02207	0.00084	4.37	-0.5027	0.566		
605	26844	0.0222	0.00085	4.36	-0.5177	0.566	RBL1	RB transcriptional corepressor like 1
606	8780	0.02232	0.00086	4.35	-0.5288	0.633	SMAD1	SMAD family member 1
607	21432	0.02245	0.00087	4.35	-0.5381	0.754	IGF1	insulin like growth factor 1
608	12967	0.02263	0.00088	-4.34	-0.5552	-0.67	CEMIP	cell migration inducing hyaluronan binding protein
609	44691	0.02285	0.00089	-4.33	-0.5688	-0.533		
610	18943	0.02308	0.00091	4.32	-0.5839	0.556	LOC44166	zinc finger protein 91 pseudogene

611	18333	0.02319	0.00091	4.32	-0.592	0.792	IGF1	insulin like growth factor 1
612	32492	0.02334	0.00093	4.31	-0.6068	0.506	IL17RD	interleukin 17 receptor D
613	1758	0.02338	0.00093	-4.31	-0.6134	-0.718	LRRN3	leucine rich repeat neuronal 3
614	23315	0.02339	0.00094	4.31	-0.6151	0.518	GIMAP2	GTPase, IMAP family member 2
615	2316	0.02354	0.00095	4.3	-0.6258	0.588	ZMAT4	zinc finger matrin-type 4
616	39160	0.02406	0.00098	4.28	-0.6635	0.519	LUM	lumican
617	42386	0.02422	0.00099	-4.27	-0.6726	-0.565	MECOM	MDS1 and EVI1 complex locus
618	8973	0.02427	0.001	4.27	-0.6763	0.79	IQCA1	IQ motif containing with AAA domain 1
619	4392	0.02455	0.00101	4.26	-0.691	0.599		
620	11094	0.02482	0.00103	-4.25	-0.7113	-0.649	PDE5A	phosphodiesterase 5A
621	36857	0.02482	0.00103	-4.25	-0.7114	-0.527	TLR4	toll like receptor 4
622	38290	0.02532	0.00106	-4.23	-0.7417	-0.517		
623	19403	0.02562	0.00108	-4.23	-0.7571	-0.581	TMEM159	transmembrane protein 159
624	24613	0.02574	0.00109	4.22	-0.7625	0.821	IGF1	insulin like growth factor 1
625	19581	0.02579	0.00109	4.22	-0.7649	0.506	ENPP1	ectonucleotide pyrophosphatase/phosphodiesterase 1
626	2433	0.02608	0.00111	4.21	-0.7818	0.605		
627	38869	0.02617	0.00111	4.21	-0.7873	0.646		
628	34718	0.02619	0.00112	-4.21	-0.7885	-0.744		
629	21162	0.02648	0.00114	4.2	-0.8075	0.865	KANK4	KN motif and ankyrin repeat domains 4
630	742	0.02666	0.00115	4.19	-0.819	0.809	FGFR2	fibroblast growth factor receptor 2
631	2535	0.0268	0.00116	-4.19	-0.8287	-0.615	MECOM	MDS1 and EVI1 complex locus
632	35295	0.02743	0.00121	-4.17	-0.8645	-0.7	PTX3	pentraxin 3
633	36836	0.02745	0.00121	-4.17	-0.8665	-0.516	ARHGEF28	Rho guanine nucleotide exchange factor 28
634	28158	0.02804	0.00124	-4.15	-0.8953	-0.641		
635	22504	0.02826	0.00126	-4.14	-0.9088	-0.702	CLDN14	claudin 14
636	8229	0.02827	0.00126	4.14	-0.9113	0.613	WWC1	WW and C2 domain containing 1
637	13979	0.02831	0.00127	4.14	-0.9151	0.559	CRIP1	cysteine rich protein 1
638	18844	0.02833	0.00127	4.14	-0.9179	0.703	IGF1	insulin like growth factor 1
639	8407	0.02846	0.00128	-4.13	-0.9273	-0.801	RIMS1	regulating synaptic membrane exocytosis 1
640	9039	0.02864	0.0013	-4.13	-0.9362	-0.574	ADRB2	adrenoceptor beta 2
641	43613	0.02866	0.0013	-4.12	-0.9394	-0.9	CEMIP	cell migration inducing hyaluronan binding protein
642	4647	0.02876	0.00131	-4.12	-0.9436	-0.508	GRB14	growth factor receptor bound protein 14
643	34715	0.02879	0.00131	4.12	-0.9453	0.882	DES	desmin
644	7907	0.02883	0.00131	-4.12	-0.9489	-0.778	CEMIP	cell migration inducing hyaluronan binding protein
645	28377	0.02921	0.00134	-4.11	-0.9685	-0.777	CCDC81	coiled-coil domain containing 81
646	43113	0.02926	0.00134	4.11	-0.9707	0.616	MYOZ3	myozenin 3
647	14802	0.02971	0.00137	-4.1	-0.9896	-0.527	NFIB	nuclear factor I B
648	9521	0.03007	0.0014	4.09	-1.0086	0.567	ITGB3	integrin subunit beta 3
649	30020	0.03097	0.00147	4.06	-1.0617	0.722		
650	22906	0.0316	0.00152	4.04	-1.0898	0.515		
651	28265	0.03164	0.00152	-4.04	-1.0929	-0.701	PTX3	pentraxin 3
652	11400	0.03185	0.00153	-4.03	-1.1017	-0.689	PTX3	pentraxin 3
653	17992	0.03196	0.00154	-4.03	-1.1072	-1.23	IL1B	interleukin 1 beta
654	38608	0.03209	0.00155	-4.03	-1.1132	-0.7	PTX3	pentraxin 3
655	34352	0.03232	0.00157	4.02	-1.1232	0.745	IGF1	insulin like growth factor 1
656	44663	0.03237	0.00157	-4.02	-1.1252	-0.688	PTX3	pentraxin 3
657	22214	0.03244	0.00158	-4.02	-1.1315	-0.696	PTX3	pentraxin 3

658	3056	0.03246	0.00158	4.02	-1.1331	0.596	BTBD11	BTB domain containing 11
659	29783	0.03254	0.00159	-4.02	-1.1362	-0.516		
660	11035	0.03256	0.00159	4.01	-1.1373	0.547	TNC	tenascin C
661	18014	0.0327	0.00161	4.01	-1.1482	0.793	IGF1	insulin like growth factor 1
662	30608	0.03284	0.00163	4	-1.159	0.502	SHROOM2	shroom family member 2
663	28223	0.03284	0.00163	4	-1.1597	0.615	HLA-DPA1	major histocompatibility complex, class II, DP alpha 1
664	17139	0.03287	0.00163	4	-1.1618	0.558	PCDHGB7	protocadherin gamma subfamily B, 7
665	21633	0.03289	0.00163	4	-1.164	0.539	ADAM22	ADAM metallopeptidase domain 22
666	33635	0.03291	0.00164	-4	-1.1662	-0.524	CDH1	cadherin 1
667	24279	0.03339	0.00167	3.99	-1.1854	0.882	FLRT3	fibronectin leucine rich transmembrane protein 3
668	33143	0.03375	0.0017	-3.98	-1.2012	-0.693	PTX3	pentraxin 3
669	18977	0.03378	0.0017	-3.98	-1.2029	-1.11	COL4A4	collagen type IV alpha 4 chain
670	42448	0.034	0.00171	3.97	-1.2102	0.679	HIST1H1B	histone cluster 1, H1b
671	40071	0.03401	0.00172	-3.97	-1.2128	-0.703	PTX3	pentraxin 3
672	6829	0.0343	0.00174	3.97	-1.2278	0.536	FBXO16	F-box protein 16
673	15330	0.03453	0.00176	-3.96	-1.2388	-0.587		
674	29514	0.03454	0.00177	3.96	-1.2401	0.754	TNFRSF19	TNF receptor superfamily member 19
675	15424	0.03454	0.00177	-3.96	-1.2403	-1.19	MYCT1	myc target 1
676	39030	0.03459	0.00177	3.96	-1.2447	0.591		
677	20527	0.03482	0.00179	-3.95	-1.2535	-0.554	ACKR4	atypical chemokine receptor 4
678	43282	0.03483	0.00179	3.95	-1.254	0.574	AIM1	absent in melanoma 1
679	18578	0.03495	0.0018	-3.95	-1.2592	-0.501	GCNT3	glucosaminyl (N-acetyl) transferase 3, mucin type
680	8738	0.03516	0.00182	-3.94	-1.2684	-0.879	SFTA1P	surfactant associated 1, pseudogene
681	9372	0.03531	0.00183	-3.94	-1.2769	-1.27	IL1B	interleukin 1 beta
682	28470	0.03538	0.00184	-3.94	-1.2805	-1.12	SCN3A	sodium voltage-gated channel alpha subunit 3
683	14853	0.03558	0.00186	3.93	-1.2906	0.525	MCHR1	melanin concentrating hormone receptor 1
684	35683	0.03562	0.00187	-3.93	-1.2942	-0.651	TNFRSF11	TNF receptor superfamily member 11b
685	41667	0.03563	0.00187	-3.93	-1.2948	-0.53	ADRB2	adrenoceptor beta 2
686	32463	0.03569	0.00187	3.93	-1.2978	0.595	BCL2L11	BCL2 like 11
687	14283	0.03571	0.00188	3.93	-1.2992	0.525	CD40	CD40 molecule
688	34126	0.03671	0.00195	-3.9	-1.3384	-0.566	ADRB2	adrenoceptor beta 2
689	43171	0.03675	0.00196	3.9	-1.3414	0.737	IGF1	insulin like growth factor 1
690	17980	0.03771	0.00202	-3.88	-1.3744	-1.32	IL1B	interleukin 1 beta
691	39657	0.03796	0.00205	3.88	-1.3846	0.712	CLDN23	claudin 23
692	22217	0.03846	0.00208	3.87	-1.4017	0.751	EYA4	EYA transcriptional coactivator and phosphatase 4
693	6769	0.03859	0.00209	-3.87	-1.4072	-0.672	PTX3	pentraxin 3
694	4806	0.03862	0.0021	-3.86	-1.4098	-0.534	ABLIM3	actin binding LIM protein family member 3
695	43700	0.03862	0.0021	3.86	-1.4104	0.564	ADRA2A	adrenoceptor alpha 2A
696	40081	0.03862	0.0021	-3.86	-1.411	-0.648	TNFRSF11	TNF receptor superfamily member 11b
697	22358	0.03874	0.00211	3.86	-1.416	0.734	IGF1	insulin like growth factor 1
698	15825	0.03874	0.00211	-3.86	-1.4164	-0.799	PSG3	pregnancy specific beta-1-glycoprotein 3
699	44976	0.03899	0.00213	3.86	-1.4242	0.584	CENPI	centromere protein I
700	23947	0.03906	0.00214	-3.86	-1.4269	-0.52	ADRB2	adrenoceptor beta 2
701	16178	0.03916	0.00215	3.85	-1.4312	0.562	NLGN4X	neuroligin 4, X-linked
702	9352	0.03924	0.00215	3.85	-1.4342	0.538	OOEP	oocyte expressed protein
703	39815	0.03941	0.00217	-3.85	-1.4415	-1.23	IL1B	interleukin 1 beta
704	15079	0.03958	0.00218	3.84	-1.449	0.751	SYT13	synaptotagmin 13

705	5358	0.03962	0.00219	-3.84	-1.4515	-0.57	ADRB2	adrenoceptor beta 2
706	43967	0.03973	0.0022	-3.84	-1.4569	-0.544		
707	44325	0.03997	0.00222	-3.83	-1.4664	-0.764	AK5	adenylate kinase 5
708	38038	0.04042	0.00226	-3.83	-1.4807	-1.14	IL1B	interleukin 1 beta
709	3715	0.04113	0.00231	-3.81	-1.5059	-1.3	IL1B	interleukin 1 beta
710	8230	0.04116	0.00232	3.81	-1.5069	0.607		
711	1184	0.04132	0.00233	-3.81	-1.5117	-0.653	TNFRSF11	TNF receptor superfamily member 11b
712	5922	0.04136	0.00233	3.81	-1.5143	0.635	KALRN	kalirin, RhoGEF kinase
713	32653	0.04187	0.00237	3.8	-1.5295	0.549		
714	15188	0.04243	0.00241	-3.79	-1.5475	-1.01		
715	44445	0.04245	0.00242	-3.79	-1.5483	-0.663	PTX3	pentraxin 3
716	35986	0.04248	0.00242	-3.79	-1.5498	-0.581	ADRB2	adrenoceptor beta 2
717	23034	0.0425	0.00242	-3.79	-1.5512	-0.541	NGF	nerve growth factor
718	18106	0.04259	0.00244	3.78	-1.5562	0.777	PLXDC2	plexin domain containing 2
719	30396	0.04277	0.00245	3.78	-1.5612	0.692	NUDT10	nudix hydrolase 10
720	13475	0.04311	0.00248	-3.77	-1.5737	-1.3	IL1B	interleukin 1 beta
721	8425	0.04323	0.00249	-3.77	-1.5784	-0.547	ADRB2	adrenoceptor beta 2
722	20685	0.04325	0.00249	-3.77	-1.5791	-0.629	ADAMTSL	ADAMTS like 4
723	36281	0.04353	0.00252	3.77	-1.5882	0.643	BCL2	BCL2, apoptosis regulator
724	559	0.04375	0.00254	3.76	-1.5959	0.866	PDPN	podoplanin
725	14484	0.0438	0.00254	-3.76	-1.598	-1.2	IL1B	interleukin 1 beta
726	28341	0.0438	0.00254	-3.76	-1.5981	-1.17	IL1B	interleukin 1 beta
727	15942	0.04418	0.00258	-3.75	-1.6118	-0.565	SH2D5	SH2 domain containing 5
728	24394	0.04486	0.00264	-3.74	-1.6356	-0.698		
729	16198	0.04524	0.00267	-3.73	-1.6475	-0.658	TNFRSF11	TNF receptor superfamily member 11b
730	13495	0.04524	0.00268	-3.73	-1.6482	-0.653	TNFRSF11	TNF receptor superfamily member 11b
731	4351	0.04644	0.00278	-3.71	-1.686	-0.501	AMPD3	adenosine monophosphate deaminase 3
732	18922	0.04664	0.0028	-3.71	-1.6932	-0.652	TNFRSF11	TNF receptor superfamily member 11b
733	34180	0.04667	0.00281	-3.71	-1.6955	-0.517	ADRB2	adrenoceptor beta 2
734	24322	0.04667	0.00281	3.71	-1.6957	0.601	HTRA3	HtrA serine peptidase 3
735	15278	0.04694	0.00284	-3.7	-1.706	-0.658	TNFRSF11	TNF receptor superfamily member 11b
736	30190	0.04702	0.00285	3.7	-1.7091	0.538	RNF180	ring finger protein 180
737	5938	0.04733	0.00288	3.69	-1.7208	0.894	BRINP1	BMP/retinoic acid inducible neural specific 1
738	25941	0.04742	0.00289	-3.69	-1.7237	-0.579	MASP1	mannan binding lectin serine peptidase 1
739	1082	0.0476	0.0029	3.69	-1.7278	0.516	CD40	CD40 molecule
740	12383	0.04786	0.00293	3.68	-1.7384	0.563	TMEM26	transmembrane protein 26
741	29593	0.04786	0.00294	-3.68	-1.739	-0.535	ADRB2	adrenoceptor beta 2
742	44214	0.0481	0.00295	-3.68	-1.7452	-0.632	TNFRSF11	TNF receptor superfamily member 11b
743	36041	0.04982	0.0031	3.66	-1.7922	1.01		
744	32586	0.04989	0.00311	-3.65	-1.7947	-0.502	SLC7A11	solute carrier family 7 member 11
745	25887	0.04992	0.00311	-3.65	-1.7957	-0.639	TNFRSF11	TNF receptor superfamily member 11b
746	30516	0.05005	0.00312	-3.65	-1.7986	-0.547	DOK5	docking protein 5
747	5871	0.05066	0.00319	3.64	-1.8192	0.532		
748	9824	0.05075	0.0032	-3.64	-1.8219	-0.704		
749	5746	0.0508	0.0032	-3.64	-1.8236	-0.753	SLC7A14	solute carrier family 7 member 14
750	154	0.0509	0.00322	-3.63	-1.8294	-0.588	ADRB2	adrenoceptor beta 2
751	33467	0.05149	0.00328	-3.63	-1.8467	-0.53		

752	28020	0.05149	0.00328	-3.63	-1.847	-0.649	MYCT1	myc target 1
753	37307	0.05214	0.00335	3.61	-1.8685	0.562	RUNX1T1	RUNX1 translocation partner 1
754	1112	0.05215	0.00335	-3.61	-1.869	-0.64	TNFRSF11	TNF receptor superfamily member 11b
755	40621	0.05233	0.00337	3.61	-1.8731	0.533	CCDC8	coiled-coil domain containing 8
756	32669	0.05246	0.00338	3.61	-1.8765	0.607	CA12	carbonic anhydrase 12
757	34779	0.05343	0.00349	3.59	-1.9084	0.635	CADM1	cell adhesion molecule 1
758	4997	0.05362	0.00351	-3.59	-1.9149	-0.896	MASP1	mannan binding lectin serine peptidase 1
759	43456	0.05369	0.00352	3.59	-1.9175	0.646	GPR183	G protein-coupled receptor 183
760	14101	0.05386	0.00356	3.58	-1.9269	0.517	C1QTNF4	C1q and tumor necrosis factor related protein 4
761	37088	0.05391	0.00356	-3.58	-1.9286	-0.821		
762	6887	0.05403	0.00357	3.58	-1.9313	0.566	MME	membrane metallo-endopeptidase
763	8084	0.05409	0.00359	3.58	-1.9347	0.531	PTPRN	protein tyrosine phosphatase, receptor type N
764	11173	0.05418	0.0036	3.58	-1.9376	0.533		
765	10896	0.05474	0.00366	3.57	-1.955	0.57		
766	10378	0.05476	0.00367	3.57	-1.9565	0.772	IGDCC4	immunoglobulin superfamily DCC subclass member 4
767	15355	0.0556	0.00375	-3.55	-1.9776	-0.541	TOR4A	torsin family 4 member A
768	357	0.0556	0.00375	3.55	-1.9778	0.752	IGF1	insulin like growth factor 1
769	35491	0.05593	0.00378	3.55	-1.9869	0.566	KCNE3	potassium voltage-gated channel subfamily E regulatory subunit 3
770	10828	0.05668	0.00386	-3.54	-2.0056	-1.25	IL1B	interleukin 1 beta
771	21150	0.05709	0.00391	3.53	-2.0203	0.95	ST6GAL2	ST6 beta-galactoside alpha-2,6-sialyltransferase 2
772	12156	0.05743	0.00394	3.53	-2.0274	0.977	MYF6	myogenic factor 6
773	44565	0.05745	0.00394	3.53	-2.0279	0.54	SUSD3	sushi domain containing 3
774	17322	0.05754	0.00396	3.52	-2.0311	0.513		
775	24733	0.05754	0.00396	-3.52	-2.032	-0.829	NTF3	neurotrophin 3
776	16231	0.05869	0.0041	3.51	-2.0655	0.667	ZNF711	zinc finger protein 711
777	23593	0.05906	0.00414	-3.5	-2.0745	-0.869	CNTNAP3	contactin associated protein-like 3
778	17299	0.05936	0.00417	-3.5	-2.0823	-0.514	LIPH	lipase H
779	19220	0.05968	0.00423	-3.49	-2.0956	-0.772	TNFRSF11	TNF receptor superfamily member 11b
780	41241	0.06077	0.00435	3.47	-2.1236	0.533	SOCS2	suppressor of cytokine signaling 2
781	5618	0.06116	0.0044	3.47	-2.1335	0.608	EFCAB1	EF-hand calcium binding domain 1
782	16899	0.06116	0.0044	3.47	-2.1339	0.643	VCAM1	vascular cell adhesion molecule 1
783	43050	0.06128	0.00441	3.47	-2.1366	0.519	RAB27B	RAB27B, member RAS oncogene family
784	4761	0.06144	0.00443	-3.46	-2.142	-0.678	HTR1F	5-hydroxytryptamine receptor 1F
785	23550	0.06156	0.00446	3.46	-2.147	0.578	APOBEC3(	apolipoprotein B mRNA editing enzyme catalytic subunit 3G
786	38574	0.0619	0.00449	3.46	-2.1543	0.66	TXLNB	taxilin beta
787	22143	0.06226	0.00453	-3.45	-2.1635	-0.632	BMPER	BMP binding endothelial regulator
788	27892	0.06305	0.00462	-3.44	-2.1822	-1.15	IL1A	interleukin 1 alpha
789	2223	0.06359	0.00467	3.44	-2.1924	0.604	OLFML2A	olfactomedin like 2A
790	20825	0.06387	0.0047	-3.43	-2.1984	-0.698		
791	29298	0.06468	0.0048	3.42	-2.2195	0.832		
792	40622	0.06488	0.00482	3.42	-2.224	0.916	MYBPH	myosin binding protein H
793	17873	0.06496	0.00483	-3.42	-2.2264	-0.574	TGFA	transforming growth factor alpha
794	23767	0.06532	0.00489	3.41	-2.2368	0.56	TLR5	toll like receptor 5
795	44377	0.06591	0.00496	-3.4	-2.2512	-0.716	CNTNAP3	contactin associated protein-like 3
796	36633	0.06626	0.005	3.4	-2.2583	0.724	DDX43	DEAD-box helicase 43
797	14876	0.06691	0.00507	3.39	-2.2734	0.519	BCL2	BCL2, apoptosis regulator
798	14196	0.06696	0.00508	3.39	-2.2749	0.527	MME	membrane metallo-endopeptidase

799	11832	0.06716	0.00511	3.39	-2.2801	0.773	COL11A1	collagen type XI alpha 1 chain
800	31972	0.06725	0.00512	3.39	-2.2826	0.736		
801	37799	0.06792	0.00522	3.38	-2.3007	0.567	GPR85	G protein-coupled receptor 85
802	17648	0.06886	0.00534	3.37	-2.3238	0.554	TSPAN11	tetraspanin 11
803	29627	0.06915	0.00539	-3.36	-2.3316	-1.21	IL1A	interleukin 1 alpha
804	2136	0.06915	0.00539	-3.36	-2.3319	-0.617		
805	7127	0.0697	0.00545	3.35	-2.3437	0.585	HPSE	heparanase
806	18950	0.06999	0.00549	-3.35	-2.3499	-0.957	SAMD3	sterile alpha motif domain containing 3
807	27722	0.07037	0.00554	3.35	-2.3586	0.568	KIF24	kinesin family member 24
808	40595	0.07088	0.00561	3.34	-2.3704	0.75	LMNB1	lamin B1
809	5059	0.07137	0.00566	-3.34	-2.3792	-0.865	GRIK2	glutamate ionotropic receptor kainate type subunit 2
810	42850	0.07145	0.00567	-3.33	-2.3816	-0.825		
811	3337	0.07145	0.00567	-3.33	-2.3819	-1.15	IL1A	interleukin 1 alpha
812	31339	0.07154	0.00568	-3.33	-2.3839	-0.534	AHNAK2	AHNAK nucleoprotein 2
813	28055	0.07228	0.00578	3.32	-2.4006	0.5	SOX11	SRY-box 11
814	16274	0.07228	0.00579	3.32	-2.4016	0.559	PRKCQ-A!	PRKCQ antisense RNA 1
815	12517	0.0727	0.00584	3.32	-2.4109	0.825	PLXDC1	plexin domain containing 1
816	5506	0.07291	0.00587	-3.32	-2.415	-0.689	CNTN3	contactin 3
817	999	0.07421	0.00604	-3.3	-2.4425	-0.51	PSG2	pregnancy specific beta-1-glycoprotein 2
818	34612	0.07799	0.00651	3.26	-2.5157	0.621	IGF1	insulin like growth factor 1
819	22740	0.07805	0.00652	3.26	-2.5167	0.538	SOX11	SRY-box 11
820	11358	0.07833	0.00655	-3.26	-2.5225	-0.537	KRTAP4-9	keratin associated protein 4-9
821	4184	0.07967	0.00673	-3.24	-2.5482	-0.509	COMP	cartilage oligomeric matrix protein
822	40679	0.08019	0.0068	-3.24	-2.5582	-0.535	CD55	CD55 molecule (Cromer blood group)
823	23575	0.0807	0.00686	3.23	-2.5673	0.521		
824	17057	0.0814	0.00695	-3.23	-2.5799	-0.534	SLC6A4	solute carrier family 6 member 4
825	19771	0.08166	0.00699	-3.22	-2.5845	-0.516	ESM1	endothelial cell specific molecule 1
826	9439	0.0823	0.00708	-3.22	-2.5969	-0.51	CMAHP	cytidine monophospho-N-acetylneuraminic acid hydroxylase, pseudogene
827	7653	0.08279	0.00714	3.21	-2.6052	0.541	TMEM150	transmembrane protein 150C
828	14697	0.08367	0.00726	-3.2	-2.6217	-0.706	PTPRB	protein tyrosine phosphatase, receptor type B
829	25251	0.0846	0.00738	3.19	-2.638	0.527	TMEM108	transmembrane protein 108
830	5364	0.08522	0.00749	3.19	-2.652	0.566	ZNF711	zinc finger protein 711
831	3563	0.08694	0.00775	-3.17	-2.6853	-0.527		
832	30214	0.08841	0.00797	3.15	-2.712	0.841	SERPINB3	serpin family B member 3
833	23133	0.08871	0.00801	-3.15	-2.7164	-0.763		
834	9505	0.08876	0.00802	-3.15	-2.7177	-0.619		
835	28165	0.08887	0.00804	-3.15	-2.7203	-0.59	CNTN3	contactin 3
836	5389	0.08943	0.00811	3.14	-2.7282	0.589	CALB2	calbindin 2
837	15985	0.09048	0.00825	3.14	-2.7453	0.533		
838	24341	0.091	0.00833	3.13	-2.7546	0.55	SAMD15	sterile alpha motif domain containing 15
839	19211	0.09167	0.00841	-3.12	-2.7645	-0.592	KRT16	keratin 16
840	10792	0.09208	0.00849	3.12	-2.7732	0.54	HHIPL2	HHIP like 2
841	27925	0.0921	0.00851	3.12	-2.7753	0.57		
842	15193	0.09249	0.00856	-3.12	-2.7809	-0.601	S1PR5	sphingosine-1-phosphate receptor 5
843	8103	0.09287	0.00863	-3.11	-2.7884	-0.559	SEMA3A	semaphorin 3A
844	20461	0.09383	0.00875	-3.1	-2.8025	-0.613	FOXE1	forkhead box E1
845	38511	0.09516	0.00893	3.09	-2.8223	0.505	EIF4E3	eukaryotic translation initiation factor 4E family member 3

846	32636	0.09544	0.00899	-3.09	-2.828	-0.548	MIR7515H	MIR7515 host gene
847	21085	0.09634	0.00915	-3.08	-2.845	-0.698	FAM43A	family with sequence similarity 43 member A
848	17822	0.09701	0.00924	3.08	-2.8551	0.73	PTPN20	protein tyrosine phosphatase, non-receptor type 20
849	28454	0.09703	0.00925	-3.07	-2.8555	-0.858	EPHA5	EPH receptor A5
850	9080	0.09716	0.00927	3.07	-2.8584	0.617	CIITA	class II major histocompatibility complex transactivator
851	26987	0.09716	0.00927	3.07	-2.8584	0.715	CDO1	cysteine dioxygenase type 1
852	12286	0.09723	0.0093	-3.07	-2.8612	-0.945	EREG	epiregulin
853	23687	0.09762	0.00936	-3.07	-2.8672	-0.768	SAA1	serum amyloid A1
854	30340	0.09769	0.00937	3.07	-2.8686	0.548	KALRN	kalirin, RhoGEF kinase
855	26347	0.09781	0.00939	-3.07	-2.8702	-0.682	LOC10050	uncharacterized LOC100506544
856	613	0.09839	0.00948	-3.06	-2.8794	-0.574	PTGIS	prostaglandin I2 (prostacyclin) synthase
857	6116	0.09903	0.00959	3.06	-2.8908	0.594	MEOX2	mesenchyme homeobox 2
858	34586	0.1004	0.00985	3.04	-2.916	0.549	PRG4	proteoglycan 4
859	29882	0.10132	0.01	3.03	-2.9307	0.543	IL16	interleukin 16
860	26906	0.10156	0.01	3.03	-2.9334	0.563	KIAA1161	KIAA1161
861	9012	0.10209	0.0101	-3.03	-2.94	-0.766	PDE11A	phosphodiesterase 11A
862	18796	0.10239	0.0101	3.03	-2.9452	0.639		
863	13623	0.10252	0.0102	3.02	-2.9481	0.836	CRISPLD2	cysteine rich secretory protein LCCL domain containing 2
864	23857	0.10276	0.0102	-3.02	-2.9548	-0.654	COL4A3	collagen type IV alpha 3 chain
865	1659	0.10389	0.0104	3.01	-2.9699	0.503	FOXR1	forkhead box R1
866	4428	0.10613	0.0108	-2.99	-3.0012	-0.697	UTS2B	urotensin 2B
867	21226	0.10701	0.0109	2.99	-3.013	0.685	LRRC15	leucine rich repeat containing 15
868	37858	0.10706	0.0109	-2.99	-3.0136	-0.676		
869	11415	0.10776	0.011	-2.98	-3.024	-0.665	ANGPTL4	angiopoietin like 4
870	3781	0.10834	0.0111	-2.98	-3.0326	-0.638		
871	28440	0.10958	0.0113	2.97	-3.0487	0.561	LOC37519	uncharacterized LOC375196
872	44354	0.10973	0.0113	2.97	-3.051	0.541	AFF2	AF4/FMR2 family member 2
873	32758	0.11037	0.0114	-2.96	-3.0598	-0.532		
874	27219	0.11086	0.0115	-2.96	-3.0686	-1.18	IL1A	interleukin 1 alpha
875	39853	0.11092	0.0116	-2.96	-3.0699	-0.63	EPHA5	EPH receptor A5
876	40879	0.11128	0.0116	2.95	-3.0745	0.595	CD24	CD24 molecule
877	4847	0.11235	0.0118	-2.95	-3.0888	-0.648	SUN3	Sad1 and UNC84 domain containing 3
878	7868	0.11261	0.0118	2.94	-3.0928	0.685		
879	25933	0.11336	0.0119	-2.94	-3.1018	-0.578		
880	42294	0.11522	0.0123	-2.93	-3.1267	-0.501	BCL7A	BCL tumor suppressor 7A
881	441	0.1163	0.0124	2.92	-3.1391	0.564		
882	27328	0.11739	0.0126	-2.91	-3.152	-0.577	TNFSF4	tumor necrosis factor superfamily member 4
883	31576	0.11744	0.0126	2.91	-3.1536	0.55	TNFRSF19	TNF receptor superfamily member 19
884	4311	0.11792	0.0127	-2.91	-3.16	-0.513	GRAMD1C	GRAM domain containing 1C
885	18208	0.11959	0.0129	2.9	-3.1785	0.586		
886	30401	0.12003	0.013	-2.89	-3.1836	-1.11	IL1A	interleukin 1 alpha
887	41703	0.12018	0.013	-2.89	-3.1865	-0.503	EXPH5	exophilin 5
888	39806	0.12033	0.0131	-2.89	-3.1891	-0.701	GATA6	GATA binding protein 6
889	25971	0.12241	0.0134	-2.88	-3.2132	-0.543	ADD3-AS	ADD3 antisense RNA 1
890	7851	0.12254	0.0134	-2.88	-3.2149	-0.519	FAM129A	family with sequence similarity 129 member A
891	1847	0.12296	0.0135	-2.87	-3.2209	-0.707		
892	34823	0.12412	0.0137	2.87	-3.2341	0.84	TCEAL7	transcription elongation factor A like 7

893	34388	0.12512	0.0139	-2.86	-3.2459	-0.55	AMZ1	archaelysin family metallopeptidase 1
894	36512	0.12533	0.0139	-2.86	-3.2482	-0.518	ADAM23	ADAM metallopeptidase domain 23
895	35751	0.12567	0.014	-2.86	-3.2526	-0.604	SLC16A12	solute carrier family 16 member 12
896	8193	0.12573	0.014	-2.86	-3.2547	-0.552		
897	11668	0.12711	0.0142	2.85	-3.27	0.521	HHIPL2	HHIP like 2
898	42213	0.12843	0.0144	-2.84	-3.2844	-0.569		
899	10857	0.12895	0.0146	-2.83	-3.2915	-0.533	EXPH5	exophilin 5
900	2097	0.13027	0.0148	-2.83	-3.3074	-0.593	PTGIS	prostaglandin I2 (prostacyclin) synthase
901	24685	0.13346	0.0153	-2.81	-3.3422	-0.843	ASPN	asporin
902	16057	0.13522	0.0156	-2.8	-3.3595	-0.646	LOC10050	uncharacterized LOC100506544
903	36867	0.13661	0.0158	2.79	-3.3724	0.648	SNAP25	synaptosome associated protein 25
904	38936	0.13713	0.016	2.79	-3.3789	0.565		
905	9432	0.13755	0.0161	-2.78	-3.3851	-0.53	MEGF6	multiple EGF like domains 6
906	21934	0.1379	0.0161	-2.78	-3.3888	-0.564		
907	43347	0.13809	0.0162	-2.78	-3.391	-0.519	ITGA2	integrin subunit alpha 2
908	41982	0.13835	0.0162	2.78	-3.3942	0.511	PDZD2	PDZ domain containing 2
909	42018	0.13936	0.0164	-2.77	-3.4053	-0.703	ITGA2	integrin subunit alpha 2
910	14533	0.14104	0.0167	-2.76	-3.4221	-0.516	MYPN	myopalladin
911	24676	0.14357	0.0171	2.75	-3.4464	0.796	MMP13	matrix metallopeptidase 13
912	17528	0.14405	0.0172	2.75	-3.4509	0.525	ZCCHC5	zinc finger CCHC-type containing 5
913	30999	0.14405	0.0172	2.75	-3.451	0.508		
914	40861	0.14921	0.0181	-2.72	-3.5018	-0.555		
915	22228	0.14949	0.0182	2.72	-3.5044	0.673	FRMD7	FERM domain containing 7
916	24393	0.15243	0.0188	-2.7	-3.5351	-0.701	GRIK2	glutamate ionotropic receptor kainate type subunit 2
917	28959	0.15349	0.019	2.69	-3.5462	0.746	SORCS2	sortilin related VPS10 domain containing receptor 2
918	24605	0.15731	0.0198	-2.67	-3.5825	-0.692		
919	15817	0.15831	0.02	-2.67	-3.5923	-0.634	PLCL1	phospholipase C like 1
920	17166	0.15966	0.0203	-2.66	-3.6081	-0.592	SLC7A11	solute carrier family 7 member 11
921	10987	0.15983	0.0204	2.66	-3.6111	0.593	ALX1	ALX homeobox 1
922	34429	0.16158	0.0207	2.65	-3.6265	0.578	DOK6	docking protein 6
923	29764	0.16159	0.0207	2.65	-3.6269	0.505	HLA-DRB4	major histocompatibility complex, class II, DR beta 4
924	7471	0.16166	0.0207	-2.65	-3.6286	-0.519	OSR1	odd-skipped related transciption factor 1
925	34460	0.16366	0.0212	-2.63	-3.6496	-0.571	CNTN3	contactin 3
926	1282	0.16374	0.0212	-2.63	-3.6507	-0.849	SULT1B1	sulfotransferase family 1B member 1
927	20784	0.16488	0.0215	2.63	-3.6615	0.598	PTPRD	protein tyrosine phosphatase, receptor type D
928	11931	0.16538	0.0216	2.63	-3.6663	0.507	SERPINB4	serpin family B member 4
929	27423	0.16613	0.0218	2.62	-3.6736	0.563		
930	9976	0.1696	0.0225	-2.6	-3.7044	-0.971	IL1A	interleukin 1 alpha
931	29715	0.16992	0.0226	-2.6	-3.7077	-0.513	MAGEA1	MAGE family member A1
932	41802	0.17207	0.023	2.59	-3.726	0.702	TCEAL7	transcription elongation factor A like 7
933	8427	0.17259	0.0231	-2.59	-3.7308	-0.659	LSAMP	limbic system-associated membrane protein
934	30448	0.17842	0.0244	2.56	-3.7804	0.642	KCNS3	potassium voltage-gated channel modifier subfamily S member 3
935	20576	0.17872	0.0244	-2.56	-3.7826	-0.535	HTR2A	5-hydroxytryptamine receptor 2A
936	30681	0.18019	0.0247	2.55	-3.7947	0.621	PLXDC1	plexin domain containing 1
937	15231	0.18055	0.0248	-2.55	-3.7976	-0.515	SLC7A14	solute carrier family 7 member 14
938	36291	0.18199	0.0252	-2.54	-3.8111	-1.05	IL1A	interleukin 1 alpha
939	7016	0.18294	0.0254	2.54	-3.8215	0.874	ITGA8	integrin subunit alpha 8

940	18164	0.18379	0.0257	-2.53	-3.8297	-0.548	CCL2	C-C motif chemokine ligand 2
941	36422	0.18384	0.0257	2.53	-3.8301	0.532	JAM2	junctional adhesion molecule 2
942	37662	0.18503	0.026	2.53	-3.8411	0.509	DNAH10	dynein axonemal heavy chain 10
943	22473	0.18705	0.0264	-2.52	-3.8577	-0.542	CCL2	C-C motif chemokine ligand 2
944	44335	0.18998	0.0271	-2.5	-3.8797	-0.535	CCL2	C-C motif chemokine ligand 2
945	35227	0.19059	0.0272	-2.5	-3.8844	-0.547	CCL2	C-C motif chemokine ligand 2
946	32704	0.19062	0.0272	2.5	-3.8847	0.714	CHI3L1	chitinase 3 like 1
947	32372	0.19209	0.0276	2.49	-3.8991	0.515	DIRAS3	DIRAS family GTPase 3
948	45034	0.19347	0.028	-2.49	-3.9098	-0.53	CCL2	C-C motif chemokine ligand 2
949	12381	0.19406	0.0281	-2.48	-3.9149	-0.687	ITGA2	integrin subunit alpha 2
950	6315	0.19462	0.0283	-2.48	-3.9201	-0.519	LOC10013	uncharacterized LOC100130476
951	2177	0.19498	0.0283	2.48	-3.9231	0.525	MYF5	myogenic factor 5
952	8879	0.19523	0.0285	-2.48	-3.9269	-0.577	PPP1R14A	protein phosphatase 1 regulatory inhibitor subunit 14A
953	10392	0.19672	0.0288	2.47	-3.9371	0.763	TNFSF11	tumor necrosis factor superfamily member 11
954	30360	0.1987	0.0293	2.46	-3.954	0.538	LINC0057	long intergenic non-protein coding RNA 578
955	27156	0.19912	0.0294	-2.46	-3.9574	-0.511	SLC38A1	solute carrier family 38 member 1
956	34800	0.20028	0.0298	2.45	-3.9691	0.583	MCTP2	multiple C2 and transmembrane domain containing 2
957	23326	0.20034	0.0298	-2.45	-3.9703	-0.586	MFAP5	microfibrillar associated protein 5
958	36853	0.20036	0.0298	2.45	-3.9708	0.535	COL12A1	collagen type XII alpha 1 chain
959	44045	0.20121	0.03	-2.45	-3.9758	-0.514	TRIM58	tripartite motif containing 58
960	8567	0.20147	0.0301	-2.45	-3.9785	-0.534	CCL2	C-C motif chemokine ligand 2
961	29736	0.20301	0.0304	-2.44	-3.9898	-0.533	CCL2	C-C motif chemokine ligand 2
962	24238	0.20398	0.0307	-2.44	-3.9982	-0.527	CCL2	C-C motif chemokine ligand 2
963	13990	0.20416	0.0308	2.43	-4.0003	0.613	CASP8	caspase 8
964	10193	0.20487	0.031	-2.43	-4.0065	-0.534	CCL2	C-C motif chemokine ligand 2
965	9218	0.20527	0.0311	-2.43	-4.009	-1.12	IL1A	interleukin 1 alpha
966	11440	0.20531	0.0311	2.43	-4.0096	0.607	NTRK2	neurotrophic receptor tyrosine kinase 2
967	25257	0.20794	0.0319	2.42	-4.0327	0.668		
968	9590	0.21542	0.0337	2.39	-4.0854	0.833	CRISPLD2	cysteine rich secretory protein LCCL domain containing 2
969	42866	0.21595	0.0339	-2.38	-4.0892	-0.517	CCL2	C-C motif chemokine ligand 2
970	15184	0.21838	0.0345	-2.37	-4.1053	-0.517	NRXN3	neurexin 3
971	44653	0.22	0.035	-2.37	-4.1187	-0.59	LSAMP	limbic system-associated membrane protein
972	42723	0.22111	0.0353	-2.36	-4.129	-1.07	IL1A	interleukin 1 alpha
973	23101	0.22222	0.0356	-2.36	-4.1367	-0.666	ITGA2	integrin subunit alpha 2
974	2242	0.22297	0.0358	2.35	-4.1421	0.589	IGF1	insulin like growth factor 1
975	21620	0.22305	0.0359	-2.35	-4.1426	-0.546	ARPP21	cAMP regulated phosphoprotein 21
976	541	0.22446	0.0362	-2.35	-4.1523	-0.59	PPP4R4	protein phosphatase 4 regulatory subunit 4
977	21883	0.2257	0.0367	-2.34	-4.1632	-0.612	SLC38A1	solute carrier family 38 member 1
978	26269	0.2257	0.0367	2.34	-4.1633	0.842	CPXM2	carboxypeptidase X, M14 family member 2
979	33564	0.22694	0.0371	2.33	-4.1728	0.553	GAS1	growth arrest specific 1
980	39393	0.22698	0.0371	-2.33	-4.1737	-0.517	COMP	cartilage oligomeric matrix protein
981	43852	0.22808	0.0376	-2.33	-4.1853	-0.545	MAGEC2	MAGE family member C2
982	16713	0.23151	0.0386	-2.31	-4.2116	-0.616	CSTA	cystatin A
983	206	0.2331	0.0392	-2.3	-4.2243	-0.536	CXCL2	C-X-C motif chemokine ligand 2
984	44740	0.23569	0.04	-2.29	-4.2437	-0.72	TM4SF20	transmembrane 4 L six family member 20
985	31313	0.23583	0.0401	-2.29	-4.2452	-0.536	TSLP	thymic stromal lymphopoietin
986	36312	0.24287	0.0422	-2.26	-4.2928	-0.537	MCTP1	multiple C2 and transmembrane domain containing 1

987	2120	0.24308	0.0423	-2.26	-4.2945	-0.649	TRH	thyrotropin releasing hormone
988	16365	0.24972	0.0443	-2.24	-4.3375	-0.543	PGM5-AS	PGM5 antisense RNA 1
989	11866	0.25396	0.0454	-2.22	-4.3605	-0.635	PDE11A	phosphodiesterase 11A
990	2714	0.25404	0.0454	-2.22	-4.3613	-0.503		
991	6555	0.25533	0.0459	-2.22	-4.371	-0.549	CDH10	cadherin 10
992	7644	0.25613	0.0463	-2.21	-4.3787	-0.709	CXCL8	C-X-C motif chemokine ligand 8
993	14571	0.25645	0.0464	-2.21	-4.3809	-0.514	LSAMP	limbic system-associated membrane protein
994	9047	0.25768	0.0469	-2.2	-4.3906	-0.671	LMO2	LIM domain only 2
995	32430	0.26627	0.0499	2.17	-4.4468	0.619	CRISPLD2	cysteine rich secretory protein LCCL domain containing 2
996	21754	0.26627	0.0499	2.17	-4.4469	0.61	COL3A1	collagen type III alpha 1 chain

SI9_Original blots

Figure S13

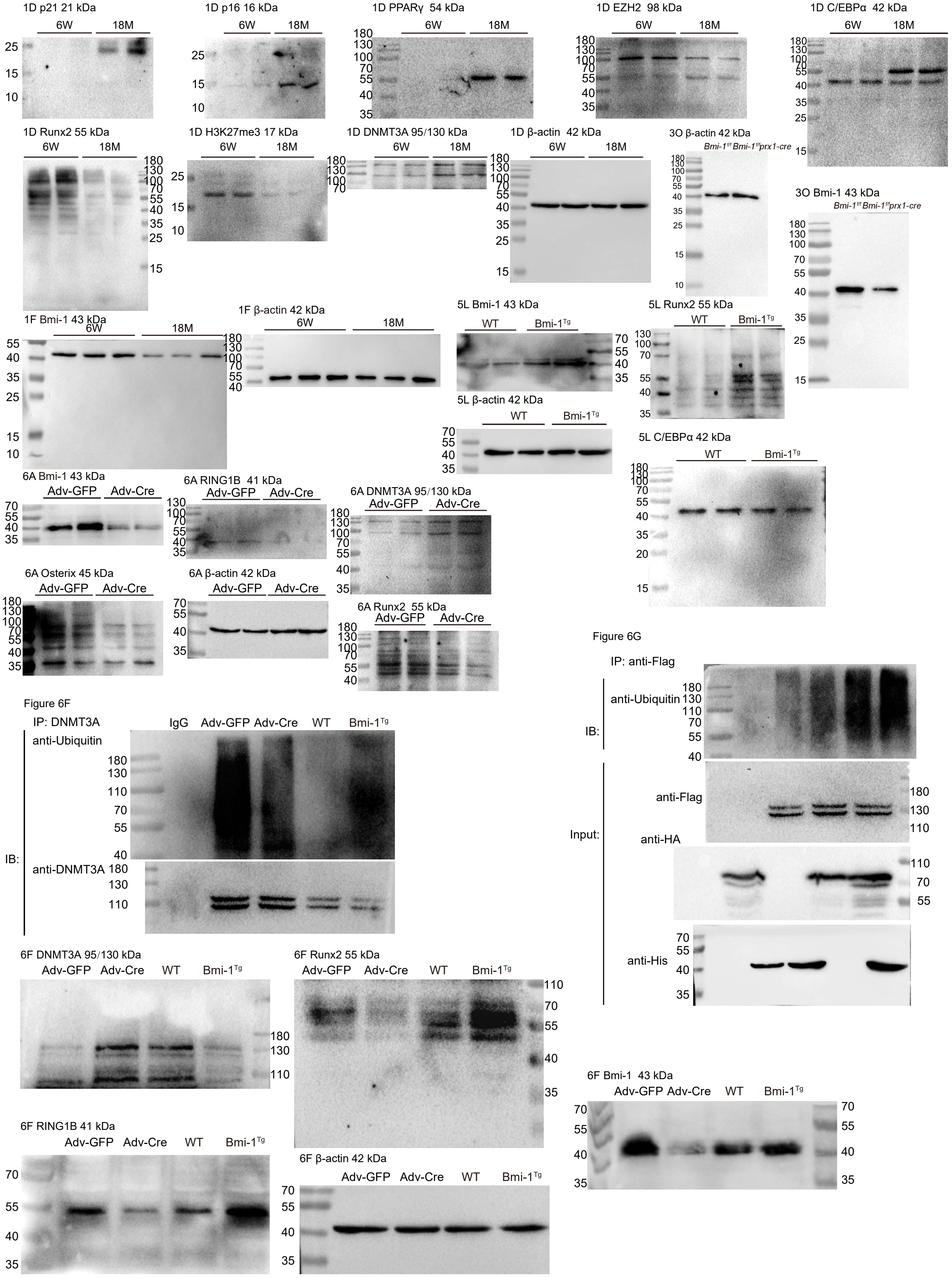

Figure S14

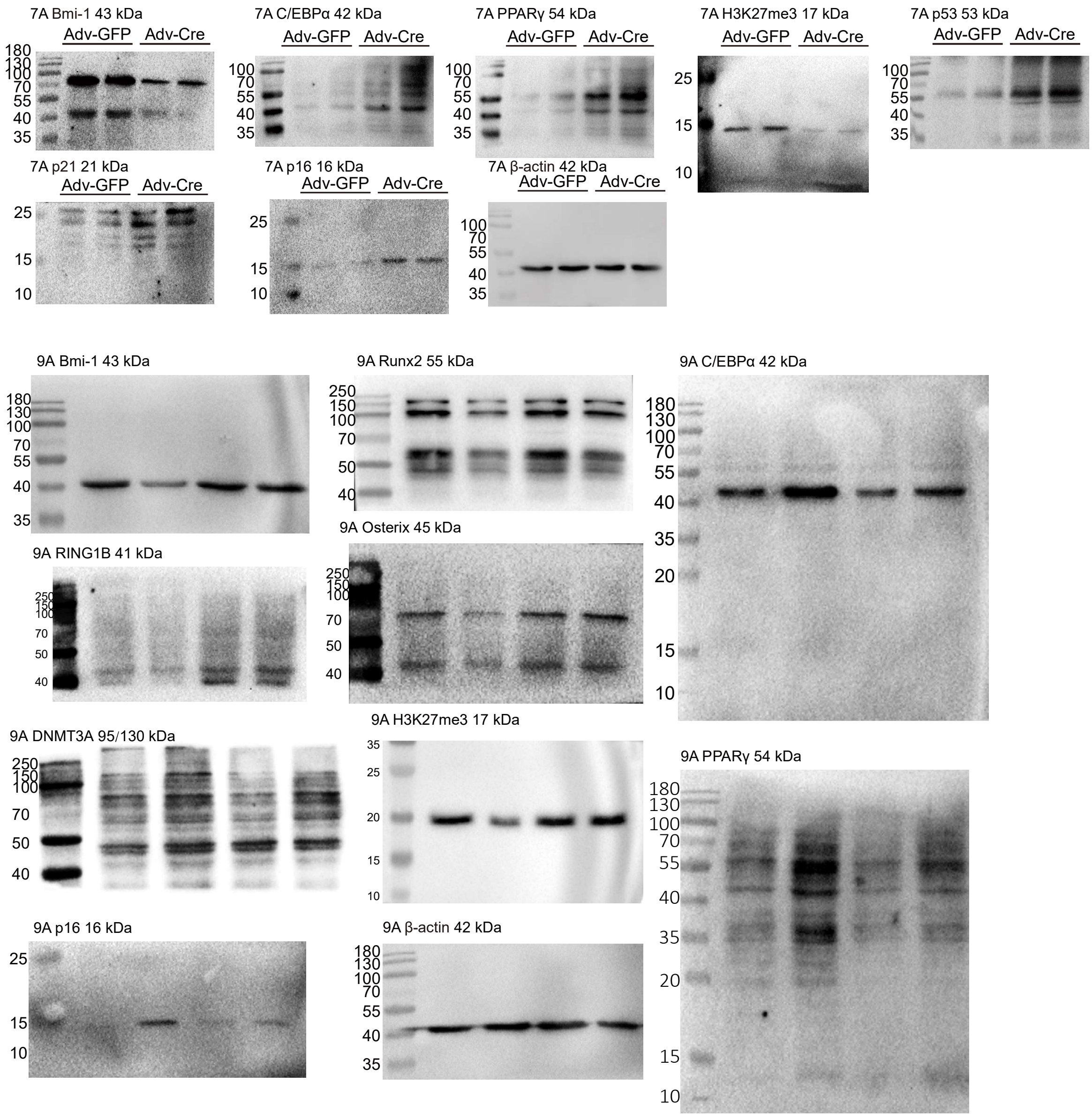
7A Bmi-1 43 kDa
Adv-GFP
Adv-Cre
7A C/EBPα 42 kDa
Adv-GFP
Adv-Cre
7A PPARγ 54 kDa
Adv-GFP
Adv-Cre
7A H3K27me3 17 kDa
Adv-GFP
Adv-Cre
7A p53 53 kDa
Adv-GFP
Adv-Cre
7A p21 21 kDa
Adv-GFP
Adv-Cre
7A p16 16 kDa
Adv-GFP
Adv-Cre
7A β-actin 42 kDa
Adv-GFP
Adv-Cre
9A Bmi-1 43 kDa
9A Runx2 55 kDa
9A C/EBPα 42 kDa
9A RING1B 41 kDa
9A Osterix 45 kDa
9A DNMT3A 95/130 kDa
9A H3K27me3 17 kDa
9A PPARγ 54 kDa
9A p16 16 kDa
9A β-actin 42 kDa

Figure S15

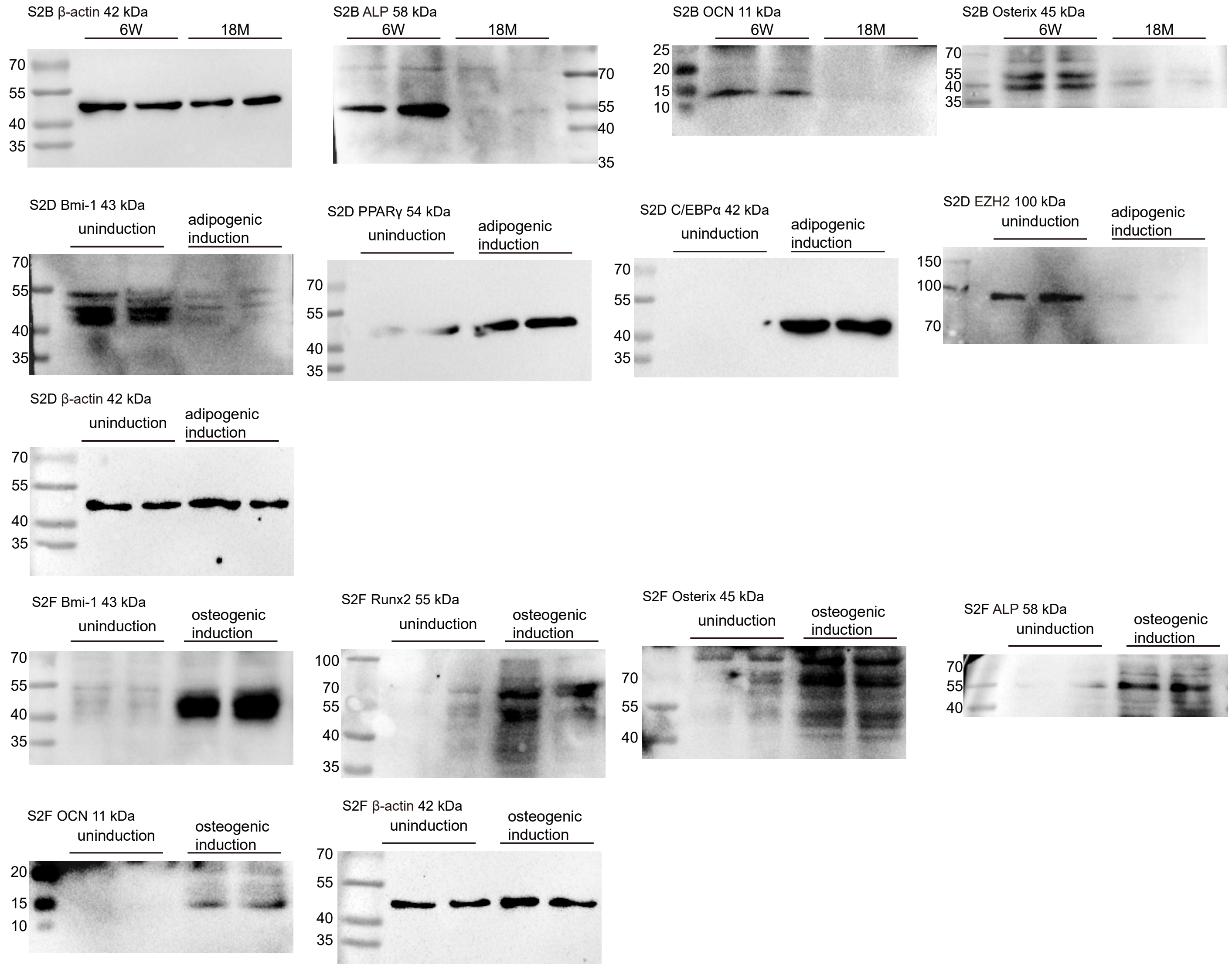

S2B β-actin 42 kDa
6W
18M
70
55
40
35
S2B ALP 58 kDa
6W
18M
70
55
40
35
S2B OCN 11 kDa
6W
18M
25
20
15
10
S2B Osterix 45 kDa
6W
18M
70
55
40
35
S2D Bmi-1 43 kDa
uninduction
adipogenic induction
70
55
40
35
S2D PPARγ 54 kDa
uninduction
adipogenic induction
70
55
40
35
S2D C/EBPα 42 kDa
uninduction
adipogenic induction
70
55
40
35
S2D EZH2 100 kDa
uninduction
adipogenic induction
150
100
70
S2D β-actin 42 kDa
uninduction
adipogenic induction
70
55
40
35
S2F Bmi-1 43 kDa
uninduction
osteogenic induction
70
55
40
35
S2F Runx2 55 kDa
uninduction
osteogenic induction
100
70
55
40
35
S2F Osterix 45 kDa
uninduction
osteogenic induction
70
55
40
S2F ALP 58 kDa
uninduction
osteogenic induction
70
55
40
S2F OCN 11 kDa
uninduction
osteogenic induction
20
15
10
S2F β-actin 42 kDa
uninduction
osteogenic induction
70
55
40
35
